# Design, Synthesis and Molecular Modeling of Pyrazolo[1,5-*a*]pyrimidine Derivatives as Dual Inhibitors of CDK2 and TRKA Kinases with Antiproliferative Activity

**DOI:** 10.3390/ph17121667

**Published:** 2024-12-10

**Authors:** Mohamed H. Attia, Deena S. Lasheen, Nermin Samir, Azza T. Taher, Hatem A. Abdel-Aziz, Dalal A. Abou El Ella

**Affiliations:** 1 Department of Pharmaceutical Chemistry, Faculty of Pharmacy, October 6 University (O6U), Giza 12585, Egypt; 2 Pharmaceutical Chemistry Department, Faculty of Pharmacy, Ain Shams University, Cairo 11566, Egypt; deenalasheen@pharma.asu.edu.eg (D.S.L.); nerminsamir@pharma.asu.edu.eg (N.S.); 3 Department of Pharmaceutical Organic Chemistry, Faculty of Pharmacy, Cairo University, Cairo 11562, Egypt; azza.shalaby@pharma.cu.edu.eg; 4 Department of Pharmaceutical Organic Chemistry, Faculty of Pharmacy, October 6 University (O6U), Giza 12585, Egypt; 5 Department of Applied Organic Chemistry, National Research Center, Cairo 12622, Egypt; ha.abdel-aziz@nrc.sci.eg; 6 Department of Pharmaceutical Chemistry, Faculty of Pharmacy, Pharos University in Alexandria, Canal El Mahmoudia St., Alexandria 21648, Egypt

**Keywords:** synthesis, pyrazolo[1,5-*a*]pyrimidine, anticancer activity, TRKA, CDK2

## Abstract

Background: The increasing prevalence of drug resistance in cancer therapy underscores the urgent need for novel therapeutic approaches. Dual enzyme inhibitors, targeting critical kinases such as CDK2 and TRKA, represent a promising strategy. The goal of this investigation was to design, synthesize, and evaluate a set of pyrazolo[1,5-*a*]pyrimidine derivatives for their dual inhibition potential toward CDK2 and TRKA kinases, along with their potential antiproliferative against cancer cell lines. Methods: A set of pyrazolo[1,5-*a*]pyrimidine derivatives (**6a**–**t**, **11a**–**g**, and **12**) was synthesized and subjected to in vitro enzymatic assays to determine their inhibitory activity against CDK2 and TRKA kinases. Selected compounds were further assessed for antiproliferative effects across the set of 60 cell lines from the NCI, representing various human cancer types. Additionally, simulations of molecular docking were conducted to explore the modes of binding for the whole active compounds and compare them with known inhibitors. Results: Compounds **6t** and **6s** exhibited potent dual inhibitory activity, showing an IC_50_ = 0.09 µM and 0.23 µM against CDK2, and 0.45 µM against TRKA, respectively. These results were comparable to reference inhibitors ribociclib (CDK2, IC_50_ = 0.07 µM) and larotrectinib (TRKA, IC_50_ = 0.07 µM). Among the studied derivatives, compound **6n** displayed a notable broad-spectrum anticancer activity, achieving a mean growth inhibition (GI%) of 43.9% across 56 cell lines. Molecular docking simulations revealed that the synthesized compounds adopt modes of binding similar to those of the lead inhibitors. Conclusions**:** In this study, prepared pyrazolo[1,5-*a*]pyrimidine derivatives demonstrated significant potential as dual CDK2/TRKA inhibitors, and showed potent anticancer activity toward diverse cancer cell lines. These findings highlight their potential as key compounds for the design of novel anticancer therapeutics.

## 1. Introduction

Protein kinases are essential enzymes that modulate cellular processes by catalyzing the phosphorylation of certain proteins, hence affecting pathways related to cell growth, differentiation, and apoptosis [1]. Selectivity in protein kinase activity is crucial for ensuring accurate cellular responses, as non-specific kinase inhibition may result in adverse consequences and toxicity [2]. Attaining selective inhibition of individual kinases is essential for the development of therapeutic medicines, especially in cancer, where certain kinases exhibit overactivity or mutations [3]. Selective kinase inhibitors demonstrate potential in targeted therapy, providing enhanced efficacy and less side effects relative to the non-selective [2]. Dual kinase inhibitors provide a significant advantage by simultaneously targeting two critical pathways, effectively obstructing cancer’s alternative survival mechanisms. This approach mitigates resistance and diminishes the necessity for multiple medications, resulting in more robust, enduring outcomes with fewer adverse effects [4].

CDK2 is a crucial cell cycle advancement regulator, particularly in phase G1/S transition and DNA replication. It establishes with cyclin E an active complex in the G1 phase also the S phase with cyclin A in the cell cycle. These complexes phosphorylate and activate essential substrates that propel the cell cycle and guarantee precise DNA replication. CDK2 deregulation has been noted in many malignancies. Altered expression levels, genetic alterations, and aberrant activation of CDK2 have been documented in multiple malignancies, including breast cancer and lung cancer [5].

Numerous studies have underscored the significance of CDK2 in oncogenesis and tumor progression, resulting in a heightened interest in the creation of CDK2 inhibitors as prospective anticancer therapeutics. Inhibition of CDK2 and its associated cyclins has displayed promising findings in preclinical investigations and clinical trials, establishing them as a novel category of therapeutic drugs for cancer treatment [6].

**Figure 1:**
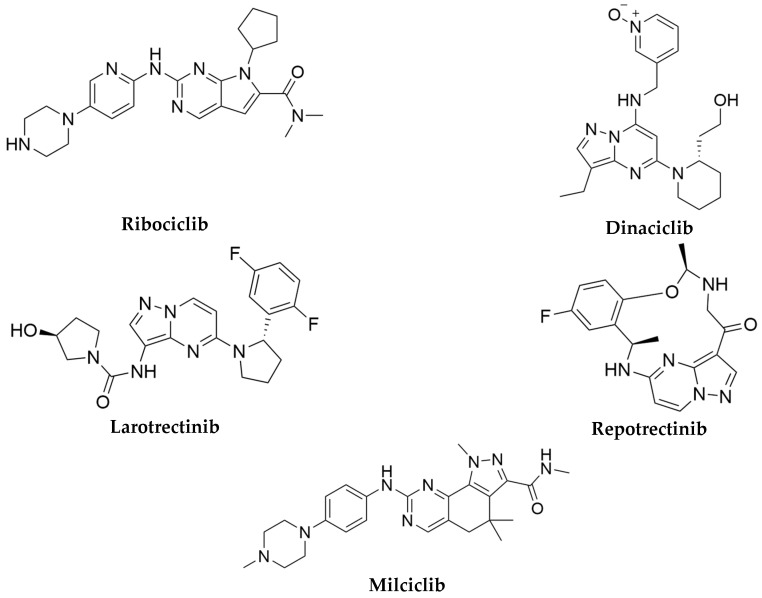
Chemical structures of potent CDK2 and TRKA protein inhibitors [7,8,15,16,20].

Ribociclib and dinaciclib (Figure 1) are selective CDK inhibitors, each possessing unique pharmacophores that target the ATP-binding site. Ribociclib is constructed around a pyrrolo[2,3-*d*]pyrimidine core that resembles ATP, maintained by hydrophobic interactions with aromatic groups and van der Waals forces. Crucial hydrogen bonds with active site residues and halogen substituents augment its lipophilicity, thereby obstructing ATP binding and inhibiting several CDKs [7].

Conversely, dinaciclib possesses a pyrazolo[1,5-*a*]pyrimidine core that occupies an ATP-binding site, hence inhibiting multiple CDKs by obstructing ATP. The hydrophobic areas, comprising aromatic rings and alkyl chains, engage with non-polar residues, whilst hydrogen bond donors and acceptors establish essential interactions that augment the selectivity. Dinaciclib occupies a hydrophobic cleft contiguous to the ATP-binding site, facilitated by Van der Waals interactions and halogen atoms that enhance binding affinity. Both compounds efficiently emulate ATP with enhanced binding affinity, serving as significant therapeutic agents in cancer treatment [8].

TRKA is a subgroup of receptor tyrosine kinases, specifically the tropomyosin receptor kinase family [9].

TRKA is extensively manifested in the neurological system, where it is crucial for sensory and sympathetic nervous system formation and sustenance. The interaction between the Nerve Growth Factor (NGF) and TRKA activates their inherent tyrosine kinase activity, concluding in the autophosphorylation of certain tyrosine residues within the receptor. The phosphorylated residues function as sites of docking for several downstream signaling molecules, leading to the initiation of various signaling pathways, for example, the PI3K-Akt and MAPK pathways [10].

In addition to its function in the neurological system, TRKA has been associated with many malignancies. Genetic rearrangements and TRKA activation have been observed in several malignancies, including colon cancer and papillary thyroid carcinoma, resulting in the constitutive activation of TRKA signaling [11]. Moreover, autocrine or paracrine activation of TRKA signaling by neurotrophins has been documented in other malignancies, comprising neuroblastoma, mesothelioma, pancreatic, prostate, ovarian, and breast carcinomas [12,13].

TRKA has emerged as a promising target for therapy in certain malignancies types due to its involvement in malignancies formation and progression. The inhibition of TRKA signaling using certain inhibitors has demonstrated potential in preclinical research and early-phase clinical trials, justifying future exploration of TRKA-targeted medicines in cancer treatment [14].

Larotrectinib and repotrectinib (Figure 1) have been developed to obstruct TRK proteins via their distinct pharmacophores. Both possess a pyrazolo[1,5-*a*]pyrimidin core and utilize hydrophobic interactions, hydrogen bonding, and halogen substitutions to augment binding affinity and specificity for TRK targets. Their efficient competition with ATP in the kinase active site renders them significant therapeutic agents for addressing malignancies associated with NTRK fusions [15,16].

The simultaneous inhibition of CDK2 and TRKA may yield improved anticancer efficacy and may diminish the risk of drug resistance. Derivatives with a pyrazolo[1,5-*a*]pyrimidine core structure have been identified in various studies as CDK2 and TRKA inhibitors, positioning them as promising candidates for anticancer agent development [17,18]. Milciclib (Figure 1) was recognized as an oral agent exhibiting cross-reactivity with other CDKs [19], and demonstrating significant potency against TRKA and TRKC [20].

**Figure 2:**
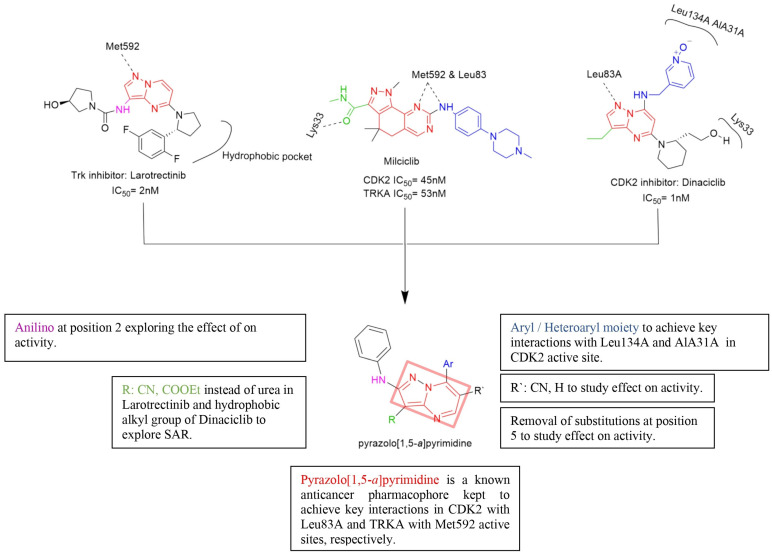
Strategic design of pyrazolo[1,5-*a*]pyrimidine derivatives, focusing on structural optimization to improve biological activity and selectivity for the target.

The design of 2-(anilinyl)pyrazolo[1,5-*a*]pyrimidine **6a**–**t**, **11a**–**g**, and **12** as dual CDK2/TRKA inhibitors was predicated to preserve the pyrazolopyrimidine scaffold of di-naciclib and larotrectinib and facilitate hydrogen bonding with Leu83A of CDK2 and Met592 of TRKA. This involved investigating the influence of anilino at position 2 on the activity and interactions of CN and COOEt at position 3, as opposed to urea in larotrectinib and the hydrophobic alkyl group of dinaciclib to elucidate structure-activity relationships (SAR). Additionally, we investigated the impact of various substituents at positions 3, 6, and 7, while eliminating the substituent at position 5 to explore SAR (Figure 2).

## 2. Results and Discussion

### 2.1. Chemistry

**Scheme 1:**
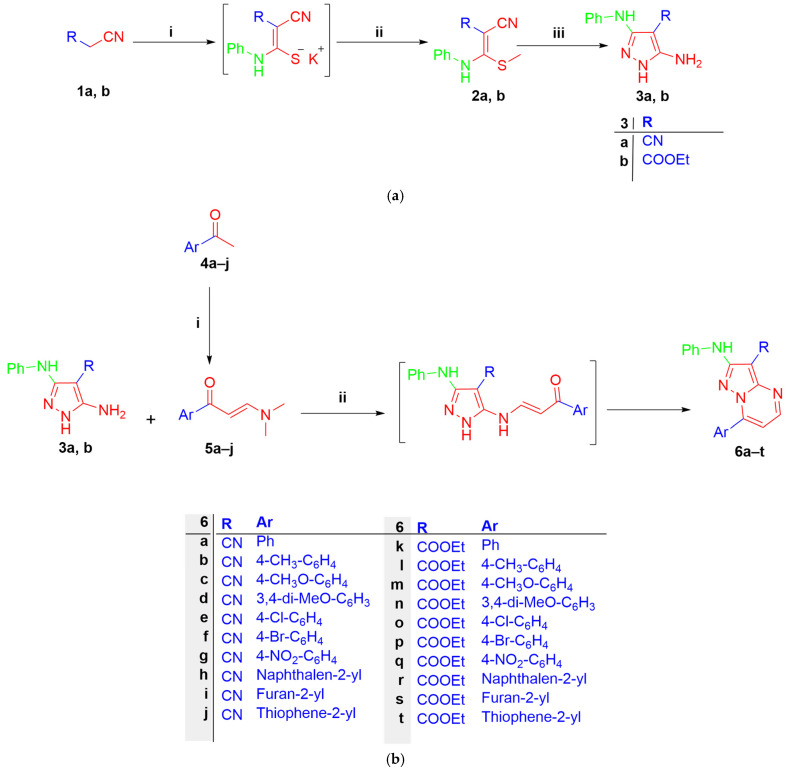
(**a**) (i) Phenylisothiocynate, anhydrous KOH, DMF, rt, 12 h; (ii) methyl iodide, rt, 8 h; (iii) hydrazine hydrate 100%, abs. MeOH, reflux, 3–4 h. (**b**) (i) DMF-DMA, dry xylene, reflux, 8 h; (ii) glacial AcOH, reflux, 3 h.

The primary precursor materials 5-amino-3-(anilinyl)-1H-pyrazole-4-carbonitrile (**3a**) and ethyl 5-amino-3-(anilinyl)-1H-pyrazole-4-carboxylate (**3b**) were constructed via the reaction of malononitrile (**1a**) or ethyl cyanoacetate (**1b**), respectively, with phenylisothiocyanate in the presence of potassium hydroxide resulting in potassium thiolate salt formation. Subsequently, they were reacted with methyl iodide to yield 2-((methylthio)(anilinyl)methylene)malononitrile (**2a**) and (E)-ethyl 2-cyano-3-(methylthio)-3-(anilinyl)acrylate (**2b**), respectively, which were ultimately treated with hydrazine hydrate to produce compounds **3a** and **3b** (Scheme 1a) [21,22].

Conversely, enaminones **5a**–**j** were selected in this investigation for the preparation of pyrazolo[1,5-*a*]pyrimidines **6a**–**t**. Enaminones **5a**–**j** were synthesized via methyl aryl ketones **4a**–**j** condensation with DMF-DMA in refluxing xylene (Scheme 1b) [23,24,25,26]. Subsequently, the reaction of 5-amino-3-(anilinyl)-1H-pyrazole-4-carbonitrile (**3a**) or ethyl 5-amino-3-(anilinyl)-1H-pyrazole-4-carboxylate (**3b**) with **5a**–**j** within glacial acetic acid reflux yielded 2-(anilinyl)-7-(aryl)pyrazolo[1,5-*a*]pyrimidine-3-carbonitrile (**6a**–**j**) or ethyl 2-(anilinyl)-7-(aryl)pyrazolo[1,5-*a*]pyrimidine-3-carboxylate (**6k**–**t**), respectively. This reaction was presumed to initiate a Michael-type addition reaction with the NH2 moiety of **3a** and **3b** on enaminones **5a**–**j** double bond, subsequently followed by dimethylamine elimination resulting in non-isolable intermediates that underwent cyclization through the loss of a single mole of H_2_O to construct pyrazolo[1,5-*a*]pyrimidines **6a**–**t** (Scheme 1b) [27].

**Scheme 2:**
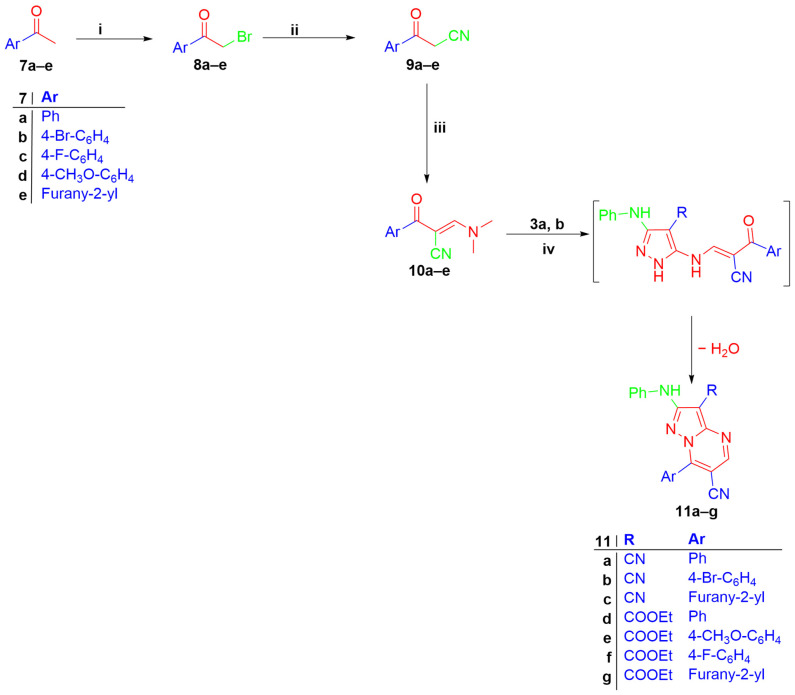
(i) 5% Br_2_, glacial AcOH, rt, 1 h; (ii) KCN, EtOH, H_2_O, rt, 4 h; (iii) DMF-DMA, dry xylene, reflux, 6–10 h; (iv) glacial AcOH, reflux, 3 h.

Brominate aryl aldehydes **7a**–**e** were employed to yield **8a**–**e** [28,29,30,31], which were subsequently transformed into acrylonitrile **10a**–**e** via a substitution process followed by nitrile synthesis [32,33,34]. Moreover, the interaction of **3a** and **3b** with 2-aroyl-3-(dimethylamino)acrylonitrile (**10a**–**e**) in glacial acetic acid under reflux conditions yielded the corresponding pyrazolopyrimidine **11a**–**g**. This reaction was suggested to follow a similar mechanism to the production of compounds **6a**–**t** (Scheme 2) [27].

**Scheme 3:**
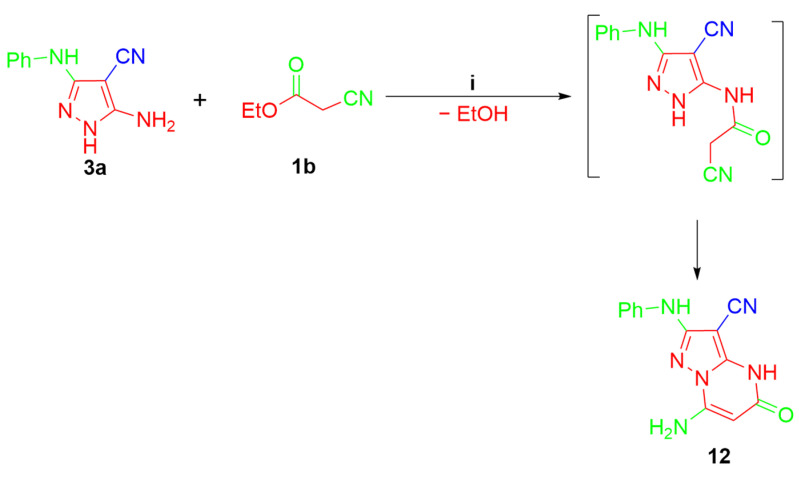
(i) Fusion, 45 min.

The subsequent reaction of ethyl cyanoacetate with compound **3a** was heated to reflux for 45 min yielding 7-amino-2-(anilinyl)-5-oxo-4,5-dihydro-pyrazolo[1,5-*a*]pyrimidine-3-carbonitrile (**12**). Ethyl cyanoacetate reacted with a primary amine, where the nucleophilic attack on the cyano group generated an intermediate. The nearby secondary amine then attacked the ester group intramolecularly, leading to the cyclization and the release of ethanol, forming a heterocyclic structure (**12**) (Scheme 3) [35].

### 2.2. In Vitro Anticancer Activity

#### 2.2.1. Assessment of Anti-Proliferative Activity Across a Diverse Set of 60 Cell Lines

The developed pyrazolopyrimidines **6a**–**t, 11a**–**g**, and **12** were evaluated by the National Cancer Institute (NCI) Developmental Therapeutic Program https://dtp.cancer.gov/ (accessed on 17 July 2022) for their prospective efficacy of growth-inhibitory activity verses 60 different carcinoma cell lines of NCI. Human carcinoma cell lines originated across nine distinct cancer types: central nervous system, cancer of the blood, colorectal, breast, ovary, kidney, melanoma, prostatic, and lung carcinomas. All compounds have been assessed with a concentration of 10 µM versus the collection of 60 diverse cancer cell lines. Outcomes are expressed as percentages of growth inhibition (GI %) for the evaluated compounds versus the complete set of cell lines (Supplementary Materials).

**Figure 3:**
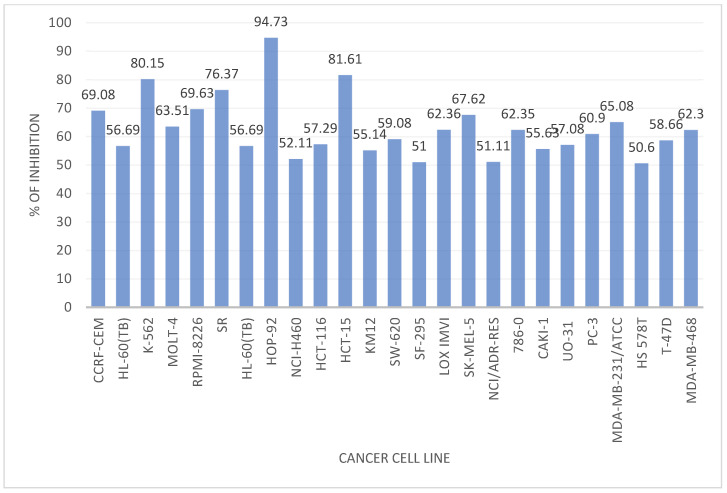
GI % of **6n** against the NCI cell line set at 10 µM. Cell lines exhibiting over 50% growth inhibition are depicted in blue.

Accordingly, compounds **6d**, **6i**, **6k**, **6n**, **6o**, **6p**, and **11g** exhibited the best inhibition against certain cell lines. Compound **6d** showed selective growth inhibition against lung carcinoma HOP-62 (GI % = 100.07) and **6i** showed significant growth inhibition against lung carcinoma HOP-92 (GI % = 68.96). Compound **6k** exhibited potent growth inhibition toward two leukemia cell lines CCRF-CEM (GI % = 90.41) and RPMI-8226 (GI % = 69.31). Compound **6n** exhibited selective growth inhibition against six leukemia cell lines and showed significant activity against two lung carcinoma HOP-92 and NCI-H460 (GI % = 94.73 and 52.11, respectively), with potent inhibition toward colon, melanoma, renal, prostatic, and breast carcinoma cell lines (Figure 3). Compound **6o** showed potent activity toward leukemia cell line CCRF-CEM (GI % = 83.85) and compound **6p** showed significant activity against two lung carcinoma HOP-92 and NCI-H460 (GI % = 71.8 and 66.12, respectively), and renal cancer cell line ACHN (GI % = 66.02). Finally, compound **11g** showed growth inhibition against the central nervous system SNB-75 carcinoma cell line (GI % = 69.53).

**Figure 4:**
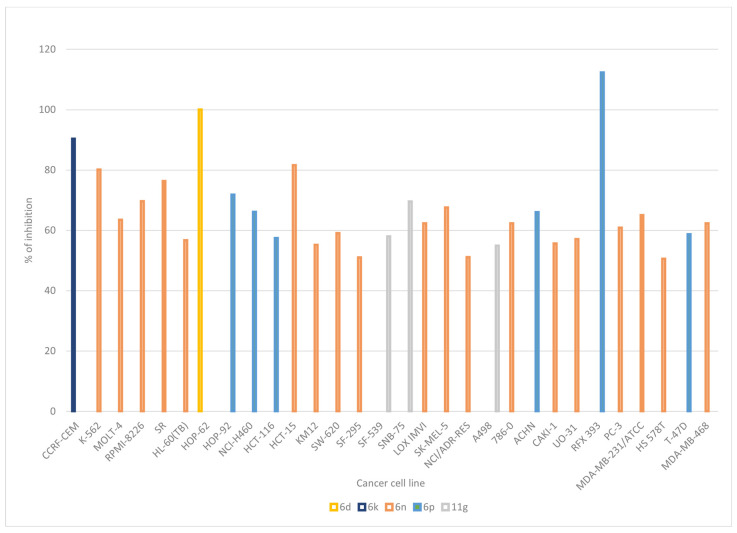
GI % of compounds **6d**, **6k**, **6n**, **6p** and **11g** against 23 NCI cell lines that showed inhibition % above 50%.

Additionally, compounds **6d**, **6m**, **6o**, **6p**, **6q**, **6r**, and **6t** showed remarkable growth inhibition against kidney carcinoma cell line RFX 393 with GI % = 84.17, 102.68, 110.55, 112.34, 52.08, 81.71, and 106.08, respectively (Figure 4).

#### 2.2.2. Kinase Inhibition

**Figure 5:**
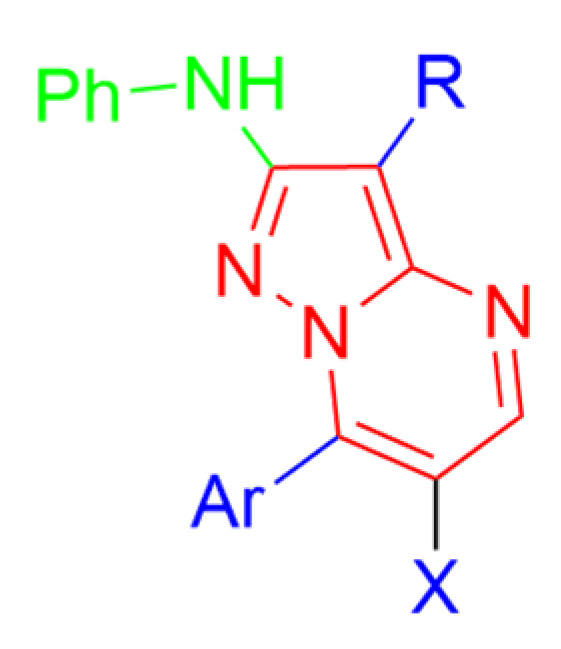
General structure of synthesized compounds **6a**–**t**, **11a**–**g**, and **12**.

**Figure 6:**
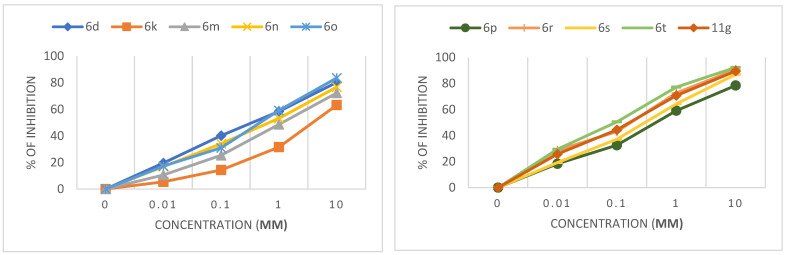
Graphical representation of the IC_50_ determination for compounds **6d**, **6k**, **6m**–**p**, **6r**, **6t**, and **11g** in inhibiting CDK2 activity. Four distinct concentrations, varying from 0 to 10 µM were tested. The data, presented as percentage inhibition, are expressed as mean values ± standard deviation obtained through a minimum of three separate assessments.

**Figure 7:**
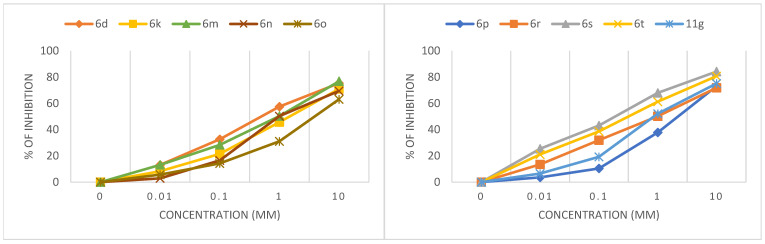
Graphical representation of the IC_50_ determination for compounds **6d**, **6k**, **6m**–**p**, **6r**, **6t**, and **11g** in inhibiting TRKA activity. Four distinct concentrations, varying from 0 to 10 µM were tested. The data, presented as percentage inhibition, are expressed as mean values ± standard deviation obtained through a minimum of three separate assessments.

The CDK2 and TRKA inhibitory effect of the synthesized pyrazolopyrimidines with the highest growth inhibition % of NCI 60 cell lines **6d**, **6k**, **6m**–**p**, **6r**–**t**, and **11g** (Figure 5) were investigated in vitro using ribociclib and larotrectinib as reference drugs for CDK2 and TRKA, respectively. All the studied compounds demonstrated substantial inhibitory efficacy against CDK2 and TRKA, with an IC_50_ range of 0.09–1.58 µM and 0.23–1.59 µM, respectively (Table 1) (Figure 6 and Figure 7). Upon examining the results, we may infer significant information regarding the structure–activity relationship. Initially, on observing the effect of phenyl moiety substitution in compounds **6k**, **6m**, and **6n**, the results mentioned that a dimethoxy group grafting in **6n** (IC_50_ = 0.78 and 0.98 µM) is more beneficial for CDK2 inhibition activity with little decrease in TRKA inhibition activity than the mono-methoxy group at the para position in compound **6m** (IC_50_ =1.06 and 0.96 µM, respectively).

The effect of replacement of COOEt at the 3 position of the pyrazolo ring in compound **6n** (IC_50_ = 0.78 and 0.98 µM on CDK2 and TRKA, respectively) with the cyano group in compound **6d** (IC_50_ = 0.55 and 0.57 µM on CDK2 and TRKA, respectively), resulted in a remarkable increase in the activity.

The substitution of the bromo group on the phenyl group in compound **6p** (IC_50_ = 0.67 and 1.34 µM on CDK2 and TRKA, respectively) is better for activity compared to the chloro group in compound **6o** (IC_50_ = 0.76 and 1.59 µM on CDK2 and TRKA, respectively). The result of the substitution of the pyrazolopyrimidine phenyl moiety in **6k** (IC_50_ = 1.58 and 1.17 µM on CDK2 and TRKA, respectively) with another more lipophilic naphthalene moiety enhanced the efficacy of compound **6r** (IC_50_ = 0.20 and 0.97 µM on CDK2 and TRKA, respectively).

Also, replacing of furan ring in **6s** (IC_50_ = 0.45 and 0.23 µM on CDK2 and TRKA, respectively) with a thiophene ring, generated **6t** (IC_50_ = 0.09 and 0.45 µM on CDK2 and TRKA, respectively) and resulted in a remarkable increase in inhibition activity of CDK2 which was comparable to ribociclib and a reference drug IC_50_= 0.07 µM while a decrease in inhibition activity of TRKA was observed.

Finally, cyano group incorporation at position 6 of the pyrazolopyrimidine ring in compound **6s** (IC_50_ = 0.45 and 0.23 µM on CDK2 and TRKA, respectively) resulted in an increase in CDK2 inhibitory activity in compound **11g** while TRKA inhibitory activity decreased (IC_50_ = 0.22 and 0.89 µM on CDK2 and TRKA, respectively).

#### 2.2.3. Evaluation of In Vitro Cytotoxic Effects on the Renal Carcinoma Cell Line (RFX 393)

Compounds **6s** and **6t** were selected for evaluation due to their superior activity in enzyme inhibition, demonstrating the highest potency among the synthesized pyrazolopyrimidine derivatives.

**Figure 8:**
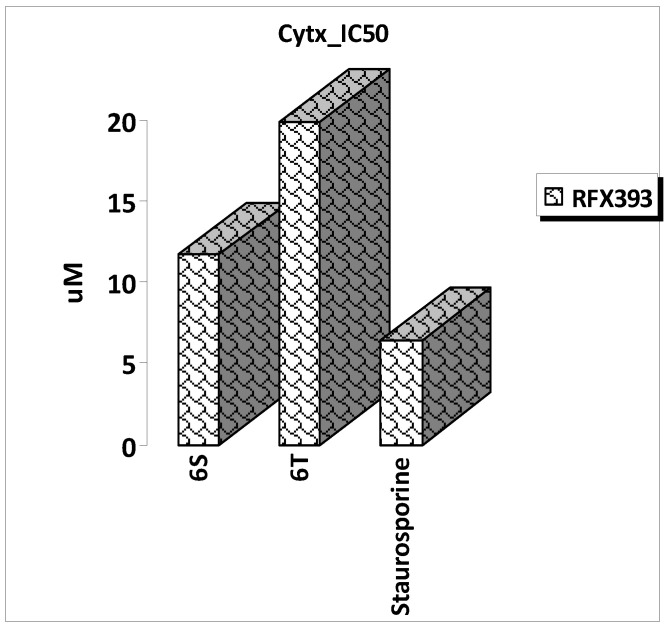
Cytotoxicity of compounds **6s** and **6t** on renal carcinoma cell line (RFX 393).

The in vitro cell growth suppression effects of the developed pyrazolopyrimidine derivatives **6s** and **6t** were evaluated against the renal carcinoma cell line RFX 393, chosen for its high expression of CDK2 and TRKA. Staurosporine served as the reference compound. The outcomes (Table 2) demonstrated moderate inhibition by both compounds in comparison to the reference, and compound **6s** showed greater cytotoxicity than **6t**, exhibiting IC_50_ = 11.70 µM and 19.92 µM, respectively (Figure 8).

#### 2.2.4. Cell Cycle and Apoptosis Investigation

##### Cell Cycle Investigation

**Figure 9:**
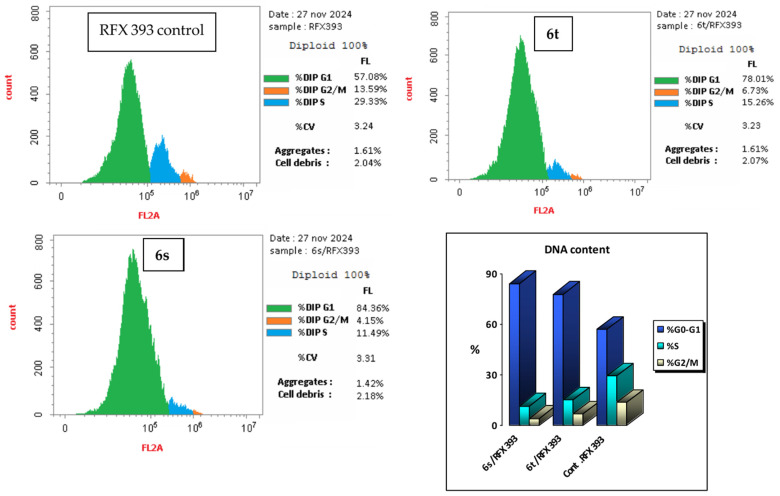
Cell Cycle investigation of compounds 6s and 6t on RFX 393 cell line.

The induction of apoptosis is widely regarded as a highly effective strategy for cancer therapy development. In this study, compounds **6s** and **6t**, which demonstrated enhanced inhibitory effects on CDK2 and TRKA, along with significant cytotoxic activity against RFX 393 cancerous cells, have been tested for their impact on the progression of the cell cycle and induction of apoptosis. RFX 393 cells underwent treatment with pyrazolopyrimidine derivatives **6s** and **6t** at their GI_50_ concentrations for 48 h. The results (Table 3) indicated that both compounds caused notable arrest occurring during the G0–G1 phase of the cell cycle, with treated cell populations increased to 84.36% and 78.01%, respectively, compared to 57.08% in the control group (Figure 9). Furthermore, cell accumulation in the S phase decreased to 11.49% and 15.26%, compared to 29.33% in the control, while in the G2/M phase, it reduced to 4.15% and 6.73%, compared to 13.59% in the control. These findings suggest that compounds **6s** and **6t** exert their cytotoxic effects by inducing cell cycle arrest and promoting apoptosis.

##### Apoptosis and Necrosis Investigation

**Figure 10:**
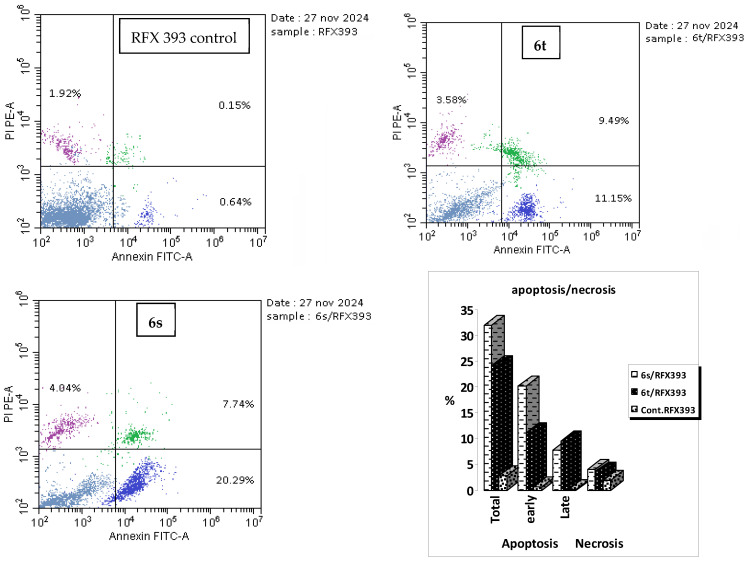
Apoptosis and necrosis investigation of compounds **6s** and **6t** on RFX 393 cell line.

To assess the influence of compounds 6s and 6t on cell death pathways, RFX 393 cells were subjected to the compounds for 48 h and then stained using Annexin V/PI. The analysis results were compared to the control cells, and revealed that both compounds significantly induced early and late apoptosis. Additionally, both 6s and 6t showed higher levels of necrosis relative to the control, further demonstrating their cytotoxic potential. The comprehensive outcomes are provided in Table 4 and graphically illustrated in Figure 10.

#### 2.2.5. Computational Study

**Figure 11:**
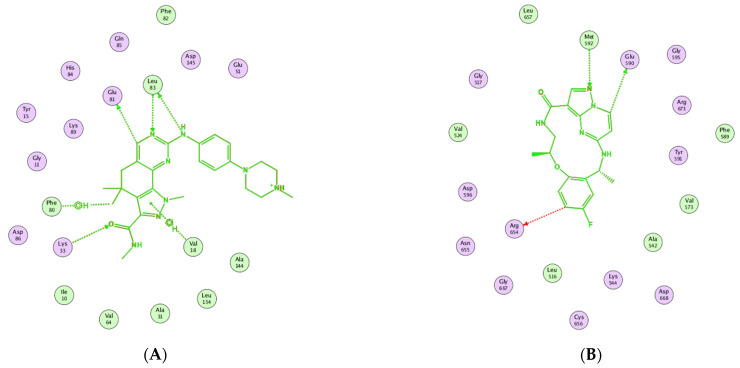
(**A**) The co-crystallized ligand (milciclib) with CDK2 interaction and (**B**) the co-crystallized ligand (repotrectinib) with TRKA interaction.

**Figure 12:**
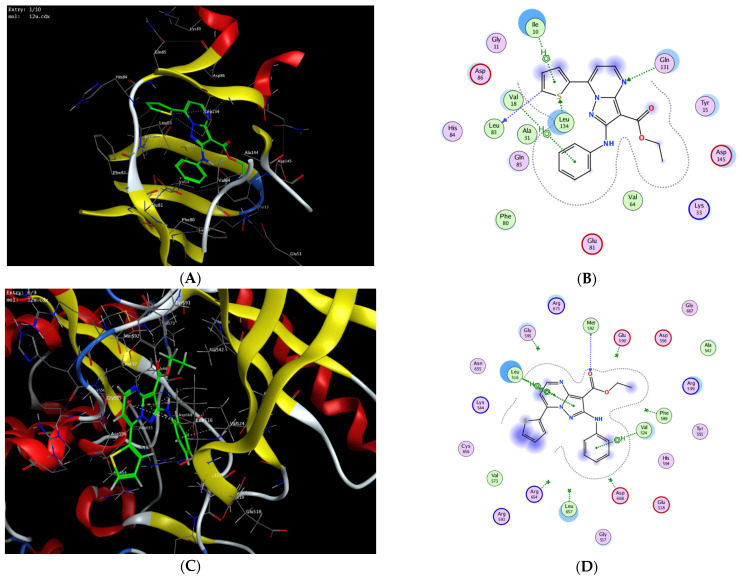
(**A**) Three-dimensional ligand interactions of compound **6t** (green) with CDK2 active site and (**B**) 2D representation, and (**C**) 3D ligand interactions of compound **6t** (green) with TRKA active site and (**D**) 2D representation.

Molecular docking studies are extensively conducted to provide corroborative evidence for the anticancer action of many natural and synthesized compounds [36]. As a result, a molecular docking analysis utilizing (MOE, 2019) Molecular Operating Environment suite was carried out. The research focused on identifying the binding interactions and analyzing the orientation of inhibitors specifically designed for CDK2 and TRKA kinases. Numerous crystal structures of CDK2 and TRKA complexed with different inhibitors have been identified and recorded [19,37,38,39]. CDK2, co-crystallized with milciclib, and TRKA, co-crystallized with repotrectinib, were retrieved based on their structural significance to the expected compounds (PDB codes 2WIH and 7VKO, respectively) from http://www.rcsb.org/ for this experiment. The successful expression of the protein of interest was followed by the identification of its active interaction site, informed by the positional coordinates of the co-crystallized ligand. To validate the docking approach, milciclib and repotrectinib, the co-crystallized ligands, have been re-docked into the active sites for CDK2 and TRKA, respectively. The validation criteria achieved under 2 Å of root mean square deviation (RMSD) was measured in relation to the co-crystallized conformers of milciclib and repotrectinib and their respective re-docked positions, exhibiting similar binding mechanisms. In this study, the validation criteria indicated 0.920 Å and 0.292 Å as RMSD values, along with docking scores of −9.64 kcal/mole and −8.20 kcal/mole for CDK2 and TRKA, respectively. The contact between the co-crystallized ligand and CDK2 entailed two bonds of hydrogen involving the region of the hinge (Leu-83) with the pyrimidine ring nitrogen atom in addition to the NH of anilino. An extra hydrogen bonding interaction has been found between Lys-33 and the oxygen of the amide moiety (Figure 11A). During the contact of the ligand of co-crystallized and TRKA, hydrogen bonding occurred between Met-592 and the nitrogen of the pyrazole ring. Two supplementary hydrogen bonding interactions were identified between Glu-590 and pyrimidine moiety, phenyl moiety and Arg-654 (Figure 11B). The developed derivatives of 2-(anilinyl)pyrazolo[1,5-*a*]pyrimidine **6a**–**t**, **11a**–**g**, and **12** were docked into CDK2 and TRKA binding sites (PDB codes 2WIH and 7VKO, respectively). The newly synthesized compounds exhibited comparable binding interactions and orientation to established CDK2 inhibitors, such as milciclib, and TRKA inhibitors, such repotrectinib, as described in Table 5. Pyrazolopyrimidine **6t** docking results showed it as a highly effective CDK2 inhibitor with moderate TRKA inhibition, where the compound’s capacity engaged with the active site of critical amino acids, hence elucidating its high potency. Figure 12 depicts the docking orientation and configuration of compound **6t** in comparison to milciclib and repotrectinib. Compound **6t** participated in interactions with the essential amino acid Leu-83 of milciclib and with Met-592 of repotrectinib via hydrogen bonding. Hydrophobic interactions between the anilino moiety and Val-18 stabilized the complex, as well as between the thiophene moiety and Ile-10 in CDK2, while in TRKA, stabilization occurred through hydrophobic interactions between the anilino moiety and Val-524, and between the pyrimidine moiety and Leu-516.

#### 2.2.6. Computational ADME Analysis

ADME computer-aided study was conducted utilizing Swiss ADME tool software. This study focuses on the drug chemical structure and uses specific factor calculations, comprising permeation of blood–brain barrier (BBB) and absorption of human gastrointestinal (HIA), both determined through the model of BOILED-Egg for permeability glycoprotein (P-gp) substrates or non-substrates. It also examines cytochromes P450 molecular interactions (CYP) and assesses the score of bioavailability. Furthermore, the ligand’s molecular properties are evaluated according to the surface area total polar (TPSA), five rules of Lipinski, molar refractivity, and the n-octanol and H_2_O partition coefficient log Po/w.

##### ADME Analysis Outcomes

**Figure 13:**
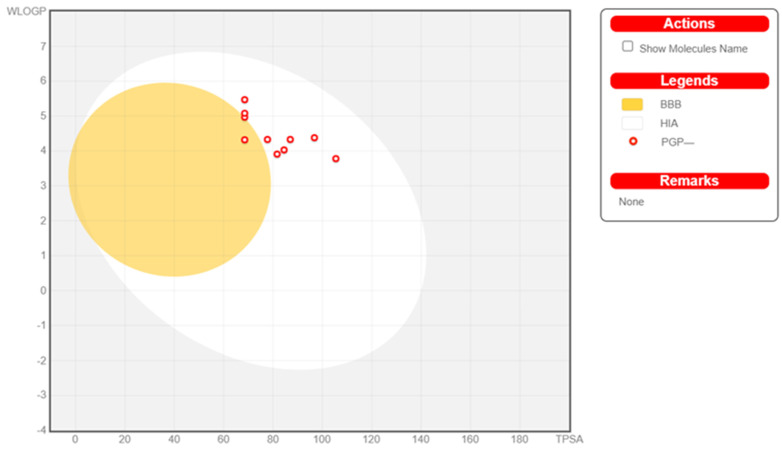
Chart of the BOILED-Egg for the newly developed compounds created by Swiss ADME.

The outcomes were illustrated as a BOILED-Egg, a two-dimensional figure utilizing estimated TPSA and A WLOGP characteristics. Graphs depicting the absorption of the most promising derivatives across the Human Intestinal Absorption (HIA) and blood–brain barrier (BBB) were created (Figure 13).

In the BBB plot, two newly synthesized compounds resided within the yolk, suggesting potential permeability across the blood–brain barrier, while eight compounds were located outside the yolk, indicating probable the blood–brain barrier inability to penetrate. This suggests that the likelihood of side effects on CNS for nine compounds is anticipated to be minimal. All newly developed compounds were located within the white region, indicating that they possess excellent absorption of human intestine in the HIA plot.

The majority of the compounds were determined to be moderate for aqueous solubility.

Most of the compounds were identified as CYP2D6 inhibitors. The parameters derived from the ADME investigation are included in the Supplementary Materials.

#### 2.2.7. Toxicity Prediction

Utilized by the server Protox-II [40] to forecast the ligands toxicological endpoints and organ toxicities, as well as values of their LD50. The linked database https://pubchem.ncbi.nlm.nih.gov/ (accessed on 18 September 2023) of PubChem had been employed to query chemical structures utilizing compound names. Chosen models have been utilized, and the online server selected toxicity objectives and calculated the acute toxicity.

The expected level of toxicity was founded on the six distinct targets associated with drug side effects. The immunotoxicity, hepatotoxicity, cytotoxicity, carcinogenicity, and mutagenicity of the substances were assessed. Compounds **6o** and **6p** were identified as non-carcinogenic; however, only compound **6d** exhibited hepatotoxicity. Additionally, compounds **6d**, **6m**–**n**, and **6q** displayed mutagenicity. The ligands acute toxicity was assessed utilizing the server of PROTOX-II. All compounds having 300 mg/kg LD50 are classified as class 3, except for compound **6t**, having 500 mg/kg LD50 which is classified as class 4.

## 3. Materials and Methods

### 3.1. Chemistry

Melting points (°C, uncorrected) have been recorded utilizing a Stuart melting point apparatus. Infrared spectra (KBr) have been measured with a SHIMADZU FT/IR spectrometer by Shimadzu Corporation, which is based in Kyoto, Japan. NMR spectra were collected on a BRUKER 400 MHz NMR spectrometer, with DMSO-d6 as the solvent. Chemical shifts (*δ*) are stated as parts per million, and coupling constants (*J*) are presented in Hertz. The internal reference was served by TMS, and chemical shifts have been expressed in ppm. All NH protons exchangeable with D_2_O. The ^1^H and ^13^C spectra have been recorded at 400 MHz and 100 MHz, respectively. Electron impact mass spectra have been determined with the Thermo Scientific ISQ LT GC-MS system include Thermo Scientific Dionex™ Chromeleon™ 7.2 CDS (Waltham, MA, USA), utilizing a Sensor Controller input connected to a quadrupole mass analyzer, and data were processed using Thermo X-calibur software v3.0.

#### 3.1.1. Synthesis of 2-((Methylthio)(anilinyl)methylene)malononitrile (**2a**) and (E)-ethyl 2-Cyano-3-(methylthio)-3-(anilinyl)acrylate (**2b**)

Into a well-agitated solution of phenyl isothiocyanate (0.135 g, 2 mmol) in DMF (5 mL), malononitrile was added (1.32 g, 2 mmol) followed by compound **1a** or ethyl cyanoacetate (0.23 g, 2 mmol) **1b** with potassium hydroxide (anhydrous) (0.073 g, 1.3 mmol). The mixture was continuously stirred overnight at ambient temperature. Subsequently, methyl iodide (0.28 g, 2 mmol) or dimethyl sulfate (0.25 g, 2 mmol) were gradually added dropwise, followed by stirring for 8 h producing a yellow crystalline precipitate. The final product was isolated by filtration, which was thoroughly rinsed with ether, and subsequently recrystallized using ethanol to obtain compounds **2a** and **2b**, respectively. Compounds **2a** and **2b** exhibited physical properties consistent with those previously reported. 2-((Methylthio)(anilinyl)methylene)malononitrile (**2a**) was isolated as yellow crystals in 82% yield, mp 180–182 °C [41] and (E)-ethyl 2-cyano-3-(methylthio)-3-(anilinyl)acrylate (**2b**) was isolated as white crystals in 51.52% yield, mp 68–71 °C [22].

#### 3.1.2. 5-Amino-3-(anilinyl)-1H-pyrazole-4-carbonitrile (**3a**) and Ethyl 5-Amino-3-(anilinyl)-1H-pyrazole-4-carboxylate (**3b**)

A methanolic solution (250 mL) of compound **2a** or **2b** (34 mmol) was refluxed for approximately 30 min until fully dissolved, then allowed to cool. Dropwise addition of Hydrazine hydrate (1.6 mL, 34 mmol) was added dropwise under cool conditions for 30 min with continuous stirring, after which it was transferred to a 80 °C water bath for around 3 to 4 h until a solid was generated. The solid was isolated by filtration, thoroughly rinsed with ether, and recrystallized using ethanol to obtain the titled compound **3a** as a whitish–gray crystalline powder in 80% yield, mp 212–214 °C (mp 204–205 °C [21]) and (**3b**) as a white crystalline powder in 80% yield, mp 170 °C [42].

#### 3.1.3. 3-(Dimethylamino)-1-arylprop-2-en-1-ones **5a**–**j** Preparation

A 50 mL mixture of xylene containing 20 mmol of aryl ethanone derivatives **4a**–**j** was prepared and 2.38 g (20 mmol) of DMF-DMA was added followed by heating to reflux for around 7 h (monitored by TLC).

The solvent was evaporated under vacuum and the resulting residue was triturated with 5 mL of diethyl ether, filtered, and washed with cold petroleum ether, yielding products **5a**–**j**, respectively, in their purest form. The physicochemical characteristics of **5a**–**j** are in agreement with those documented in the literature [23,25,43].

#### 3.1.4. Synthesis of 2-(Anilinyl)-7-(aryl)pyrazolo[1,5-*a*]pyrimidine-3-carbonitrile **6a**–**j**

A 25 mL solution of acetic acid containing 10 mmol of the corresponding enaminone (**5a**–**j**) and 10 mmol (1.99 g) of 5-amino-3-(anilinyl)-1H-pyrazole-4-carbonitrile (**3a**) was heated for 3 h at reflux followed by cooling. The resulting product was isolated by filtration, rinsed with ethanol, desiccated, and then purified by crystallization using a dimethylformamide-water mixture to yield the corresponding products **6a**–**j**.

##### 7-Phenyl-2-(anilinyl)pyrazolo[1,5-*a*]pyrimidine-3-carbonitrile (**6a**)

A pale yellow powder, 90% yield, mp 252–254 °C. IR (KBr) *ν*_max_/cm^−1^ 3300 (NH), 2220 (C≡N), 1600 (C=N). ^1^H NMR *δ* 6.95 (t, *J =* 7.5 Hz, 1H, H4 of PhNH), 7.28 (t, *J =* 8.0 Hz, 2H, H3 and H3′ of PhNH), 7.39 (d, *J =* 6.5 Hz, 1H, H3 of pyrimidine), 7.65–7.69 (m, 5H, H2 and H2′ of PhNH and H3, H3′ and H4 of Ph), 8.14–8.17 (m, 2H, H2 and H2′ of Ph), 8.68 (d, *J =* 5.5 Hz, 1H, pyrimidine H2), 9.60 (s, 1H, NH). ^13^C NMR *δ* 68.26 (C4 of pyrazole), 109.85, 118.48 (2C), 122.12, 129.02 (2C), 129.21 (2C), 129.91 (3C), 130.18, 132.13, 140.84, 146.54, 152.27, 152.87, 156.43. MS *m*/*z* (*%*) 311.98 (M^+^, 4.54). Anal. Calcd. for C_19_H_13_N_5_ (311.34): C, 73.30; H, 4.21; N, 22.49; found: C, 73.14; H, 4.39; N, 22.65.

##### 2-(Anilinyl)-7-(p-tolyl)pyrazolo[1,5-*a*]pyrimidine-3-carbonitrile (**6b**)

A yellow powder, 70% yield, mp 273–274 °C. IR (KBr) *ν*_max_/cm^−1^ 3330 (NH), 2220 (C≡N), 1610 (C=N); ^1^H NMR *δ* 2.45 (s, 3H, CH_3_), 6.96 (t, *J =* 14.5 Hz, 1H, H4 of PhNH), 7.30 (t, *J =* 7.0 Hz, 2H, H3 and H3′ of PhNH), 7.35 (d, *J =* 9.5 Hz, 1H, H3 of pyrimidine), 7.44 (d, *J =* 9.5 Hz, 2H, H3 and H3′ of Ar), 7.67 (d, *J =* 9.5 Hz, 2H, H2 and H2′ of PhNH), 8.08 (d, *J =* 7.0 Hz, 2H, H2 and H2′ of Ar), 8.64 (d, *J =* 6.5 Hz, 1H, pyrimidine H2), 9.58 (s, 1H, NH). ^13^C NMR *δ* 21.60 (-CH_3_), 68.26 (C4 of pyrazole), 109.45, 114.04, 118.46 (2C), 121.93, 127.33, 129.18 (2C), 129.56 (2C), 129.92 (2C), 141.00, 142.37, 146.46, 152.39, 152.67, 156.40. MS *m*/*z* (*%*) 326.13 (M^+^ + 1, 11.59), 325.41 (M^+^, 100); Anal. Calcd. for C_20_H_15_N_5_ (325.37): C, 73.83; H, 4.65; N, 21.52; found: C, 74.11; H, 4.88; N, 21.70.

##### 7-(4-Methoxyphenyl)-2-(anilinyl)pyrazolo[1,5-*a*]pyrimidine-3-carbonitrile (**6c**)

A yellow powder, 85% yield, mp 246–247 °C. IR (KBr) *ν*_max_/cm^−1^ 3330 (NH), 2230 (C≡N), 1610 (C=N). ^1^H NMR *δ* 3.90 (s, 3H, OCH_3_), 6.96 (t, *J =* 9.0 Hz, 1H, H4 of PhNH), 7.19 (d, *J =* 9.0 Hz, 2H, H3 and H3′ of Ar), 7.32 (t, *J =* 7.5 Hz, 2H, H3 and H3′ of PhNH), 7.37 (d, *J =* 5.5 Hz, 1H, H3 of pyrimidine), 7.70 (d, *J =* 6.0 Hz, 2H, H2 and H2′ of PhNH), 8.21 (d, *J =* 8.0 Hz, 2H, H2 and H2′ of Ar), 8.62 (d, *J =* 7.0 Hz, 1H, pyrimidine H2), 9.58 (s, 1H, NH). ^13^C NMR *δ* 56.03 (OCH_3_), 67.50 (C4 of pyrazole), 109.11, 114.49 (3C), 118.45 (2C), 122.32, 129.25 (2C), 131.91 (4C), 140.98, 146.37, 152.56, 156.37, 162.63. MS *m*/*z* (*%*) 341.99 (M^+^, 23.77). Anal. Calcd. for C_20_H_15_N_5_O (341.37): C, 70.37; H, 4.43; N, 20.52; found: C, 70.61; H, 4.56; N, 20.81.

##### 7-(3,4-Dimethoxyphenyl)-2-(anilinyl)pyrazolo[1,5-*a*]pyrimidine-3-carbonitrile (**6d**)

A yellow powder, 90% yield, mp 226–227 °C. IR (KBr) *ν*_max_/cm^−1^ 3300 (NH), 2225 (C≡N), 1600 (C=N). ^1^H NMR *δ* 3.85 (s, 3H, OCH_3_), 3.90 (s, 3H, OCH_3_), 6.98 (t, *J =* 7.5 Hz, 1H, H4 of PhNH), 7.21 (d, *J =* 9.0 Hz, 1H, H5 of Ar), 7.30 (t, *J =* 8.0 Hz, 2H, H3 and H3′ of PhNH), 7.41 (d, *J =* 5.0 Hz, 1H, H3 of pyrimidine), 7.73–7.78 (m, 3H, H2 and H2′ of PhNH and H6 of Ar), 7.95 (d, *J =* 2.0 Hz, 1H, H2 of Ar), 8.63 (d, *J =* 7.5 Hz, 1H, pyrimidine H2), 9.59 (s, 1H, NH). ^13^C NMR *δ* 55.93 (OCH_3_), 56.11 (OCH_3_), 67.91 (C4 of pyrazole), 109.20, 111.82, 112.81, 114.09, 118.50 (2C), 122.08, 122.32, 123.86, 129.22 (2C), 140.73, 146.34, 148.38, 151.95, 152.36, 152.51, 156.40. MS *m*/*z* (*%*) 372.39 (M^+^ + 1, 31.28), 371.39 (M^+^, 100). Anal. Calcd. for C_21_H_17_N_5_O_2_ (371.39): C, 67.91; H, 4.61; N, 18.86; O, 8.62; found: C, 68.13; H, 4.75; N, 18.98.

##### 7-(4-Chlorophenyl)-2-(anilinyl)pyrazolo[1,5-*a*]pyrimidine-3-carbonitrile (**6e**)

A yellow powder, 88% yield, mp 278–280 °C. IR (KBr) *ν*_max_/cm^−1^ 3110 (NH), 2235 (C≡N), 1600 (C=N). ^1^H NMR *δ* 6.96 (t, *J =* 8.0 Hz, 1H, H4 of PhNH), 7.31 (t, *J =* 7.5 Hz, 2H, H3 and H3′ of PhNH), 7.40 (d, *J =* 5.5 Hz, 1H, H3 of pyrimidine), 7.64 (d, *J =* 10.8 Hz, 2H, H2 and H2′ of PhNH), 7.73 (d, *J =* 8.0 Hz, 2H, H3 and H3′ of Ar), 8.18 (d, *J =* 9.0 Hz, 2H, H2 and H2′ of Ar), 8.68 (d, *J =* 6.5 Hz, 1H, pyrimidine H2), 9.61 (s, 1H, NH). ^13^C NMR *δ* 68.44 (C4 of pyrazole), 109.85, 113.95, 118.52 (2C), 122.03, 129.11 (3C), 129.24 (2C), 131.87 (2C), 136.85, 140.90, 145.32, 152.13, 152.81, 156.45. MS *m*/*z* (*%*) 345.93 (M^+^ + 1, 7.32), 345.15 (M^+^, 20.02). Anal. Calcd. for C_19_H_12_N_5_ (345.79): C, 66.00; H, 3.50; N, 20.25; found: C, 65.78; H, 3.72; N, 20.51.

##### 7-(4-Bromophenyl)-2-(anilinyl)pyrazolo[1,5-*a*]pyrimidine-3-carbonitrile (**6f**)

A yellow powder, 80% yield, mp 280–283 °C. IR (KBr) *ν*_max_/cm^−1^ 3320 (Aromatic CH), 2225 (C≡N), 1600 (C=N). ^1^H NMR *δ* 6.96 (t, *J =* 6.5 Hz, 1H, H4 of PhNH), 7.31 (t, *J =* 9.5 Hz, 2H, H3 and H3′ of PhNH), 7.39 (d, *J =* 5.0 Hz, 1H, H3 of pyrimidine), 7.64 (d, *J =* 7.0 Hz, 2H, H2 and H2′ of PhNH), 7.86 (d, *J =* 9.0 Hz, 2H, H3 and H3′ of Ar), 8.09 (d, *J =* 13.0 Hz, 2H, H2 and H2′ of Ar), 8.68 (d, *J =* 6.5 Hz, 1H, pyrimidine H2), 9.61 (s, 1H, NH). ^13^C NMR *δ* 68.35 (C4 of pyrazole), 109.78, 113.93, 118.54 (2C), 122.02, 125.77, 129.27, 129.31 (2C), 131.92 (2C), 132.02 (2C), 140.72, 145.41, 152.22, 152.84, 156.45. MS *m*/*z* (*%*) 392.33 (M^+^ + 2, 13.36), 391.33 (M^+^ + 1, 55.75), 390.37 (M^+^, 32.47). Anal. Calcd. for C_19_H_12_N_5_ (390.24): C, 58.48; H, 3.10; N, 17.95; found: C, 58.72; H, 3.29; N, 18.17.

##### 7-(4-Nitrophenyl)-2-(anilinyl)pyrazolo[1,5-*a*]pyrimidine-3-carbonitrile (**6g**)

An orange powder, 98% yield, mp over 300 °C. IR (KBr) *ν*_max_/cm^−1^ 3375 (NH), 2225 (C≡N), 1600 (C=N), 1350–1360 (NO_2_). ^1^H NMR *δ* 6.95 (t, *J =* 10.5 Hz, 1H, H4 of PhNH), 7.31 (t, *J =* 7.5 Hz, 2H, H3 and H3′ of PhNH), 7.49 (d, *J =* 4.5 Hz, 1H, H3 of pyrimidine), 7.63 (d, *J =* 8.0 Hz, 2H, H2 and H2′ of PhNH), 8.39 (d, *J =* 9.5 Hz, 2H, H2 and H2′ of Ar), 8.50 (d, *J =* 9.0 Hz, 2H, H3 and H3′ of Ar), 8.75 (d, *J =* 5.5 Hz, 1H, H2 of pyrimidine), 9.65 (s, 1H, NH). ^13^C NMR *δ* 77.43 (C4 of pyrazole), 82.73, 84.20, 91.41, 94.65, 123.80 (2C), 129.39 (2C), 131.45 (2C), 163.25 (2C), 172.08, 173.55, 178.56 (2C), 198.28 (2C). MS *m*/*z* (*%*) 357.34 (M^+^ + 1, 56.41), 356.36 (M^+^, 100). Anal. Calcd. for C_19_H_12_N_6_O_2_ (356.34): C, 64.04; H, 3.39; N, 23.58; found: C, 63.91; H, 3.60; N, 23.34.

##### 7-(Naphthalen-2-yl)-2-(anilinyl)pyrazolo[1,5-*a*]pyrimidine-3-carbonitrile (**6h**)

A yellow color, 80% yield, mp 271–272 °C. IR (KBr) *ν*_max_/cm^−1^ 3110 (NH), 2225 (C≡N), 1620 (C=N); ^1^H NMR *δ* 6.96 (t, *J =* 10.0 Hz, 1H, H4 of PhNH), 7.29 (t, *J =* 9.0 Hz, 2H, H3 and H3′ of PhNH), 7.54 (d, *J =* 8.5 Hz, 1H, H3 of pyrimidine), 7.68–7.74 (m, 4H, H2 and H2′ of PhNH and 2H of Ar), 8.09 (t, *J =* 7.5 Hz, 2H of Ar), 8.15–8.22 (m, 2H of Ar), 8.72 (d, *J =* 5.0 Hz, 1H, H2 of pyrimidine), 8.92 (s, 1H of Ar), 9.65 (s, 1H, NH). ^13^C NMR *δ* 110.00, 114.04, 118.58 (2C), 122.04, 126.13, 127.51, 127.64, 128.21, 128.37, 128.81, 129.13 (3C), 129.33, 130.99, 132.62, 134.46, 141.00, 146.25, 152.42, 152.77, 156.47. MS *m*/*z* (*%*) 362.38 (M^+^ + 1, 27.72), 361.37 (M^+^, 100). Anal. Calcd. for C_23_H_15_N_5_ (361.40): C, 76.44; H, 4.18; N, 19.38; found: C, 76.28; H, 4.31; N, 19.56.

##### 7-(Furan-2-yl)-2-(anilinyl)pyrazolo[1,5-*a*]pyrimidine-3-carbonitrile (**6i**)

A light-brown color, 85% yield, mp 250–252 °C. IR (KBr) *ν*_max_/cm^−1^ 3320 (NH), 2215 (C≡N), 1600 (C=N); ^1^H NMR *δ* 6.95–6.97 (m, 1H, H4 of furan), 7.03 (t, *J =* 8.5 Hz, 1H, H4 of PhNH), 7.39 (t, *J =* 8.0 Hz, 2H, H3 and H3′ of PhNH), 7.49 (d, *J =* 9.0 Hz, 1H, H3 of pyrimidine), 7.71 (d, *J =* 13.0 Hz, 2H, H2 and H2′ of PhNH), 7.99 (d, *J =* 6.5 Hz, 1H, H3 of furan), 8.21 (s, 1H, H5 of furan), 8.62 (d, *J =* 6.5 Hz, 1H, pyrimidine H2), 9.67 (s, 1H, NH). ^13^C NMR *δ* 68.06 (C4 of pyrazole), 104.54, 113.91, 114.14, 118.86 (2C), 120.35, 122.47, 129.34 (3C), 135.38, 140.69, 142.90, 148.42, 151.83, 151.89, 156.94. MS *m*/*z* (*%*) 302.61 (M^+^ + 1, 19.68), 301.74 (M^+^, 17.78). Anal. Calcd. for C_17_H_11_N_5_O (301.30): C, 67.77; H, 3.68; N, 23.24; found: C, 67.94; H, 3.85; N, 23.08.

##### 2-(Anilinyl)-7-(thiophen-2-yl)pyrazolo[1,5-*a*]pyrimidine-3-carbonitrile (**6j**)

A yellow powder, 88% yield, mp 265–266 °C. IR (KBr) *ν*_max_/cm^−1^ 3310 (NH), 2220 (C≡N), 1600 (C=N). ^1^H NMR *δ* 7.03 (t, *J =* 10.0 Hz, 1H, H4 of PhNH), 7.38–7.44 (m, 3H, H3 and H3′ of PhNH and H3 of pyrimidine), 7.86–7.90 (m, 3H, H2 and H2′ of PhNH and H4 of thiophene), 8.23 (d, *J =* 8.5 Hz, 1H, H5 of thiophene), 8.52 (d, *J =* 4.5 Hz, 1H, H3 of thiophene), 8.60 (d, *J =* 7.0 Hz, 1H, pyrimidine H2), 9.71 (s, 1H, NH). ^13^C NMR *δ* 67.88 (C4 of pyrazole), 105.89, 114.14, 118.62 (2C), 122.28, 128.53 (2C), 129.33 (2C), 133.32, 136.30, 139.96, 140.59, 151.72, 152.01, 156.25. MS *m*/*z* (*%*) 317.47 (M^+^, 13.98). Anal. Calcd. for C_17_H_11_N_5_S (317.37): C, 64.34; H, 3.49; N, 22.07; found: C, 64.58; H, 3.62; N, 22.31.

#### 3.1.5. Synthesis of Ethyl 2-(Anilinyl)-7-(aryl)pyrazolo[1,5-*a*]pyrimidine-3-carboxylate **6k**–**t**

A 25 mL mixture of acetic acid containing 10 mmol of the corresponding enaminone (**5a**–**j**) and 10 mmol (2.46 g) of ethyl 2-(anilinyl)-7-(aryl)pyrazolo[1,5-*a*]pyrimidine-3-carboxylate (**3b**) was heated for 3 h at reflux and then allowed to cool. The resultant was isolated by filtration, rinsed with ethanol, desiccated, and then purified by crystallization using a dimethyl formamide–water mixture to yield the corresponding products **6k**–**t**.

##### Ethyl 7-Phenyl-2-(anilinyl)pyrazolo[1,5-*a*]pyrimidine-3-carboxylate (**6k**)

A yellow powder, 79% yield, mp 155–156 °C. IR (KBr) *ν*_max_/cm^−1^ 3330 (NH), 1660 (C=O), 1600 (C=N). ^1^H NMR *δ* 1.37 (t, *J =* 7.0 Hz, 3H, CH_3_), 4.38 (q, *J =* 7.0 Hz, 2H, CH_2_), 6.96 (t, *J =* 7.0 Hz, 1H, H4 of PhNH), 7.29 (t, *J =* 8.0 Hz, 2H, H3 and H3′ of PhNH), 7.33 (d, *J =* 5.0 Hz, 1H, H3 of pyrimidine), 7.65–7.66 (m, 5H, H2 and H2′ of PhNH and H3, H3′ and H4 of Ph), 8.15–8.16 (m, 2H, H2 and H2′ of Ph), 8.70 (d, *J =* 4.5 Hz, 1H, H2 of pyrimidine), 9.03 (s, 1H, NH). ^13^C NMR *δ* 14.99 (-CH_3_), 60.18 (-CH_2_), 86.08 (C4 of pyrazole), 109.49, 117.97 (2C), 121.82, 128.92 (3C), 129.41 (2C), 130.00 (2C), 130.69, 131.87, 140.33, 145.99, 152.63, 157.09, 164.94. MS *m*/*z* (*%*) 359.67 (M^+^ + 1, 38.11), 358.47 (M^+^, 52.60). Anal. Calcd. for C_21_H_18_N_4_O_2_ (358.39): C, 77.38; H, 5.06; N, 15.63; found: C, 77.60; H, 5.32; N, 15.89.

##### Ethyl 2-(Anilinyl)-7-(p-tolyl)pyrazolo[1,5-*a*]pyrimidine-3-carboxylate (**6l**)

A yellow powder, 89% yield, mp 198–199 °C. IR (KBr) *ν*_max_/cm^−1^ 3325 (NH), 1665 (C=O), 1600 (C=N). ^1^H NMR *δ* 1.37 (t, *J =* 10.5 Hz, 3H, CH_3_), 2.44 (s, 3H, CH_3_ of Ar), 4.37 (q, *J =* 11.0 Hz, 2H, CH_2_), 6.96 (t, *J =* 8.5 Hz, 1H, H4 of PhNH), 7.30–7.32 (m, 3H, H3 and H3′ of PhNH and H3 of pyrimidine), 7.42–7.48 (m, 2H, H3 and H3′ of Ar), 7.65 (d, *J =* 9.5 Hz, 2H, H2 and H2′ of PhNH), 8.07–8.13 (m, 2H, H2 and H2′ of Ar), 8.67 (d, *J =* 15.0 Hz, 1H, pyrimidine H2), 9.02 (s, 1H, NH). ^13^C NMR *δ* 48.43 (-CH_3_), 59.76 (-CH_2_), 72.42 (C4 of pyrazole), 83.91, 88.03, 89.35, 98.18, 105.99, 109.81, 120.56 (2C), 138.08, 151.18, 160.74 (3C), 163.84, 186.06 (2C), 203.29 (3C). MS *m*/*z* (*%*) 373.66 (M^+^ + 1, 8.07), 372.93 (M^+^, 13.34); Anal. Calcd. for C_22_H_20_N_4_O_2_ (372.42): C, 70.95; H, 5.41; N, 15.04; found: C, 71.22; H, 5.63; N, 15.28.

##### Ethyl 7-(4-Methoxyphenyl)-2-(anilinyl)pyrazolo[1,5-*a*]pyrimidine-3-carboxylate (**6m**)

A yellow powder, 70% yield, mp 141–142 °C. IR (KBr) *ν*_max_/cm^−1^ 3325 (NH), 1670 (C=O), 1590 (C=N). ^1^H NMR *δ* 1.36 (t, *J =* 7.0 Hz, 3H, CH_3_), 3.89 (s, 3H, OCH_3_), 4.36 (q, *J =* 7.0 Hz, 2H, CH_2_), 6.96 (t, *J =* 7.0 Hz, 1H, H4 of PhNH), 7.15 (d, *J =* 8.5 Hz, 2H, H3 and H3′ of Ar), 7.26 (d, *J =* 4.5 Hz, 1H, H3 of pyrimidine), 7.32 (t, *J =* 7.5 Hz, 2H, H3 and H3′ of PhNH), 7.65 (d, *J =* 7.5 Hz, 2H, H2 and H2′ of PhNH), 8.17 (d, *J =* 8.5 Hz, 2H, H2 and H2′ of Ar), 8.61 (d, *J =* 4.5 Hz, 1H, pyrimidine H2), 9.02 (s, 1H, NH). ^13^C NMR *δ* 14.85 (-CH_3_), 55.85 (OCH_3_), 60.19 (-CH_2_), 85.71 (C4 of pyrazole), 108.44, 114.17 (2C), 117.77 (2C), 121.73, 122.34, 129.38 (2C), 131.74 (2C), 140.14, 145.54, 148.58, 152.07, 156.83, 162.01, 164.92. MS *m*/*z* (*%*) 389.41 (M^+^ + 1, 12.98), 388.39 (M^+^, 51.57). Anal. Calcd. for C_22_H_20_N_4_O_3_ (388.42): C, 68.03; H, 5.19; N, 14.42; found: C, 68.29; H, 5.28; N, 14.67.

##### Ethyl 7-(3,4-Dimethoxyphenyl)-2-(anilinyl)pyrazolo[1,5-*a*]pyrimidine-3-carboxylate (**6n**)

A yellow powder, 84% yield, mp 142–143 °C. IR (KBr) *ν*_max_/cm^−1^ 3300 (NH), 1665 (C=O), 1600 (C=N); ^1^H NMR *δ* 1.39 (t, *J =* 7.0 Hz, 3H, CH_3_), 3.88 (s, 3H, OCH_3_), 3.91 (s, 3H, OCH_3_), 4.40 (q, *J =* 7.0 Hz, 2H, CH_2_), 6.99 (t, *J =* 7.5 Hz, 1H, H4 of PhNH), 7.23 (d, *J =* 8.5 Hz, 1H, H5 of Ar), 7.33 (t, *J =* 7.5 Hz, 2H, H3 and H3′ of PhNH), 7.39 (d, *J =* 5.0 Hz, 1H, H3 of pyrimidine), 7.73–7.77 (m, 3H, H2 and H2′ of PhNH and H6 of Ar), 7.98 (s, 1H, H2 of Ar), 8.68 (d, *J =* 5.0 Hz, 1H, pyrimidine H2), 9.11 (s, 1H, NH). ^13^C NMR *δ* 14.87 (-CH_3_), 55.93 (OCH_3_), 56.11 (OCH_3_), 60.38 (-CH_2_), 85.76 (C4 of pyrazole), 109.00, 111.84, 112.92, 117.91 (2C), 122.09, 122.57, 123.88, 129.45 (2C), 140.17, 145.99, 148.39, 148.72, 151.85, 152.32, 157.00, 165.02. MS *m*/*z* (*%*) 418.97 (M^+^ + 1, 10.90), 416.40 (M^+^, 13.82). Anal. Calcd. for C_23_H_22_N_4_O_4_ (418.45): C, 66.02; H, 5.30; N, 13.39; found: C, 65.79; H, 5.38; N, 13.61.

##### Ethyl 7-(4-Chlorophenyl)-2-(anilinyl)pyrazolo[1,5-*a*]pyrimidine-3-carboxylate (**6o**)

Yellow powder, 92% yield, mp 197–198 °C. IR (KBr) *ν*_max_/cm^−1^ 3310 (NH), 1660 (C=O), 1600 (C=N). ^1^H NMR *δ* 1.38 (t, *J =* 7.0 Hz, 3H, CH_3_), 4.40 (q, *J =* 7.0 Hz, 2H, CH_2_), 6.98 (t, *J =0* 7.0 Hz, 1H, H4 of PhNH), 7.34 (t, *J =* 15.0 Hz, 2H, H3 and H3′ of PhNH), 7.40 (d, *J =* 4.5 Hz, 1H, H3 of pyrimidine), 7.66 (d, *J =* 8.5 Hz, 2H, H2 and H2′ of PhNH), 7.75 (d, *J =* 8.5 Hz, 2H, H3 and H3′ of Ar), 8.21 (d, *J =* 8.5 Hz, 2H, H2 and H2′ of Ar), 8.74 (d, *J =* 4.5 Hz, 1H, pyrimidine H2), 9.05 (s, 1H, NH). ^13^C NMR *δ* 15.16 (-CH_3_), 62.86 (-CH_2_), 74.93 (C4 of pyrazole), 115.50, 117.81 (2C), 122.42, 129.09 (3C), 129.59 (3C), 131.75 (2C), 134.30, 140.90, 146.30, 147.00, 174.43 (2C). MS *m*/*z* (*%*) 393.38 (M^+^ + 1, 21.50), 392.52 (M^+^, 100), 392.95 (M^+^, 28.43). Anal. Calcd. for C_21_H_17_N_4_O_2_ (392.84): C, 64.21; H, 4.36; N, 14.26; found: C, 64.47; H, 4.52; N, 14.50.

##### Ethyl 7-(4-Bromophenyl)-2-(anilinyl)pyrazolo[1,5-*a*]pyrimidine-3-carboxylate (**6p**)

A yellow powder, 93% yield, mp 201–202 °C. IR (KBr) *ν*_max_/cm^−1^ 3300 (NH), 1670 (C=O), 1600 (C=N); ^1^H NMR *δ* 1.38 (t, *J =* 7.0 Hz, 3H, CH_3_), 4.39 (q, *J =* 7.0 Hz, 2H, CH_2_), 6.97 (t, *J =* 7.0 Hz, 1H, H4 of PhNH), 7.33 (t, *J =* 8.0 Hz, 2H, H3 and H3′ of PhNH), 7.36 (d, *J =* 5.0 Hz, 1H, H3 of pyrimidine), 7.63 (d, *J =* 8.0 Hz, 2H, H2 and H2′ of PhNH), 7.86 (d, *J =* 8.5 Hz, 2H, H3 and H3′ of Ar), 8.11 (d, *J =* 9.0 Hz, 2H, H2 and H2′ of Ar), 8.71 (d, *J =* 5.0 Hz, 1H, pyrimidine H2), 9.03 (s, 1H, NH). ^13^C NMR *δ* 14.99 (-CH_3_), 60.21 (-CH_2_), 86.16 (C4 of pyrazole), 109.46, 118.03 (2C), 121.90, 125.56, 129.51 (2C), 129.85, 131.99 (2C), 132.03 (2C), 140.25, 144.88, 148.75, 152.64, 157.10, 164.90. MS *m*/*z* (*%*) 439.91 (M^+^ + 2, 6.85), 438.61 (M^+^ + 1, 6.82), 437.44 (M^+^, 6.84). Anal. Calcd. for C_21_H_17_N_4_O_2_ (437.29): C, 57.68; H, 3.92; N, 12.81; found: C, 57.89; H, 4.15; N, 13.08.

##### Ethyl 7-(4-Nitrophenyl)-2-(anilinyl)pyrazolo[1,5-*a*]pyrimidine-3-carboxylate (**6q**)

An orange powder, 94% yield, mp 223–224 °C. IR (KBr) *ν*_max_/cm^−1^ 3350 (NH), 1675 (C=O), 1600 (C=N), 1350 (NO_2_). ^1^H NMR *δ* 1.37 (t, *J =* 7.0 Hz, 3H, CH_3_), 4.39 (q, *J =* 7.0 Hz, 2H, CH_2_), 6.96 (t, *J =* 7.0 Hz, 1H, H4 of PhNH), 7.31 (t, *J =* 7.5 Hz, 2H, H3 and H3′ of PhNH), 7.42 (d, *J =* 4.5 Hz, 1H, H3 of pyrimidine), 7.61 (d, *J =* 8.0 Hz, 2H, H2 and H2′ of PhNH), 8.39 (d, *J =* 9.0 Hz, 2H, H2 and H2′ of Ar), 8.45 (d, *J =* 9.0 Hz, 2H, H3 and H3′ of Ar), 8.76 (d, *J =* 4.5 Hz, 1H, H2 of pyrimidine), 9.02 (s, 1H, NH). ^13^C NMR *δ* 14.92 (-CH_3_), 60.34 (-CH_2_), 86.30 (C4 of pyrazole), 110.10, 118.02 (2C), 122.00, 123.81 (2C), 129.49 (2C), 131.47 (2C), 136.66, 140.03, 143.61, 148.44, 149.13, 152.69, 157.07, 164.82. MS *m*/*z* (*%*) 403.03 (M^+^, 47.06). Anal. Calcd. for C_21_H_17_N_5_O_4_ (403.39): C, 62.53; H, 4.25; N, 17.36; found: C, 62.79; H, 4.53; N, 17.62.

##### Ethyl 7-(Naphthalen-2-yl)-2-(anilinyl)pyrazolo[1,5-*a*]pyrimidine-3-carboxylate (**6r**)

A pale-yellow color, 68% yield, mp 160 °C. IR (KBr) *ν*_max_/cm^−1^ 3325 (NH), 1665 (C=O), 1600 (C=N); ^1^H NMR *δ* 1.37 (t, *J =* 7.0 Hz, 3H, CH_3_), 4.37 (q, *J =* 7.0 Hz, 2H, CH_2_), 6.93 (t, *J =* 7.5 Hz, 1H, H4 of PhNH), 7.25 (t, *J =* 7.5 Hz, 2H, H3 and H3′ of PhNH), 7.43 (d, *J =* 5.0 Hz, 1H, H3 of pyrimidine), 7.62–7.68 (m, 4H, H2 and H2′ of PhNH and 2H of Ar), 8.02 (d, *J =* 8.0 Hz, 2H of Ar), 8.09 (d, *J =* 8.5 Hz, 2H of Ar), 8.15 (d, *J =* 8.5 Hz, 2H of Ar), 8.70 (d, *J =* 4.5 Hz, 1H, H2 of pyrimidine), 8.86 (s, 1H of Ar), 9.01 (s, 1H, NH). ^13^C NMR *δ* 14.25 (-CH_3_), 59.59 (-CH_2_), 85.37 (C4 of pyrazole), 108.82, 117.30, 121.20, 125.42, 126.84, 127.07, 127.52, 127.70, 128.02, 128.53, 128.64, 130.21, 131.90, 133.64, 139.55, 140.19, 144.91, 147.96, 151.74, 156.32, 164.23, 172.69. MS *m*/*z* (*%*) 409.48 (M^+^ + 1, 5.99), 408.47 (M^+^, 17.79). Anal. Calcd. for C_25_H_20_N_4_O_2_ (408.45): C, 73.51; H, 4.94; N, 13.72; found: C, 73.38; H, 5.12; N, 14.01.

##### Ethyl 7-(Furan-2-yl)-2-(anilinyl)pyrazolo[1,5-*a*]pyrimidine-3-carboxylate (**6s**)

A yellow powder, 80% yield, mp 145–146 °C. IR (KBr) *ν*_max_/cm^−1^ 3310 (NH), 1670 (C=O), 1610 (C=N). ^1^H NMR *δ* 1.37 (t, *J =* 7.0 Hz, 3H, CH_3_), 4.38 (q, *J =* 7.0 Hz, 2H, CH_2_), 6.99 (s, 1H, H4 of furan), 7.06 (t, *J =* 7.0 Hz, 1H, H4 of PhNH), 7.43 (t, *J =* 7.5 Hz, 2H, H3 and H3′ of PhNH), 7.51 (d, *J =* 5.0 Hz, 1H, H3 of pyrimidine), 7.74 (d, *J =* 8.0 Hz, 2H, H2 and H2′ of PhNH), 8.07 (d, *J =* 3.0 Hz, 1H, H3 of furan), 8.22 (s, 1H, H5 of furan), 8.67 (d, *J =* 5.0 Hz, 1H, pyrimidine H2), 9.11 (s, 1H, NH). ^13^C NMR *δ* 14.91 (-CH_3_), 60.32 (-CH_2_), 85.61 (C4 of pyrazole), 104.31, 114.11, 118.33, 120.11, 122.34, 129.62 (2C), 132.30, 135.09, 140.03, 143.14, 148.14, 151.65, 157.46, 164.95, 172.83. MS *m*/*z* (*%*) 349.40 (M^+^ + 1, 16.36), 348.52 (M^+^, 33.07); Anal. Calcd. for C_19_H_16_N_4_O_3_ (348.36): C, 65.51; H, 4.63; N, 16.08; found: C, 65.75; H, 4.80; N, 16.23.

##### Ethyl 2-(Anilinyl)-7-(thiophen-2-yl)pyrazolo[1,5-*a*]pyrimidine-3-carboxylate (**6t**)

A yellow powder, 85% yield, mp 172–173 °C. IR (KBr) *ν*_max_/cm^−1^ 3320 (NH), 1700 (C=O), 1650 (C=N); ^1^H NMR *δ* 1.37 (t, *J =* 7.0 Hz, 3H, CH_3_), 4.38 (q, *J =* 7.0 Hz, 2H, CH_2_), 7.05 (t, *J =* 7.0 Hz, 1H, H4 of PhNH), 7.41–7.44 (m, 3H, H3 and H3′ of PhNH and H3 of pyrimidine), 7.84–7.88 (m, 3H, H2 and H2′ of PhNH and H4 of thiophene), 8.22 (d, *J =* 5.0 Hz, 1H, H5 of thiophene), 8.50 (d, *J =* 3.0 Hz, 1H, H3 of thiophene), 8.63 (d, *J =* 4.5 Hz, 1H, pyrimidine H2), 9.19 (s, 1H, NH). ^13^C NMR *δ* 14.91 (-CH_3_), 60.29 (-CH_2_), 85.72 (C4 of pyrazole), 105.51, 118.11 (2C), 122.12, 128.31, 129.57, 129.62, 132.92, 135.97, 139.68, 139.85, 148.41, 151.40, 156.74, 165.05, 172.77. MS *m*/*z* (*%*) 365.39 (M^+^ + 1, 16.24), 364.31 (M^+^, 100). Anal. Calcd. for C_19_H_16_N_4_O_2_S (364.42): C, 62.62; H, 4.43; N, 15.37; found: C, 62.90; H, 4.67; N, 15.58.

##### 3.1.6. 2-Aryl-3-(dimethylamino)acrylonitrile **10a**–**e** Preparation

3-Oxo-3-(aryl)propanenitrile **9a**–**e** (20 mmol) was dissolved in xylene (50 mL) and (2.38 g, 20 mmol) DMF-DMA was added, and subjected to reflux for approximately 6–10 h. The solvent was evaporated under vacuum and the residue was triturated with 5 mL of diethyl ether, filtered, and washed with cold petroleum ether, yielding products **10a**–**e**, respectively. The physicochemical characteristics of **10a**–**e** were in agreement with those documented in the literature [32,33,34].

#### 3.1.7. 2-(Anilinyl)-7-(aryl)pyrazolo[1,5-*a*]pyrimidine-3,6-dicarbonitrile **11a**–**c** Preparation

A combination of (10 mmol) suitable enaminone **10a**–**e** with (1.99 g, 10 mmol) 5-amino-3-(anilinyl)-1H-pyrazole-4-carbonitrile **3a** were mixed with (25 mL) acetic acid and refluxed for 3 h followed by cooling. The resultant isolated by filtration, rinsed with ethanol, desiccated, and then purified by crystallization using a dimethyl formamide–water mixture to provide the equivalent 2-(anilinyl)-7-(aryl)pyrazolo[1,5-*a*]pyrimidine-3,6-dicarbonitrile **11a**–**c**, respectively.

##### 7-Phenyl-2-(anilinyl)pyrazolo[1,5-*a*]pyrimidine-3,6-dicarbonitrile (**11a**)

A yellow powder, 95% yield, mp 280–281 °C. IR (KBr) *ν*_max_/cm^−1^ 3300 (NH), 2225 (2C≡N), 1610 (C=N). ^1^H NMR *δ* 6.97 (t, *J =* 7.5 Hz, 1H, H4 of PhNH), 7.25 (t, *J =* 8.5 Hz, 2H, H3 and H3′ of PhNH), 7.57 (d, *J =* 9.0 Hz, 2H, H2 and H2′ of PhNH), 7.69–7.77 (m, 3H, H3, H3′ and H4 of Ph), 7.96–7.98 (m, 2H, H2 and H2′ of Ph), 9.01 (s, 1H, pyrimidine H2), 9.87 (s, 1H, NH). ^13^C NMR *δ* 71.34 (pyrazole C4), 95.99 (pyrimidine C3), 118.96 (3C), 122.98, 127.48, 128.94, 129.26 (3C), 130.40 (3C), 133.00, 139.97, 150.88, 152.30, 154.05, 157.46. MS *m*/*z* (*%*) 337.90 (M^+^ + 1, 42.20), 336.97 (M^+^, 48.24). Anal. Calcd. for C_20_H_12_N_6_ (336.35): C, 71.42; H, 3.60; N, 24.99; found: C, 71.69; H, 3.54; N, 24.78.

##### 7-(4-Bromophenyl)-2-(anilinyl)pyrazolo[1,5-*a*]pyrimidine-3,6-dicarbonitrile (**11b**)

A yellow powder, 90% yield, mp 268–269 °C. IR (KBr) *ν*_max_/cm^−1^ 3310 (NH), 2220 (2C≡N), 1610 (C=N). ^1^H NMR *δ* 6.99 (t, *J =* 7.5 Hz, 1H, H4 of PhNH), 7.29 (t, *J =* 7.5 Hz, 2H, H3 and H3′ of PhNH), 7.56 (d, *J =* 7.5 Hz, 2H, H2 and H2′ of PhNH), 7.91–7.98 (m, 4H, H2, H2′, H3 and H3′ of Ar), 9.05 (s, 1H, pyrimidine H2), 9.89 (s, 1H, NH). ^13^C NMR *δ* 71.50 (pyrazole C4), 96.00 (pyrimidine C3), 113.20, 115.70, 118.99 (2C), 122.89, 126.73, 126.81, 129.28 (2C), 132.08 (2C), 132.53 (2C), 140.07, 149.86, 152.30, 154.02, 157.50. MS *m*/*z* (*%*) 417.21 (M^+^ + 2, 65.69), 416.27 (M^+^ + 1, 92.67), 415.28 (M^+^, 89.99). Anal. Calcd. for C_20_H_11_N_6_ (415.25): C, 57.85; H, 2.67; N, 20.24; found: C, 58.09; H, 2.83; N, 20.51.

##### 7-(Furan-2-yl)-2-(anilinyl)pyrazolo[1,5-*a*]pyrimidine-3,6-dicarbonitrile (**11c**)

A black powder, 69% yield, mp over 300 °C. IR (KBr) *ν*_max_/cm^−1^ 3320 (N), 2215 (2C≡N), 1610 (C=N). ^1^H NMR *δ* 7.07–7.10 (m, 2H, H4 of furan and H4 of PhNH), 7.42 (t, *J =* 8.0 Hz, 2H, H3 and H3′ of PhNH), 7.70 (d, *J =* 8.0 Hz, 2H, H2 and H2′ of PhNH), 8.27 (d, *J =* 4.5 Hz, 1H, H3 of furan), 8.43 (s, 1H, H5 of furan), 8.91 (s, 1H, pyrimidine H2), 9.95 (s, 1H, NH). ^13^C NMR *δ* 70.99 (pyrazole C4), 90.39 (pyrimidine C3), 104.37, 112.89, 114.85, 116.34, 119.38, 123.26, 124.95, 129.45, 137.19, 139.99, 141.49, 149.88, 152.34, 154.61, 156.77, 157.58; MS *m*/*z* (*%*) 326.60 (M^+^, 46.13). Anal. Calcd. for C_18_H_10_N_6_O (326.31): C, 66.25; H, 3.09; N, 25.75; found: C, 66.17; H, 3.26; N, 25.93.

#### 3.1.8. Synthesis of Ethyl 6-Cyano-2-(anilinyl)-7-(aryl)pyrazolo[1,5-*a*]pyrimidine-3-carboxylate **11d**–**g**

The procedure was identical to the preparation of compounds **11a**–**c**, where (10 mmol) suitable enaminone **10a**–**e** and (**3b**) (2.46 g, 10 mmol) 5-amino-3-(anilinyl)-1H-pyrazole-4-carbonitrile instead of (**3a**) were mixed in (25 mL) acetic acid under reflux for 3 h followed by cool. The resultant was isolated by filtration, rinsed with ethanol, desiccated, and then, subjected to recrystallization from a dimethyl formamide–water mixture providing the final corresponding ethyl 6-cyano-2-(anilinyl)-7-(aryl)pyrazolo[1,5-*a*]pyrimidine-3-carboxylate **11d**–**g**, respectively.

##### Ethyl 6-Cyano-7-phenyl-2-(anilinyl)pyrazolo[1,5-*a*]pyrimidine-3-carboxylate (**11d**)

A yellow powder, 65% yield, mp 158–159 °C. IR (KBr) *ν*_max_/cm^−1^ 3290 (NH), 2210 (2C≡N), 1600 (C=N). ^1^H NMR *δ* 1.38 (t, *J =* 7.0 Hz, 3H, CH_3_), 4.44 (q, *J =* 7.0 Hz, 2H, CH_2_), 6.97 (t, *J =* 7.5 Hz, 1H, H4 of PhNH), 7.26 (t, *J =* 8.0 Hz, 2H, H3 and H3′ of PhNH), 7.59 (d, *J =* 8.0 Hz, 2H, H2 and H2′ of PhNH), 7.71–7.77 (m, 3H, H3, H3′ and H4 of Ph), 7.98 (d, *J =* 6.0 Hz, 2H, H2 and H2′ of Ph), 9.04 (s, 1H, H2 of pyrimidine), 9.06 (s, 1H, NH). ^13^C NMR *δ* 14.84 (-CH_3_), 60.88 (-CH_2_), 88.79 (pyrazole C4), 95.51 (pyrimidine C3), 118.35 (2C), 122.59, 127.95, 128.88, 129.43 (3C), 130.51 (2C), 132.82 (2C), 139.61, 148.42, 150.50, 153.84, 158.05, 164.36. MS *m*/*z* (*%*) 383.75 (M^+^, 14.78). Anal. Calcd. for C_22_H_17_N_5_O_2_ (383.40): C, 68.92; H, 4.47; N, 18.27; found: C, 69.14; H, 4.71; N, 18.09.

##### Ethyl 6-Cyano-7-(4-methoxyphenyl)-2-(anilinyl)pyrazolo[1,5-*a*]pyrimidine-3-carboxylate (**11e**)

A yellow powder, 80% yield, mp 208–209 °C. IR (KBr) *ν*_max_/cm^−1^ 3290 (NH), 2210 (2C≡N), 1670 (C=O), 1600 (C=N); ^1^H NMR *δ* 1.38 (t, *J =* 7.0 Hz, 3H, CH_3_), 3.94 (s, 3H, OCH_3_), 4.43 (q, *J =* 7.0 Hz, 2H, CH_2_), 6.99 (t, *J =* 7.0 Hz, 1H, H4 of PhNH), 7.27–7.33 (m, 4H, H3 and H3′ of PhNH and H3 and H3′ of Ar), 7.63 (d, *J =* 8.0 Hz, 2H, H2 and H2′ of PhNH), 8.01 (d, *J =* 9.0 Hz, 2H, H2 and H2′ of Ar), 9.01 (s, 1H, pyrimidine H2), 9.04 (s, 1H, NH). ^13^C NMR *δ* 14.28 (-CH_3_), 54.61 (-CH_2_), 56.53 (OCH_3_), 65.95 (C4 of pyrazole), 109.96 (3C), 122.77 (3C), 151.32 (2C), 162.95 (2C), 179.00 (2C), 186.36 (2C), 191.22 (2C), 208.14 (2C). MS *m*/*z* (*%*) 414.42 (M^+^ + 1, 9.12), 413.39 (M^+^, 33.16). Anal. Calcd. for C_23_H_19_N_5_O_3_ (413.43): C, 66.82; H, 4.63; N, 16.94; found: C, 66.98; H, 4.80; N, 17.12.

##### Ethyl 6-Cyano-7-(4-fluorophenyl)-2-(anilinyl)pyrazolo[1,5-*a*]pyrimidine-3-carboxylate (**11f**)

A yellow powder, 88% yield, mp 221–222 °C. IR (KBr) *ν*_max_/cm^−1^ 3310 (NH), 2220 (2C≡N), 1675 (C=O), 1600 (C=N). ^1^H NMR *δ* 1.38 (t, *J =* 7.0 Hz, 3H, CH_3_), 4.43 (q, *J =* 7.0 Hz, 2H, CH_2_), 6.99 (t, *J =* 7.0 Hz, 1H, H4 of PhNH), 7.30 (t, *J =* 8.0 Hz, 2H, H3 and H3′ of PhNH), 7.58–7.62 (m, 4H, H2 and H2′ of PhNH and H3 and H3′ of Ar), 8.07–8.11 (m, 2H, H2 and H2′ of Ar), 9.04 (s, 1H, pyrimidine H2), 9.06 (s, 1H, NH). ^13^C NMR *δ* 14.10 (-CH_3_), 44.60 (-CH_2_), 56.67, 59.18, 71.25, 78.02, 109.37, 110.84, 124.39 (3C), 125.42, 128.36, 132.48, 134.98, 163.25 (3C), 165.16 (2C), 196.22. MS *m*/*z* (*%*) 402.22 (M^+^ + 1, 5.43), 400.95 (M^+^, 4.30). Anal. Calcd. for C_22_H_16_N_5_O_2_ (401.39): C, 65.83; H, 4.02; N, 17.45; found: C, 65.71; H, 4.23; N, 17.63.

##### Ethyl 6-Cyano-7-(furan-2-yl)-2-(anilinyl)pyrazolo[1,5-*a*]pyrimidine-3-carboxylate (**11g**)

A dark-brown powder, 87% yield, mp 219–220 °C. IR (KBr) *ν*_max_/cm^−1^ 3300 (NH), 2210 (2C≡N), 1670 (C=O), 1600 (C=N). ^1^H NMR *δ* 1.38 (t, *J =* 7.0 Hz, 3H, CH_3_), 4.43 (q, *J =* 7.0 Hz, 2H, CH_2_), 7.12–7.13 (m, 2H, H4 of PhNH and H4 of furan), 7.46 (t, *J =* 8.0 Hz, 2H, H3 and H3′ of PhNH), 7.75 (d, *J =* 8.0 Hz, 2H, H2 and H2′ of PhNH), 8.33 (d, *J =* 3.5 Hz, 1H, H3 of furan), 8.43 (s, 1H, H5 of furan), 8.96 (s, 1H, pyrimidine H2), 9.15 (s, 1H, NH). ^13^C NMR *δ* 14.10 (-CH_3_), 45.19 (-CH_2_), 47.99, 66.83, 71.83, 99.21, 105.40, 112.00, 113.50, 129.83, 140.90, 142.90, 147.00, 152.60, 177.08 (3C), 194.45 (3C). MS *m*/*z* (*%*) 373.43 (M^+^, 40.93). Anal. Calcd. for C_20_H_15_N_5_O_3_ (373.36): C, 64.34; H, 4.05; N, 18.76; found: C, 64.58; H, 4.12; N, 18.98.

#### 3.1.9. 7-Amino-5-oxo-2-(anilinyl)-4,5-dihydropyrazolo[1,5-*a*]pyrimidine-3-carbonitrile (**12**) Preparation

A combination of ethyl cyanoacetate (0.113 g, 1 mmol) and (**3a**) (0.2 g, 1 mmol) was refluxed for 45 min and then cooled down. The resultant was isolated by filtration, rinsed with ethanol, then subjected to recrystallization from a dimethyl formamide–ethanol mixture providing the final product (**12**) as a brown-hued powder, 75% yield, mp over 300 °C. IR (KBr) *ν*_max_/cm^−1^ 3150–3400 (NH and NH_2_), 3100 (CH Aromatic), 2220 (C≡N), 1660 (C=O), 1600 (C=N). ^1^H NMR *δ* 4.0 (s, D_2_O exchangeable, 1H, NH pyrimidine), 5.27 (s, 1H, CH pyrimidine), 6.91 (t, *J =* 7.0 Hz, 1H, H4 of PhNH), 7.27 (t, *J =* 7.0 Hz, 2H, H3 and H3′ of PhNH), 7.53 (s, D_2_O exchangeable, 2H, NH_2_), 7.75 (d, *J =* 15.0 Hz, 2H, H2 and H2′ of PhNH), 9.07 (br. s, 1H and NH protons exchangeable with D_2_O). ^13^C NMR *δ* 57.70, 64.88 (C4 of pyrazole), 76.71, 118.06 (3C), 121.34, 129.22 (3C), 141.31, 149.78, 154.41. MS *m*/*z* (*%*) 267.63 (M^+^ + 1, 33), 266.49 (M^+^, 25.40). Anal. Calcd. for C_13_H_10_N_6_O (266.26): C, 58.64; H, 3.79; N, 31.56; found: C, 59.91; H, 3.95; N, 31.28.

### 3.2. Biological Evaluation

#### 3.2.1. In Vitro Anti-Proliferative Activity Toward 60 Cell Lines

Performed by US National Cancer Institute according to previously reported standard procedure [44,45,46] as follows: Cells were plated into 96-well microtiter plates with each well equipped with 100 µL of culture medium at densities between 5000 and 40,000 cells per well, based on the specific cell line’s doubling time. After seeding, incubation was carried out at 37 °C in 5% carbon dioxide, 95% oxygen environment with 100% relative humidity for one day to permit the cells to attach before proceeding to treatment with the tested compounds. To determine the cell count at drug exposure time of (Tz), two plates were per fixed cell line using trichloroacetic acid after the 24 h incubation period.

The studied compounds were initially dispersed in dimethyl sulfoxide at a concentration 400 times exceeding the intended final concentration and subsequently frozen until required. Before the application, an aliquot of the stock solution was warmed to room temperature and diluted in a complete medium containing 50 µg per mL of gentamicin to attain twice the intended concentration. Subsequently, 100 µL of the diluted compound was introduced to every well holding 100 µL of the medium to achieve the intended concentration. After the compound was added, the plates were incubated under the same conditions for a further 48 h at 37 °C.

For the adherent cells, the experiment was concluded via the introduction of 50 µL of cold 50% (*w*/*v*) trichloroacetic acid into each well, resulting in a last TCA concentration for 10%. Then, the cell plates were incubated at 4 °C for about one hour to fix the cells.

After the removal of the supernatant, the plates were washed with tap water five times before being dried using air. For the suspension cells, a similar process was followed, but 50 µL of 80% TCA was used, yielding a final TCA concentration of 16% for the cells fixed in the well’s bottom.

Following cell fixation, 100 µL of the 0.4% (*wight*/*volume*) solution of sulforhodamine B with acetic acid at a concentration of 1% was introduced to all individual wells, and then incubated at ambient temperature for 10 min. The plates were washed five times with 1% acetic acid to eliminate any residual dye, followed by air-drying. Next, 10 µM Tris base was added to disperse the bonded dye, and, using an automatic microplate reader, the absorbance was determined at 515 nm.

The percentage of growth inhibition measurement depended on the absorbance values at the baseline time point (T0), as well as during the control growth phase (C) and the test growth phase (Ti). When Ti was greater than or equal to Tz, the calculation formula used [(Ti − Tz)/(C − Tz)] × 100. If Ti was less than Tz, the formula used was (Ti − Tz)/Tz × 100.

#### 3.2.2. Kinase

##### CDK2 Inhibition Assay

The suppression of the CDK2/CyclinA2 enzyme by the tested compounds was measured using the CDK2 analysis kit (Cat. #79599) from BPS Bioscience, San Diego, CA, USA in accordance with the protocol designed by the manufacturer. Prior to the experiment, ATP, 10× CDK substrate peptide 1, and 5× kinase assay buffer 1 were brought to room temperature. A supreme solution was developed to dispense 25 µL per well by combining the following components: 13 µL of distilled water, 6 µL of 5× kinase assay buffer 1, 1 µL of ATP (500 µM), and 5 µL of 10× CDK substrate peptide 1. per reaction (N wells × proportions). Each well received 25 µL of this master solution.

Firstly, 5 µL of the inhibitor solution was introduced into the test inhibitor wells. Then, 5 µL of inhibitor-free buffer (Inhibitor buffer) was introduced into the “Positive Control” and “Blank” wells.

A 1× Kinase assay buffer 1 working solution had been made via combining 600 µL of 5× Kinase assay buffer 1 with 2400 µL of water, yielding a total of 3 mL, enough for 100 reactions. In the “Blank” wells, 20 µL of 1× Kinase assay buffer 1 was introduced.

The CDK2/CyclinA2 enzyme was maintained in ice and, upon initial thawing, it was briefly centrifuged to collect the entire contents. It was then adjusted to a concentration of approximately 5 ng/µL in 1× Kinase assay buffer 1 based on the assay requirements. The enzyme, without dilution, was aliquoted and kept at −80 °C for storage to minimize the freeze–thaw cycles, as the enzyme is sensitive to such conditions. The samples that had been previously warmed or diluted with the enzyme were not reused.

To start the reaction in the “Positive Control” and “Test Inhibitor” wells, 20 µL of the prepared enzyme solution was introduced, which was incubated at 30 °C for over 45 min. Following incubation, every well received 50 µL of the thawed Kinase-Glo Max reagent. The plate was sealed using aluminum foil and left to incubate for 15 min at ambient temperature. The measurement of the luminescence was performed via a Tecanspark Microplate reader (Männedorf, Switzerland) capable of luminescence detection. Blank values were deducted from all measurements.

The assay evaluated samples at four concentrations: 0.01, 0.1, 1, and 10 µM. All samples, blanks, and standards were tested in triplicate. Ribociclib was incorporated as a standard reference drug and positive control for the experiment [47].

##### TRKA Inhibition Assay

The suppression of the TrkA enzyme with the tested compounds was measured using the TrkA kinase analysis kit (Cat. #79548) from BPS Bioscience, San Diego, CA, USA, in compliance with the comprehensive directives given in the protocol of the manufacturer. The assay began with thawing ATP, 5× Kinase Assay Buffer 1, and the PTK Substrate Poly (Glu:Tyr 4:1) with a concentration of 10 mg/mL. To prepare the assay buffer, dithiothreitol was incorporated into the 5× Kinase Assay Buffer 1 to obtain the desired concentration of 10 µM.

A supreme solution has been developed to dispense 25 µL per well by combining the following components: 17 µL of water, 1 µL of ATP (500 µM), 1 µL of PTK Substrate Poly (Glu:Tyr 4:1) at 10 mg/mL, and 6 µL of 5× Kinase Assay Buffer 1. Each well received 25 µL of this master solution.

Firstly, 5 µL of the inhibitor solution were dispensed into the test inhibitor wells. Then, 5 µL of the Positive Control and Blank wells received an addition of buffer without inhibitors (Inhibitor buffer). The DMSO concentration in the inhibitor solution was kept below 10% to ensure that the final reaction concentration did not exceed 1%.

A working solution of 1× Kinase Assay Buffer 1 was produced through the process of combining 600 µL of 5× Kinase Assay Buffer 1 in addition to 2400 µL of water, resulting in a total volume of 3 mL, adequate for 100 reactions. In the “Blank” wells, 20 µL of the 1× Kinase Assay Buffer 1 was introduced.

The TrkA enzyme was maintained on ice and, and upon initial thawing was briefly centrifuged to collect the entire contents. It was then adjusted to a concentration of approximately 5 ng/µL in 1× Kinase Assay Buffer 1 based on the assay requirements. The enzyme, without dilution was aliquoted and kept at −80 °C for storage to minimize freeze–thaw cycles, as the enzyme is sensitivity to such conditions. The samples that were previously warmed or the diluted enzyme were not reused.

To start the reaction in the “Positive Control” and “Test Inhibitor” wells, 20 µL of the prepared enzyme solution was introduced, which had been incubated at 30 °C for a duration of 45 min. Following incubation, every well received 50 µL of the thawed Kinase-Glo Max reagent. The plate was sealed with aluminum foil and incubated at ambient temperature for 15 min to ensure proper reaction conditions. The measurement of luminescence was performed using a Tecanspark Microplate reader V1.0 capable of luminescence detection. The Blank values were deducted from all recorded readings to ensure accurate results.

The assay evaluated samples at four concentrations: 0.01, 0.1, 1, and 10 µM. All samples, blanks, and standards were tested in triplicate. Larotrectinib sulfate, a highly potent and selective ATP-competitive inhibitor of tropomyosin receptor kinases (TRKs), was used as the positive control (Catalog No. S7960, Houston, TX, USA) [48,49].

#### 3.2.3. Evaluation of In Vitro Cytotoxic Effects on the Renal Carcinoma Cell Line (RFX 393)

The MTT assay is a widely used technique that uses multiwell plates to assess in vitro cytotoxicity. To achieve the best possible outcomes, cells in the exponential growth phase were utilized, ensuring that the end cell density did not surpass 10^6^ cells/cm^2^. Every assessment included a blank, which consisted of an entire medium devoid of cells. To begin, the cultures were taken out of the incubator and moved to a laminar flow hood or another sterile workspace. Every MTT [M-5655] vial was refilled by adding 3 mL of culture medium or adjusted solution of salt without phenol red and serum, and an equivalent volume of 10% of the total medium was utilized to incorporate the reconstituted MTT into the culture. The cultures were then backed into the incubator for a duration ranging from 2 to 4 h, according to the particular type of cells and their corresponding density levels (with 2 h typically being sufficient, though longer times were sometimes needed for lower cell densities or cells that have reduced metabolic activity). Unwavering incubation periods have been maintained to allow for proper comparisons between tests. MTT Solubilization Solution (M-8910) was introduced to dissolve the resultant formazan crystals after the cell cultures were extracted from the incubator following the period of incubation in the same volume as the original culture medium. Gentle shaking or pipetting up and down, especially in denser cultures, was used to fully disperse the formazan crystals. Finally, absorbance was spectrophotometrically analyzed at 570 nm, with background absorbance at 690 nm being subtracted for accuracy. The results were read directly from the multiwell plate. The contents of each well were placed in cuvettes for measurement or analyzed using a plate reader [50].

#### 3.2.4. Cell Cycle Investigation

RFX 393 cells underwent treatment with compounds **6s** and **6t** at their respective GI50 concentrations for 48 h. Following treatment, the cells were collected, dispersed in 0.5 mL of PBS, and subjected to centrifugation. They were then preserved in 70% (*v*/*v*) ethanol at 4 °C and subsequently rinsed with PBS, treated with RNase, and propidium iodide (PI) was used to stain the cells. Flow cytometric measurements were carried out employing a FACScalibur system (Becton Dickinson, Franklin Lakes, NJ, USA). The distributions of the cell cycle were subsequently calculated with the aid of software from Phoenix Flow Systems and Verity Software House (ModFit LT V6.0) [51].

#### 3.2.5. Apoptosis and Necrosis Investigation

RFX 393 cells underwent treatment with the GI_50_ concentrations of compounds **6s** and **6t** for 48 h. Following the treatment, the cells were collected, dispersed in 0.5 mL of PBS, and subjected to centrifugation. They were then preserved in 70% (*v*/*v*) ethanol at 4 °C and subsequently rinsed with PBS. The fixed cells were then centrifuged again, resuspended in PBS, centrifuged once more, and stained with PE Annexin V and propidium iodide (PI) according to the manufacturer’s instructions. The samples were subsequently analyzed using flow cytometry with a FACScalibur instrument (Becton Dickinson, Franklin Lakes, NJ, USA). Data on cell cycle distributions were processed with the aid of Phoenix Flow Systems and Verity Software House [52].

### 3.3. Docking Protocol

Molecular docking simulations were conducted using molecular operating environment suite (MOE, 2019).

### 3.4. Computational ADME Analysis

The ADME analysis was carried out using the SWISSADME platform (Supplementary Materials Table S1).

### 3.5. Prediction of Toxicity

Toxicity prediction was carried out using the server Protox-II [40] (Supplementary Materials Table S2).

## 4. Conclusions

A novel series of 2-(anilinyl)-7-(aryl)pyrazolo[1,5-*a*]pyrimidine **6a**–**t**, 6-cyano-7-(aryl)-2-(anilinyl)pyrazolo[1,5-*a*]pyrimidine **11a**–**g**, and 7-amino-5-oxo-2-(anilinyl)-4,5-dihydropyrazolo[1,5-*a*]pyrimidine-3-carbonitrile **12** were developed, synthesized, and assessed. Their ability to suppress the growth of 60 distinct cancer cell lines was examined following the NCI protocol, and the most active compounds were subjected to CDK2 and TRKA enzyme. Compound **6n** demonstrated widespread antitumor activity, achieving a mean growth inhibition (GI) of 43.9% across 56 Neoplastic cultures.

Antiproliferative activity of the recently developed compounds revealed that compounds **6d**, **6k**, **6m**–**p**, **6r**–**t**, and **11g** were the most potent based on NCI 60 cancer cell lines. These promising results motivated our curiosity to assess their enzyme inhibition activity against CDK2 and TRKA. CDK2 showed heightened sensitivity to the effects of the evaluated compounds compared to TRKA, except compounds **6k**, **6m**, and **6s** which were more effective towards TRKA. Interestingly, compounds **6r**, **6s**, **6t**, and **11g** showed potent inhibition against CDK2 with IC_50_ of 0.20, 0.45, 0.09, and 0.22 µM, respectively. The replacement of the phenyl group in compound **6k** with the more lipophilic naphthalene substitution **6r** or hetero ring as furan **6s** and thiophen **6t** increased the activity of the compounds. Also, the addition of a cyano group at position 6 of compound **6s** increased the activity in compound **11g**.

Finally, compounds **6t** and **6s** demonstrated the greatest potency in inhibition against CDK2 and TRKA with IC_50_ of 0.09, 0.45 µM and 0.45, 0.23 µM, respectively. The findings encouraged us to further explore the effects of the synthesized pyrazolopyrimidine derivatives **6s** and **6t**. These compounds showed significant growth-inhibitory efficacy towards the RFX 393 renal carcinoma cell line, with IC_50_ = 11.70 and 19.92 µM, respectively, with **6s** showing higher cytotoxicity. Further analysis revealed that both compounds caused significant cell cycle arrest at the G0–G1 phase. Moreover, they effectively induced early and late apoptosis and increased necrosis levels compared to untreated control cells. So, it can be considered as dual kinase inhibitor and were anticipated to have favorable pharmacokinetic properties, positioning them as strong contenders for additional exploration in cancer therapy. In conclusion, this study highlights the suggested design approach and introduces a novel molecular scaffold with potential for optimization to enhance dual potency as inhibitors of CDK2 and TRKA.

## Figures and Tables

**Table 1:** Suppression effect of the developed compounds on kinases (IC_50_ µM).

Code	R	Ar	X	Enzyme Inhibition IC_50_ (µM)	
**6d**	CN	3,4-di-MeO-C_6_H_3_	H	CDK-2	TRK-A
				0.55 ± 0.04	0.57 ± 0.02
**6k**	COOEt	Ph	H	1.58 ± 0.13	1.17 ± 0.09
**6m**	COOEt	4-OCH_3_-C_6_H_4_	H	1.06 ± 0.09	0.96 ± 0.04
**6n**	COOEt	3,4-di-MeO-C_6_H_3_	H	0.78 ± 0.04	0.98 ± 0.04
**6o**	COOEt	4-Cl-C_6_H_4_	H	0.76 ± 0.04	1.59 ± 0.11
**6p**	COOEt	4-Br-C_6_H_4_	H	0.66 ± 0.04	1.34 ± 0.08
**6r**	COOEt	Naphthalen-2-yl	H	0.20 ± 0.02	0.97 ± 0.04
**6s**	COOEt	Furan-2-yl	H	0.45 ± 0.02	0.23 ± 0.01
**6t**	COOEt	Thiophene-2-yl	H	0.09 ± 0.01	0.45 ± 0.02
**11g**	COOEt	Furan-2-yl	CN	0.22 ± 0.02	0.89 ± 0.03
Ribociclib				0.07 ± 0.01	-
Larotrectinib				-	0.067 ± 0.01

**Table 2:** In vitro assessment of compounds **6s** and **6t** for their cytotoxic effects toward the renal carcinoma cell line RFX 393 (IC_50_ µM).

Code	RFX393 (IC_50_, µM)
**6s**	11.70 ± 0.4
**6t**	19.92 ± 0.67
Staurosporine	0.07

**Table 3:** Cell Cycle investigation of compounds **6s** and **6t** on RFX 393 cell line.

Compound	%G0–G1	%S	%G2/M
**6s**/RFX 393	84.36	11.49	4.15
**6t**/RFX 393	78.01	15.26	6.73
RFX 393 Control	57.08	29.33	13.59

**Table 4:** Apoptosis and necrosis investigation of compounds 6s and 6t on RFX 393 cell line.

	Apoptosis			Necrosis
Compound	Total	Early	Late	
**6s**/RFX 393	32.07	20.29	7.74	4.04
**6t**/RFX 393	24.22	11.15	9.49	3.58
RFX 393 Control	2.71	0.64	0.15	1.92

**Table 5:** Docking energy of the newly developed compounds scores (S) in kcal·mol⁻¹ compared to enzyme inhibition results.

	CDK2		TRKA	
Compound	Docking Score (S) kcal.mol^−1^	(IC_50_, µM)	Docking Score (S) kcal.mol^−1^	(IC_50_, µM)
**6d**	−6.2514	0.55	−6.3710	0.57
**6k**	−7.3301	1.58	−7.4962	1.17
**6m**	−6.4495	1.06	−7.0142	0.96
**6n**	−6.4894	0.78	−7.6633	0.98
**6o**	−5.8055	0.76	−7.4024	1.59
**6p**	−6.6507	0.66	−7.5902	1.34
**6r**	−6.0688	0.20	−7.1326	0.97
**6s**	−5.8138	0.45	−7.0673	0.23
**6t**	−6.8289	0.09	−6.3594	0.45
**11g**	−5.9020	0.22	−7.3838	0.89
**Ribociclib**		0.07		-
**Larotrectinib**		-		0.067

## Data Availability

Data are contained within the article and Supplementary Materials.

## Supplementary Materials

The following supporting information can be downloaded at: https://www.mdpi.com/article/10.3390/ph17121667/s1, Table S1. (Virtual ADME assessment); Table S2. (Prediction of toxicity); Figures S1–S49 (^1^H NMR and ^13^C NMR spectra of **12a**–**t**, **19a**–**g**, and **22**); Figures S50–S77. (NCI panel cell line Mean graph).
