# Supplementary material for: Design, Synthesis and Molecular Modeling of Pyrazolo[1,5-a]pyrimidine Derivatives as Dual Inhibitors of CDK2 and TRKA Kinases with Antiproliferative Activity
Source: Pharmaceuticals (Basel). 2024 Dec 10;17(12):1667. doi: 10.3390/ph17121667 (PMC11678221; doi:10.3390/ph17121667)
Supplement: Supplementary file 1 [file pharmaceuticals-17-01667-s001.zip › pharmaceuticals-3317802-supplementary.pdf]

Supplementary data

Design, Synthesis and Molecular Modeling of Pyrazolo[1,5-*a*]pyrimidine Derivatives as Dual Inhibitors of CDK2 and TRKA Kinases with Antiproliferative Activity

Mohamed H. Attia [1,*], Deena S. Lasheen [2], Nermin Samir [2], Azza T. Taher [3,4], Hatem A. Abdel-Aziz [5,6] and Dalal A. Abou El Ella [2,*]

[1] Department of Pharmaceutical Chemistry, Faculty of Pharmacy, October 6 University (O6U), Giza 12585, Egypt
[2] Pharmaceutical Chemistry Department, Faculty of Pharmacy, Ain Shams University, Cairo 11566, Egypt
[3] Department of Pharmaceutical Organic Chemistry, Faculty of Pharmacy, Cairo University, Cairo 11562, Egypt
[4] Department of Pharmaceutical Organic Chemistry, Faculty of Pharmacy, October 6 University (O6U), Giza 12585, Egypt
[5] Department of Applied Organic Chemistry, National Research Center, Cairo 12622, Egypt
[6] Department of Pharmaceutical Chemistry, Faculty of Pharmacy, Pharos University in Alexandria, Canal El Mahmoudia St., Alexandria 21648, Egypt
* Correspondence: mohammed_attia@o6u.edu.eg (M.H.A.); dalal@pharma.asu.edu.eg (D.A.A.E.E.)

Content

Virtual ADME assessment	
**Table S1.** Computer aided ADME screening of the all synthesized compounds………………….....	**5**
**Table S2.** Toxicity Prediction of the all newly synthesized compounds……….…………………….	**6**
**1H NMR and 13C NMR spectra**	
**Figure S1.** ^{1}H NMR of compound **6a**………………………………………………………………	**9**
**Figure S2.** ^{13}C NMR of compound **6a**…………..………………………..…………………………	**9**
**Figure S3.** ^{1}H NMR of compound **6b**………………………………………………………………	**10**
**Figure S4.** ^{13}C NMR of compound **6b**…………..………………………..…………………………	**10**
**Figure S5.** ^{1}H NMR of compound **6c**………………………………………………………………	**11**
**Figure S6.** ^{13}C NMR of compound **6c**…………..………………………..…………………………	**11**
**Figure S7.** ^{1}H NMR of compound **6d**………………………………………………………………	**12**
**Figure S8.** ^{13}C NMR of compound **6d**…………..………………………..…………………………	**12**
**Figure S9.** ^{1}H NMR of compound **6e**………………………………………………………………	**13**
**Figure S10.** ^{13}C NMR of compound **6e**…………..………………………..…………………………	**13**
**Figure S11.** ^{1}H NMR of compound **6f**………………………………………………………………	**14**
**Figure S12.** ^{13}C NMR of compound **6f**…………..………………………..…………………………	**14**
**Figure S13.** ^{1}H NMR of compound **6g**………………………………………………………………	**15**
**Figure S14.** ^{1}H NMR of compound **6h**………………………………………………………………	**15**
**Figure S15.** ^{13}C NMR of compound **6h**…………..………………………..…………………………	**16**
**Figure S16.** ^{1}H NMR of compound **6i**………………………………………………………………	**16**
**Figure S17.** ^{1}H NMR of compound **6j**………………………………………………………………	**17**
**Figure S18.** ^{13}C NMR of compound **6j**…………..………………………..…………………………	**17**
**Figure S19.** ^{1}H NMR of compound **6k**………………………………………………………………	**18**
**Figure S20.** ^{13}C NMR of compound **6k**…………..………………………..…………………………	**18**
**Figure S21.** ^{1}H NMR of compound **6l**………………………………………………………………	**19**
**Figure S22.** ^{1}H NMR of compound **6m**………………………………………………………………	**19**
**Figure S23.** ^{13}C NMR of compound **6m**…………..………………………..…………………………	**20**
**Figure S24.** ^{1}H NMR of compound **6n**………………………………………………………………	**20**
**Figure S25.** ^{13}C NMR of compound **6n**…………..………………………..…………………………	**21**
**Figure S26.** ^{1}H NMR of compound **6o**………………………………………………………………	**21**
**Figure S27.** ^{1}H NMR of compound **6p**………………………………………………………………	**22**
**Figure S28.** ^{13}C NMR of compound **6p**…………..………………………..…………………………	**22**
**Figure S29.** ^{1}H NMR of compound **6q**………………………………………………………………	**23**
**Figure S30.** ^{13}C NMR of compound **6q**…………..………………………..…………………………	**23**
**Figure S31.** ^{1}H NMR of compound **6r**………………………………………………………………	**24**
**Figure S32.** ^{13}C NMR of compound **6r**…………..………………………..…………………………	**24**
**Figure S33.** ^{1}H NMR of compound **6s**………………………………………………………………	**25**
**Figure S34.** ^{13}C NMR of compound **6s**…………..………………………..…………………………	**25**
**Figure S35.** ^{1}H NMR of compound **6t**………………………………………………………………	**26**
**Figure S36.** ^{13}C NMR of compound **6t**…………..………………………..…………………………	**26**

**Figure S37.** ^{1}H NMR of compound **11a**…………………………………………………………	27
**Figure S38.** ^{13}C NMR of compound **11a**………..……………………..…………………………	27
**Figure S39.** ^{1}H NMR of compound **11b**…………………………………………………………	28
**Figure S40.** ^{13}C NMR of compound **11b**………..……………………..…………………………	28
**Figure S41.** ^{1}H NMR of compound **11c**…………………………………………………………	29
**Figure S42.** ^{13}C NMR of compound **11c**………..……………………..…………………………	29
**Figure S43.** ^{1}H NMR of compound **11d**…………………………………………………………	30
**Figure S44.** ^{13}C NMR of compound **11d**………..……………………..…………………………	30
**Figure S45.** ^{1}H NMR of compound **11e**…………………………………………………………	31
**Figure S46.** ^{1}H NMR of compound **11f**…………………………………………………………	31
**Figure S47.** ^{1}H NMR of compound **11g**…………………………………………………………	32
**Figure S48.** ^{1}H NMR of compound **12**…………………………………………………………	32
**Figure S49.** ^{13}C NMR of compound **12**………..……………………..…………………………	33
**NCI panel cell line Mean graph**	
**Figure S50.** Mean graph of compound (**6a**) with colour codes for each cell line……………………	34
**Figure S51.** Mean graph of compound (**6b**) with colour codes for each cell line……………………	35
**Figure S52.** Mean graph of compound (**6c**) with colour codes for each cell line……………………	36
**Figure S53.** Mean graph of compound (**6d**) with colour codes for each cell line……………………	37
**Figure S54.** Mean graph of compound (**6e**) with colour codes for each cell line……………………	38
**Figure S55.** Mean graph of compound (**6f**) with colour codes for each cell line……………………	39
**Figure S56.** Mean graph of compound (**6g**) with colour codes for each cell line……………………	40
**Figure S57.** Mean graph of compound (**6h**) with colour codes for each cell line……………………	41
**Figure S58.** Mean graph of compound (**6i**) with colour codes for each cell line……………………	42
**Figure S59.** Mean graph of compound (**6j**) with colour codes for each cell line……………………	43
**Figure S60.** Mean graph of compound (**6k**) with colour codes for each cell line……………………	44
**Figure S61.** Mean graph of compound (**6l**) with colour codes for each cell line……………………	45
**Figure S62.** Mean graph of compound (**6m**) with colour codes for each cell line…………………	46
**Figure S63.** Mean graph of compound (**6n**) with colour codes for each cell line…………………	47
**Figure S64.** Mean graph of compound (**6o**) with colour codes for each cell line…………………	48
**Figure S65.** Mean graph of compound (**6p**) with colour codes for each cell line…………………	49
**Figure S66.** Mean graph of compound (**6q**) with colour codes for each cell line…………………	50
**Figure S67.** Mean graph of compound (**6r**) with colour codes for each cell line…………………	51
**Figure S68.** Mean graph of compound (**6s**) with colour codes for each cell line…………………	52
**Figure S69.** Mean graph of compound (**6t**) with colour codes for each cell line…………………	53
**Figure S70.** Mean graph of compound (**11a**) with colour codes for each cell line…………………	54
**Figure S71.** Mean graph of compound (**11b**) with colour codes for each cell line…………………	55
**Figure S72.** Mean graph of compound (**11c**) with colour codes for each cell line…………………	56
**Figure S73.** Mean graph of compound (**11d**) with colour codes for each cell line…………………	57
**Figure S74.** Mean graph of compound (**11e**) with colour codes for each cell line…………………	58
**Figure S75.** Mean graph of compound (**11f**) with colour codes for each cell line…………………	59
**Figure S76.** Mean graph of compound (**11g**) with colour codes for each cell line…………………	60
**Figure S77.** Mean graph of compound (**12**) with colour codes for each cell line…………………	61

Virtual ADME assessment

Table S1. Computer aided ADME screening of the all synthesized compounds:

	Parameters									
**CPD ID**	**GI Absorption**	**BBB permeation**	**Pg-p substrate**	**CYP1 A2 inhibitor**	**CYP2C 19 inhibitor**	**CYP2C 9 inhibitor**	**CYP2 D6 inhibitor**	**CYP3 A4 inhibitor**	**Logkp (Skin permeation)**	**Bioavailability score**
**6a**	High	Yes	No	Yes	Yes	Yes	Yes	Yes	-5.27	0.55
**6b**	High	Yes	No	Yes	Yes	Yes	Yes	Yes	-5.09	0.55
**6c**	High	Yes	No	Yes	Yes	Yes	Yes	Yes	-5.47	0.55
**6d**	High	No	No	Yes	Yes	Yes	Yes	Yes	-5.67	0.55
**6e**	High	Yes	No	Yes	Yes	Yes	Yes	Yes	-5.03	0.55
**6f**	High	Yes	No	Yes	Yes	Yes	Yes	Yes	-5.26	0.55
**6g**	High	No	No	Yes	No	Yes	Yes	Yes	-5.66	0.55
**6h**	High	No	Yes	Yes	Yes	Yes	Yes	No	-4.68	0.55
**6i**	High	No	No	Yes	Yes	Yes	Yes	Yes	-5.84	0.55
**6j**	High	No	No	Yes	Yes	Yes	Yes	Yes	-5.5	0.55
**6k**	High	Yes	No	Yes	Yes	Yes	Yes	No	-5.2	0.55
**6l**	High	Yes	No	Yes	Yes	Yes	Yes	Yes	-5.02	0.55
**6m**	High	No	No	Yes	Yes	Yes	Yes	Yes	-5.4	0.55
**6n**	High	No	No	Yes	Yes	Yes	Yes	Yes	-5.6	0.55
**6o**	High	No	No	Yes	Yes	Yes	No	No	-4.96	0.55
**6p**	High	No	No	Yes	Yes	Yes	No	No	-5.18	0.55
**6q**	High	No	No	Yes	Yes	Yes	No	Yes	-5.59	0.55
**6r**	High	No	No	No	Yes	Yes	No	No	-4.62	0.55
**6s**	High	No	No	Yes	Yes	Yes	Yes	Yes	-5.78	0.55
**6t**	High	No	No	Yes	Yes	Yes	No	Yes	-5.43	0.55
**11a**	High	No	No	Yes	No	Yes	Yes	Yes	-5.62	0.55
**11b**	High	No	No	Yes	Yes	Yes	Yes	Yes	-5.61	0.55
**11c**	High	No	No	Yes	No	Yes	Yes	Yes	-6.2	0.55

**11d**	High	No	No	Yes	Yes	Yes	No	Yes	-5.55	0.55
**11e**	High	No	No	Yes	Yes	Yes	No	Yes	-5.75	0.55
**11f**	High	No	No	Yes	Yes	Yes	No	Yes	-5.59	0.55
**11g**	High	No	No	Yes	No	Yes	No	Yes	-6.13	0.55
**12**	High	No	No	Yes	No	No	No	No	-6.94	0.55

Table S2. Toxicity Prediction of the all newly synthesized compounds

	**Predicted target**					
**Compound**	**Hepatotoxicity**	**Carcinogenicity**	**Immunotoxicity**	**Mutagenicity**	**Cytotoxicity**	**Acute toxicity**
**6a**	Active	Active	Inactive	Active	Inactive	Class 3
**6b**	Active	Active	Inactive	Active	Inactive	Class 3
**6c**	Active	Active	Inactive	Active	Inactive	Class 3
**6d**	Active	Active	Inactive	Active	Active	Class 3
**6e**	Active	Inactive	Inactive	Inactive	Inactive	Class 3
**6f**	Active	Inactive	Inactive	Inactive	Active	Class 3
**6g**	Active	Active	Inactive	Active	Inactive	Class 3
**6h**	Active	Active	Inactive	Active	Inactive	Class 3
**6i**	Active	Active	Inactive	Active	Inactive	Class 3
**6j**	Active	Active	Inactive	Active	Inactive	Class 4
**6k**	Inactive	Active	Inactive	Inactive	Active	Class 3
**6l**	Inactive	Active	Inactive	Inactive	Active	Class 3
**6m**	Inactive	Active	Inactive	Active	Inactive	Class 3
**6n**	Inactive	Active	Inactive	Active	Inactive	Class 4
**6o**	Inactive	Inactive	Inactive	Inactive	Inactive	Class 3
**6p**	Inactive	Inactive	Inactive	Inactive	Active	Class 3
**6q**	Inactive	Active	Inactive	Active	Inactive	Class 3
**6r**	Inactive	Active	Inactive	Inactive	Active	Class 3
**6s**	Inactive	Active	Inactive	Inactive	Inactive	Class 3

**6t**	Inactive	Active	Inactive	Inactive	Inactive	Class 4
**11a**	Active	Active	Inactive	Active	Inactive	Class 3
**11b**	Inactive	Active	Inactive	Active	Inactive	Class 3
**11c**	Active	Active	Inactive	Active	Inactive	Class 3
**11d**	Inactive	Active	Inactive	Inactive	Active	Class 3
**11e**	Inactive	Active	Inactive	Active	Inactive	Class 3
**11f**	Active	Inactive	Inactive	Inactive	Active	Class 3
**11g**	Inactive	Active	Inactive	Inactive	Inactive	Class 3
**12**	Active	Active	Inactive	Active	Inactive	Class 4

1H NMR and 13C NMR spectra

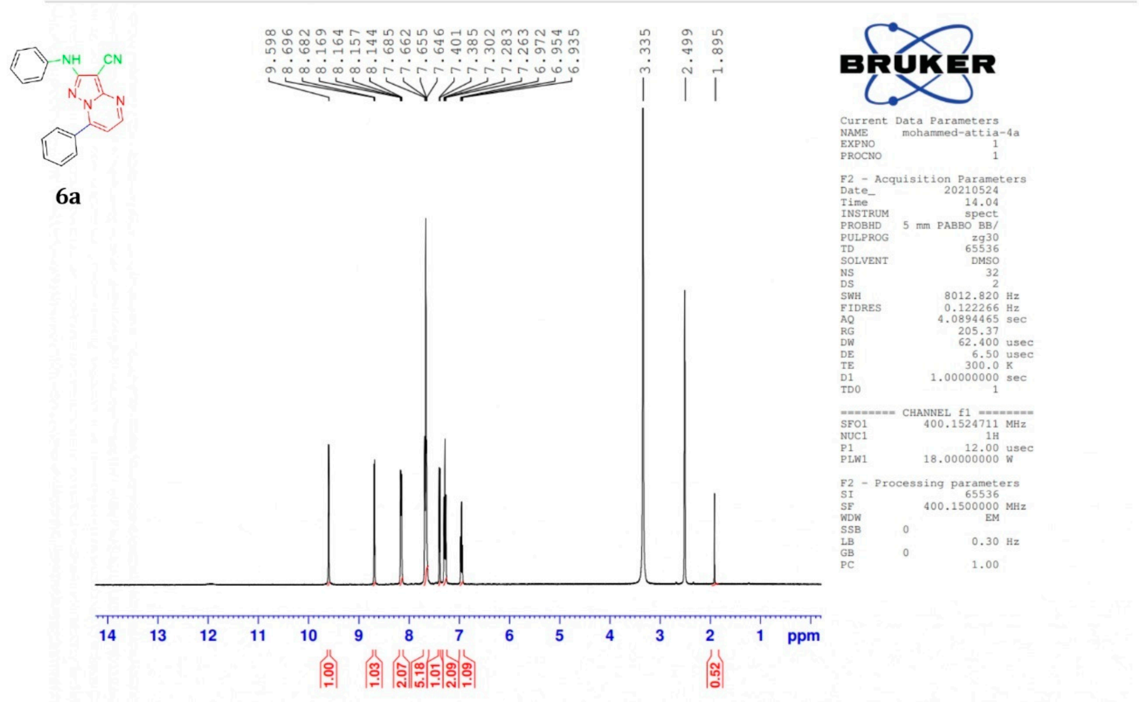

Figure S1. ^{1}H NMR of compound **6a.**

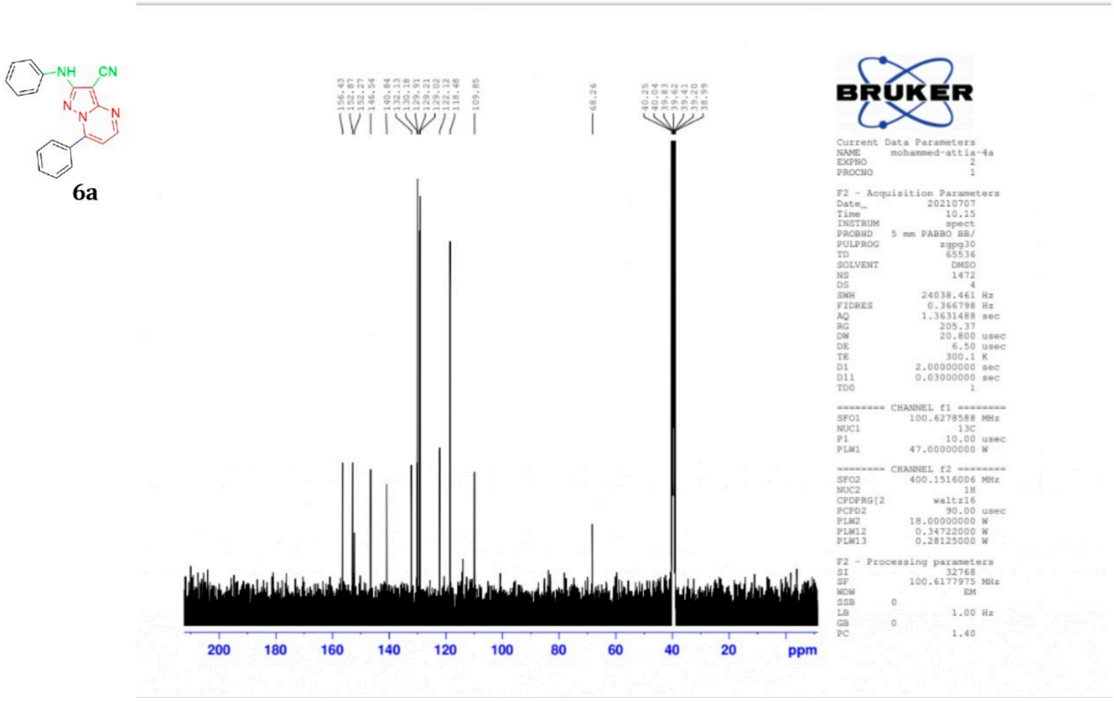

Figure S2. ^{13}C NMR of compound **6a**

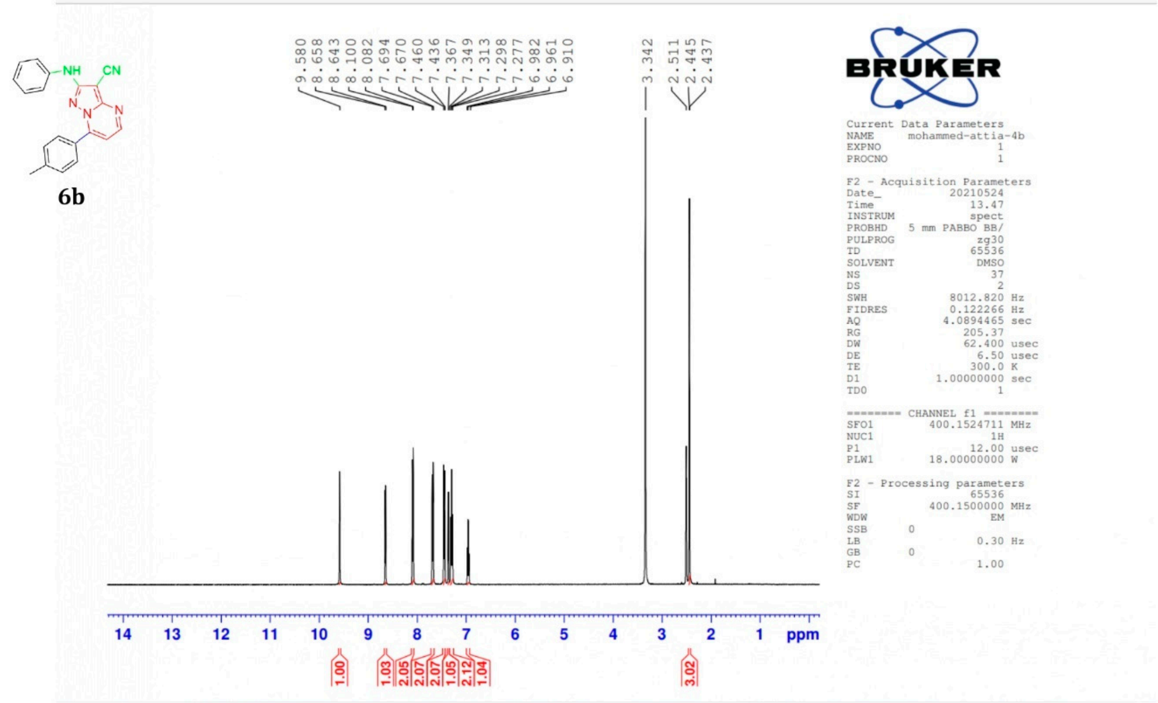

Figure S3. ^{1}H NMR of compound **6b**

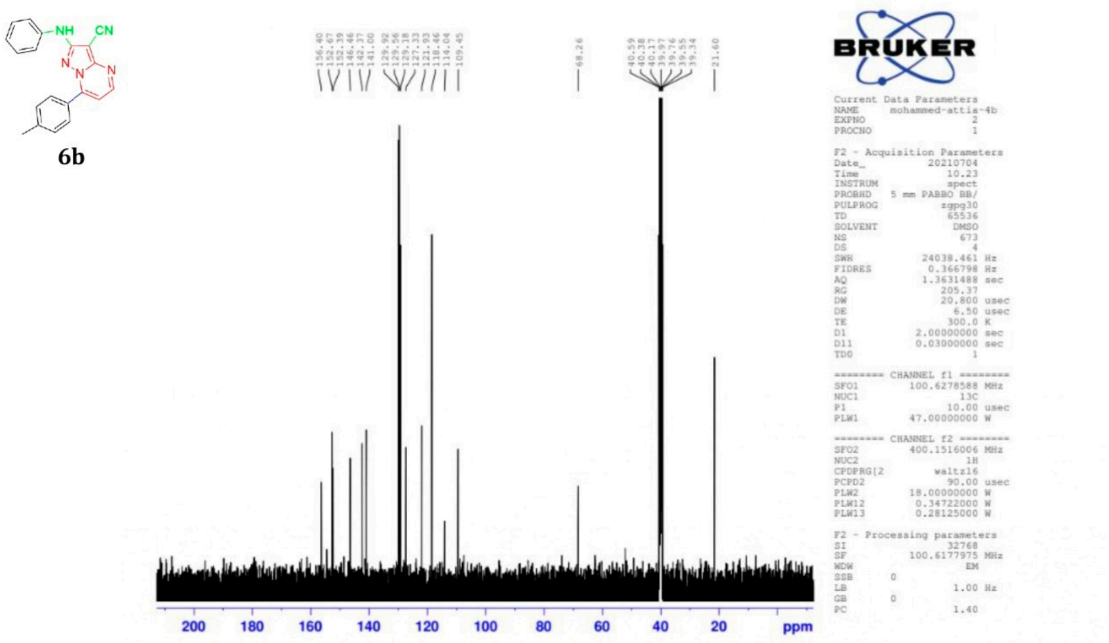

Figure S4. ^{13}C NMR of compound **6b**

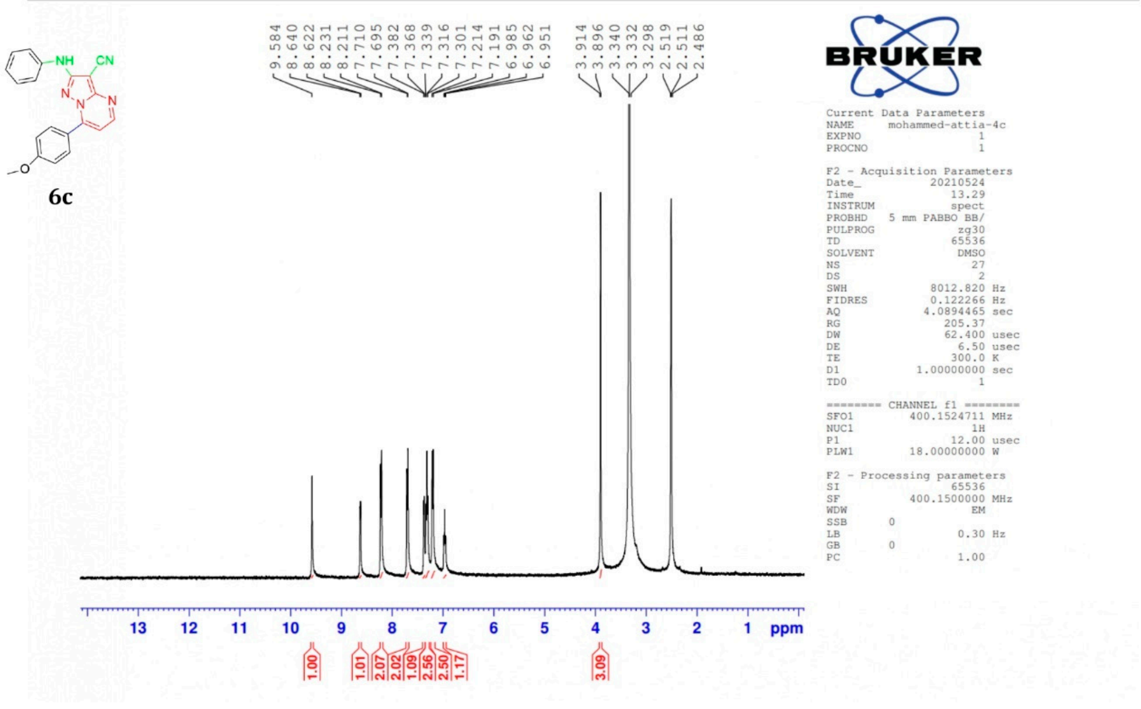

Figure S5. ^{1}H NMR of compound **6c**

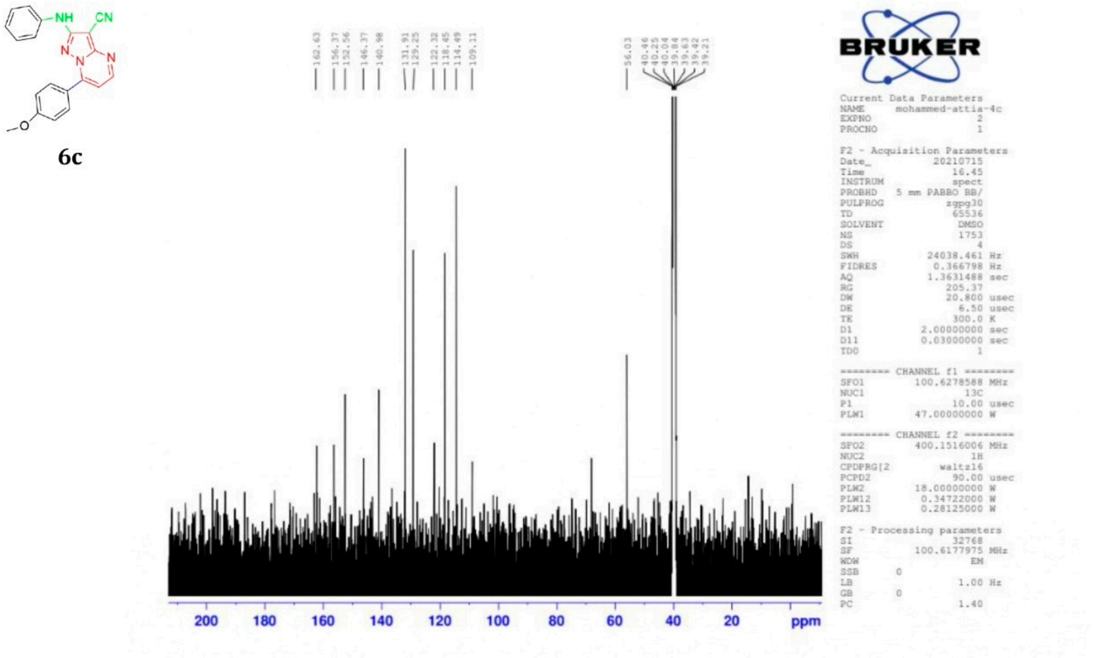

Figure S6. ^{13}C NMR of compound **6c**

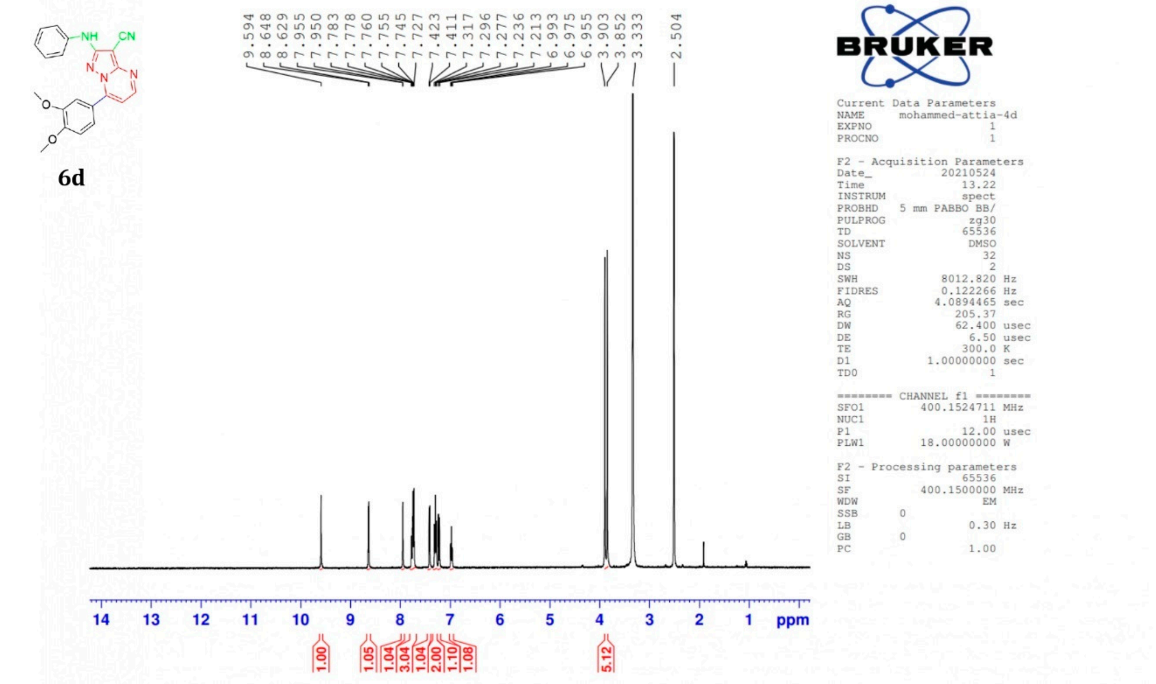

Figure S7. ^{1}H NMR of compound **6d**

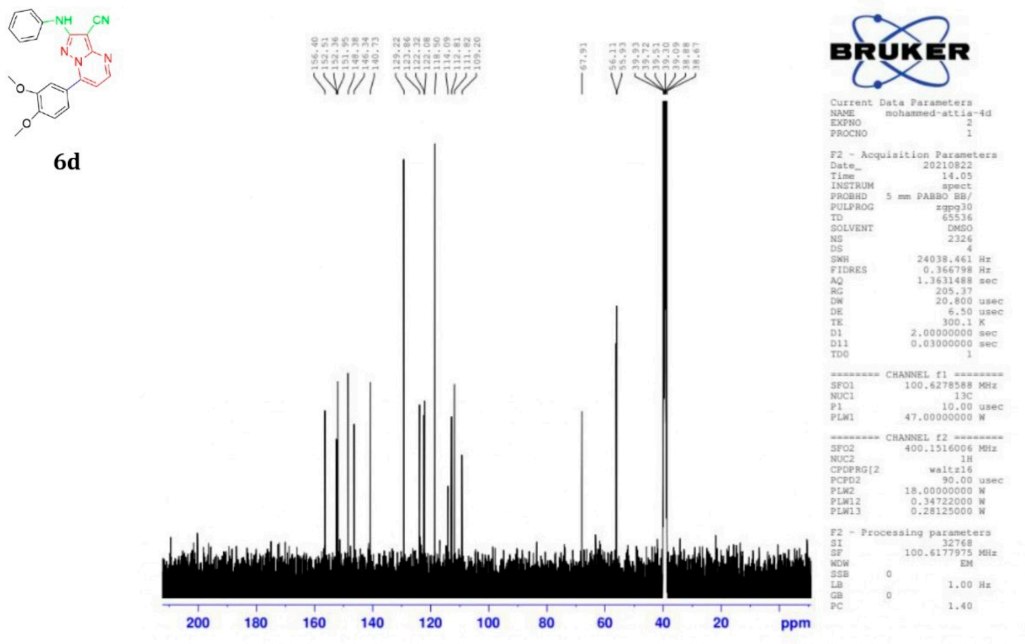

Figure S8. ^{13}C NMR of compound **6d**

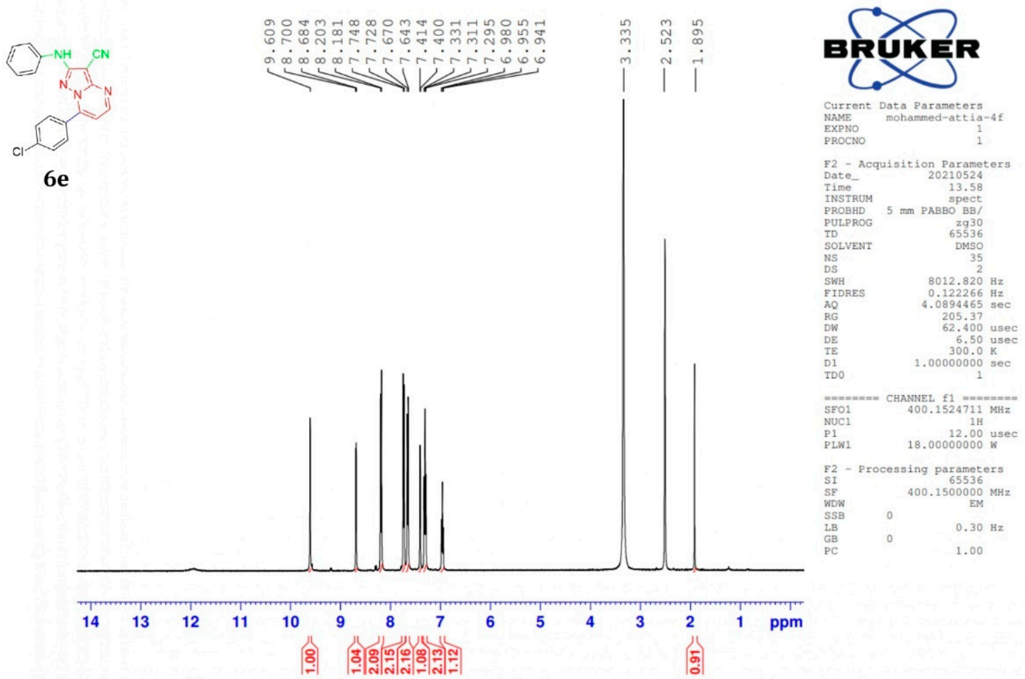

Figure S9. ^{1}H NMR of compound **6e**

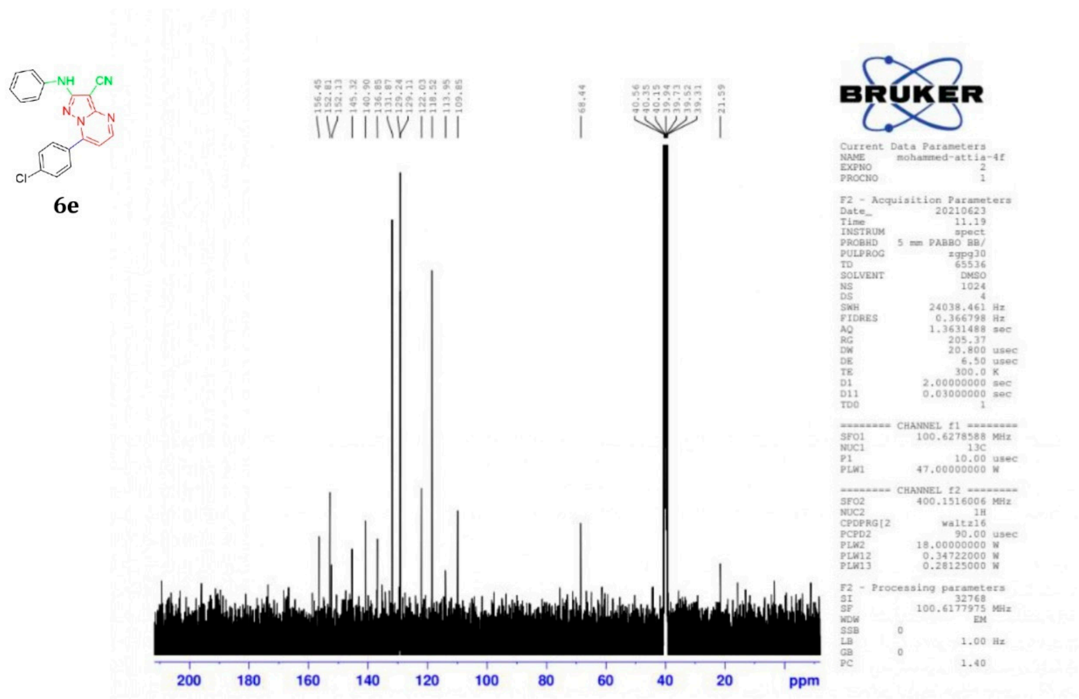

Figure S10. ^{13}C NMR of compound **6e**

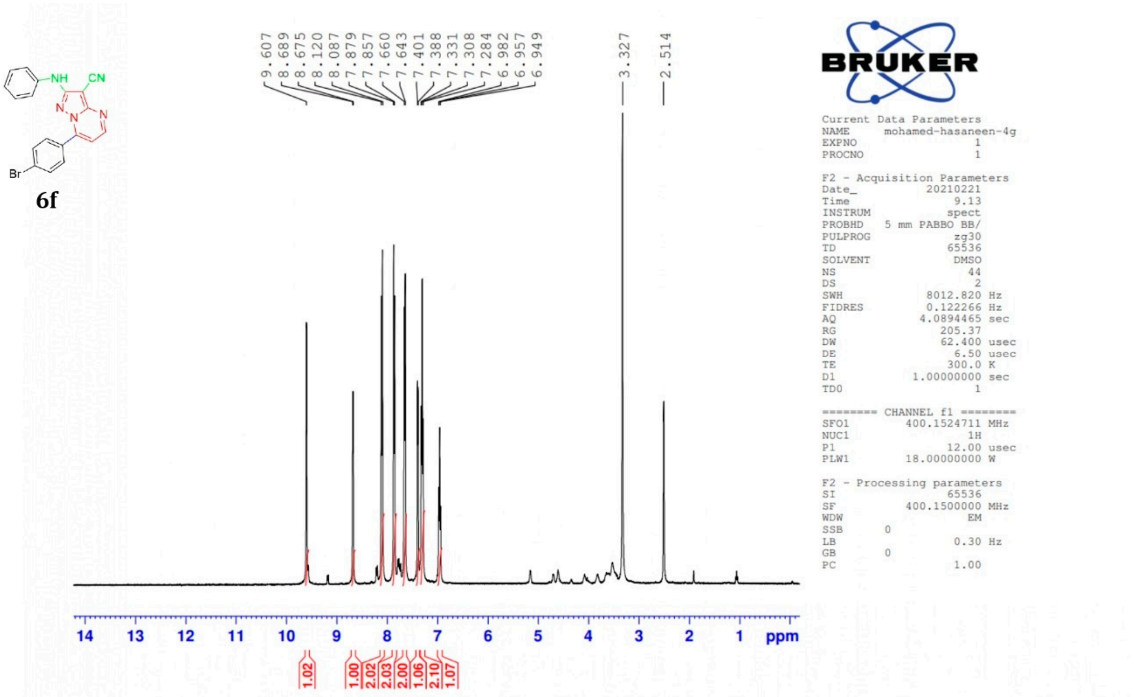

Figure S11. ^{1}H NMR of compound **6f**

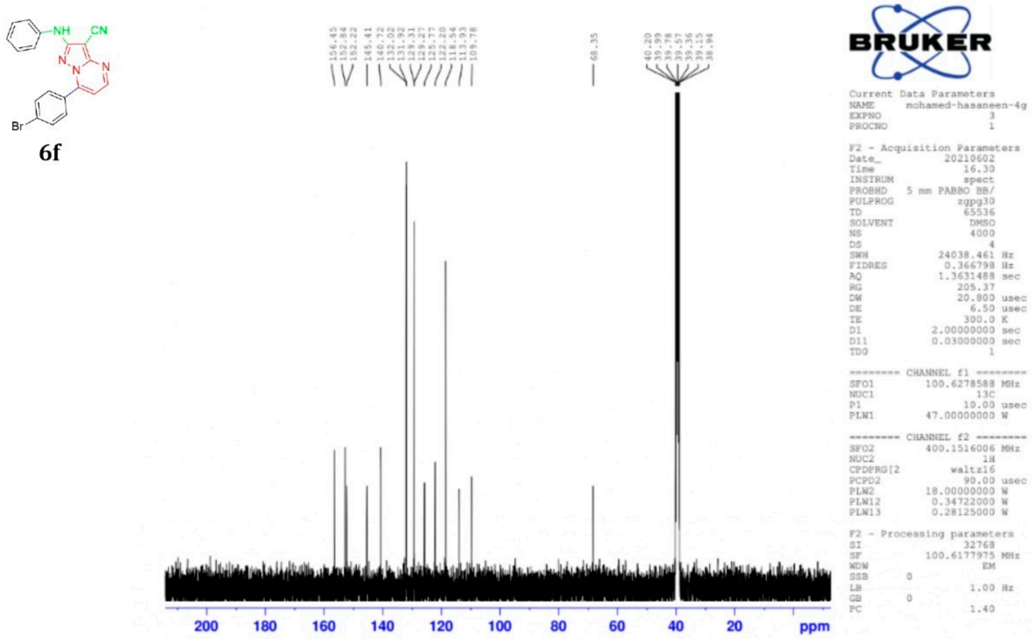

Figure S12. ^{13}C NMR of compound **6f**

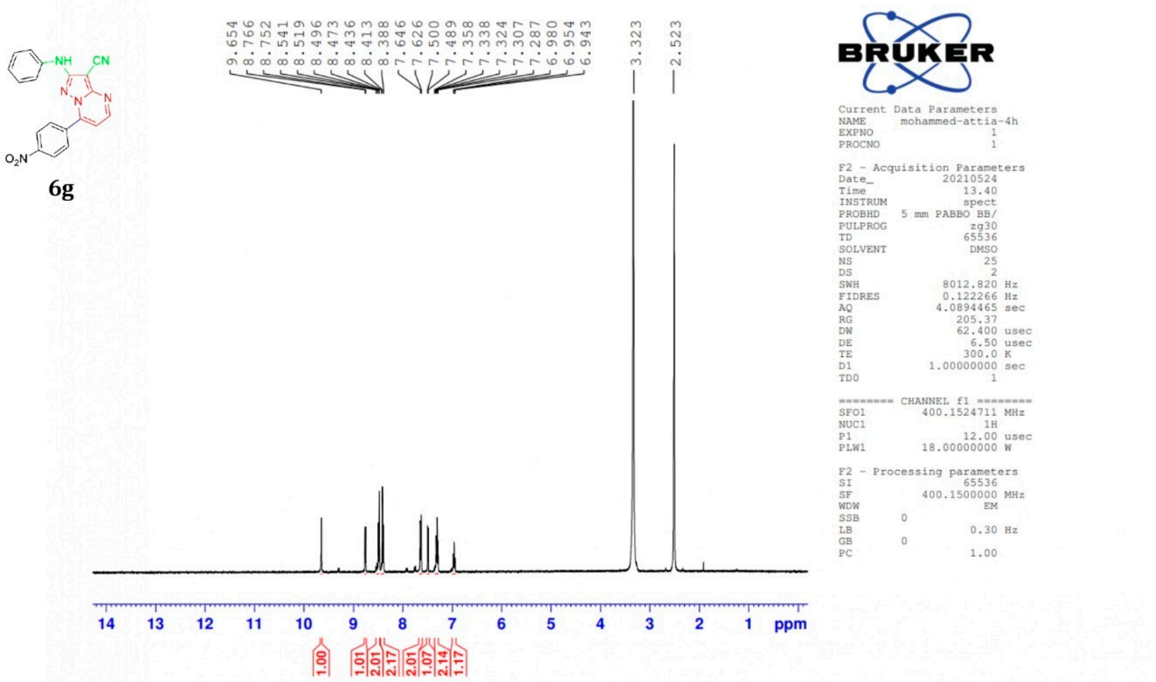

Figure S13. ^{1}H NMR of compound **6g**

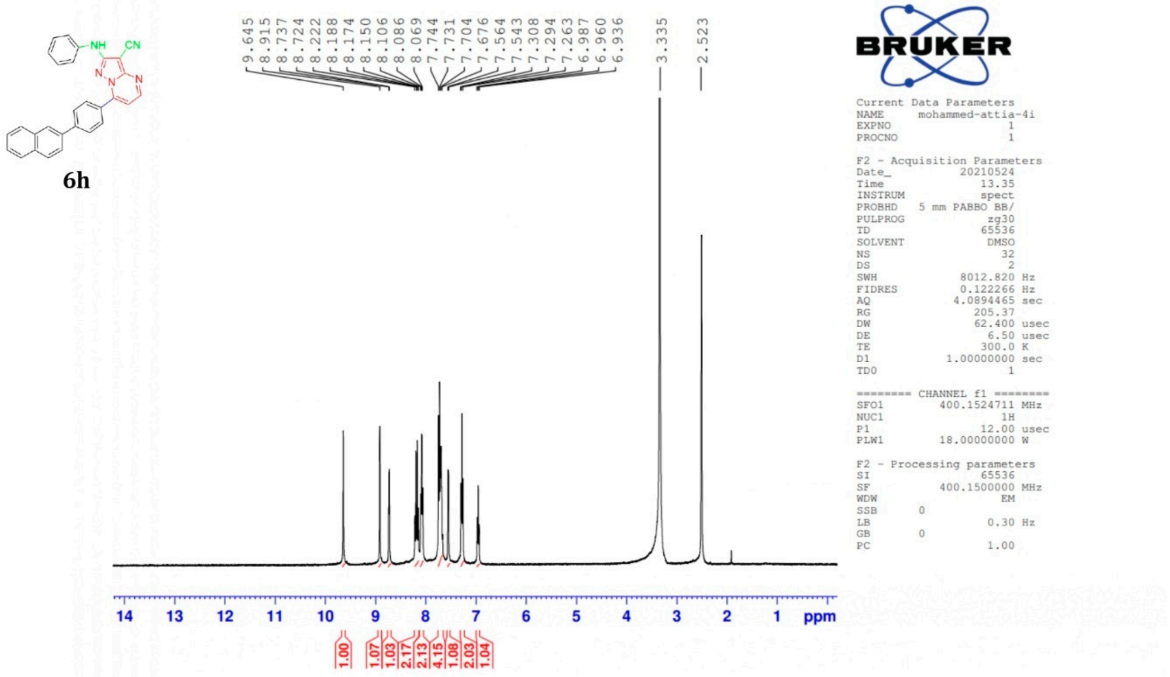

6h

Figure S14. ^{1}H NMR of compound **6h**

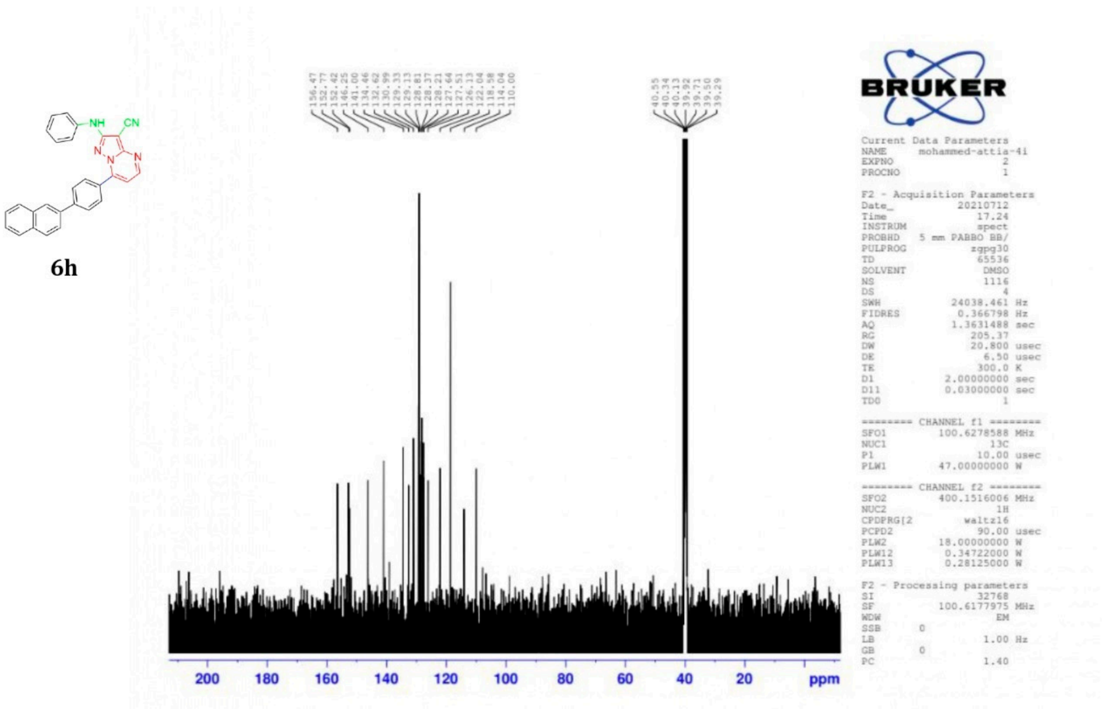

6h

Figure S15. ^{13}C NMR of compound **6h**

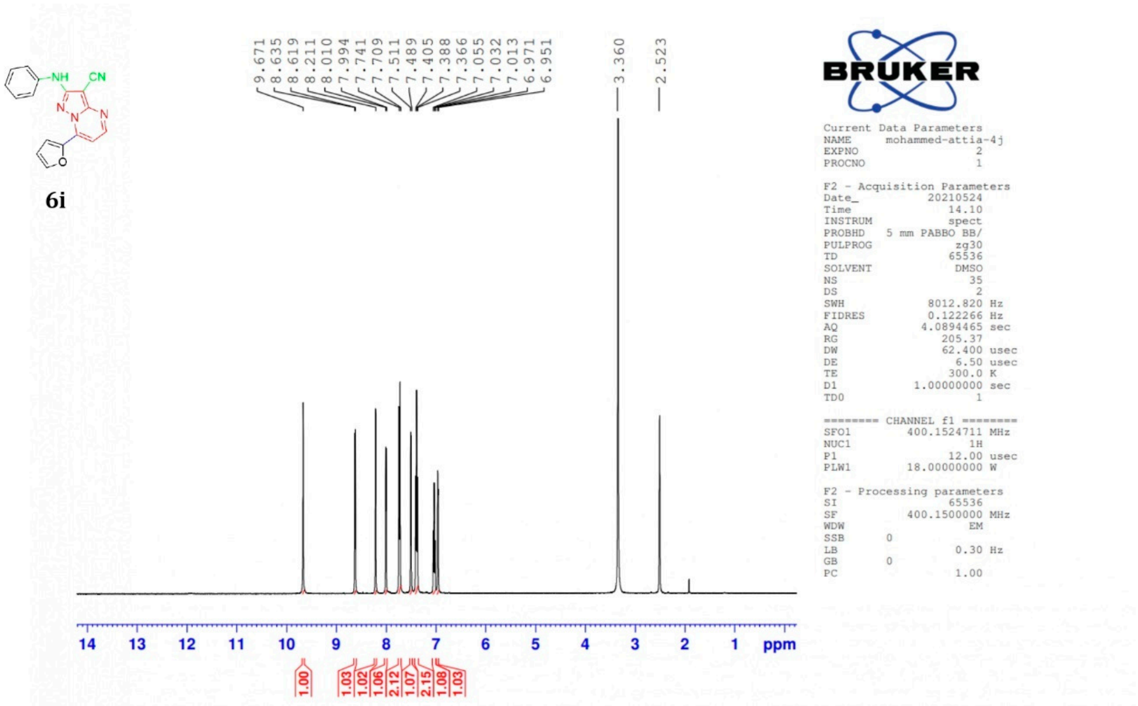

Figure S16. ^{1}H NMR of compound **6i**

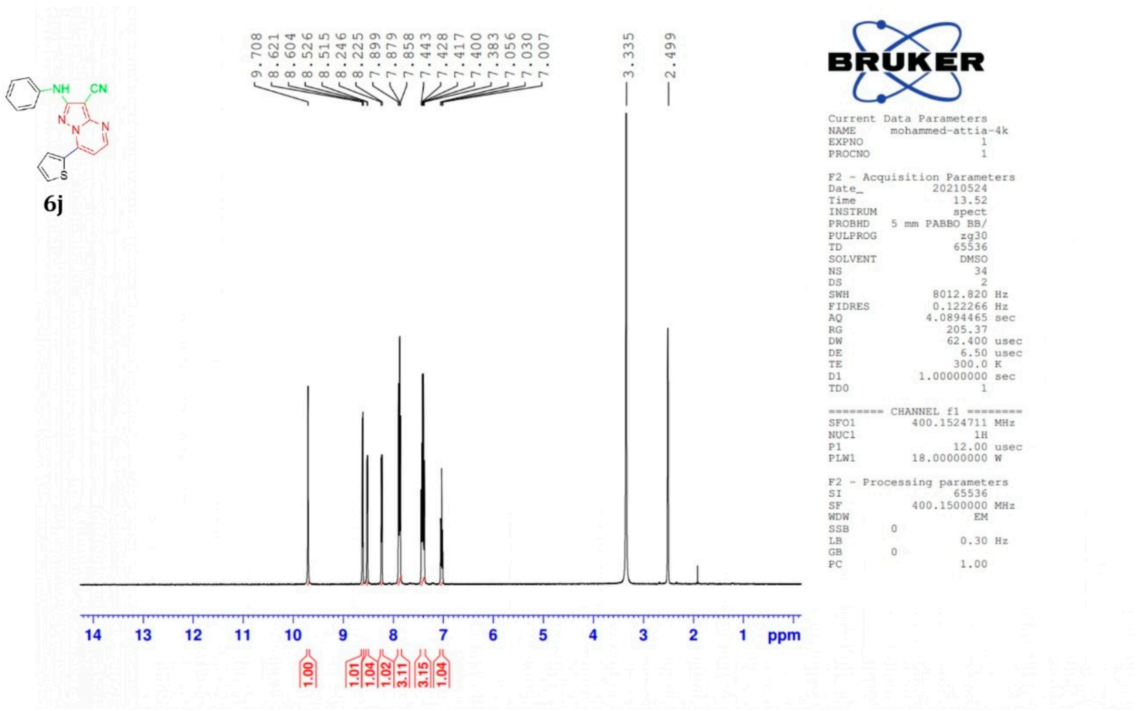

Figure S17. ^{1}H NMR of compound **6j**

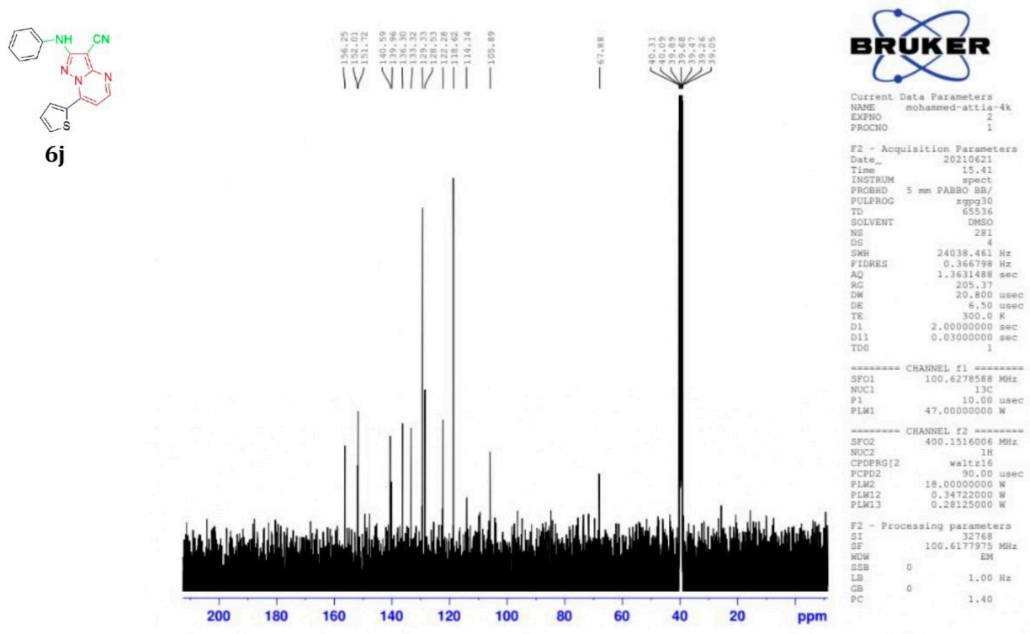

Figure S18. ^{13}C NMR of compound **6j**

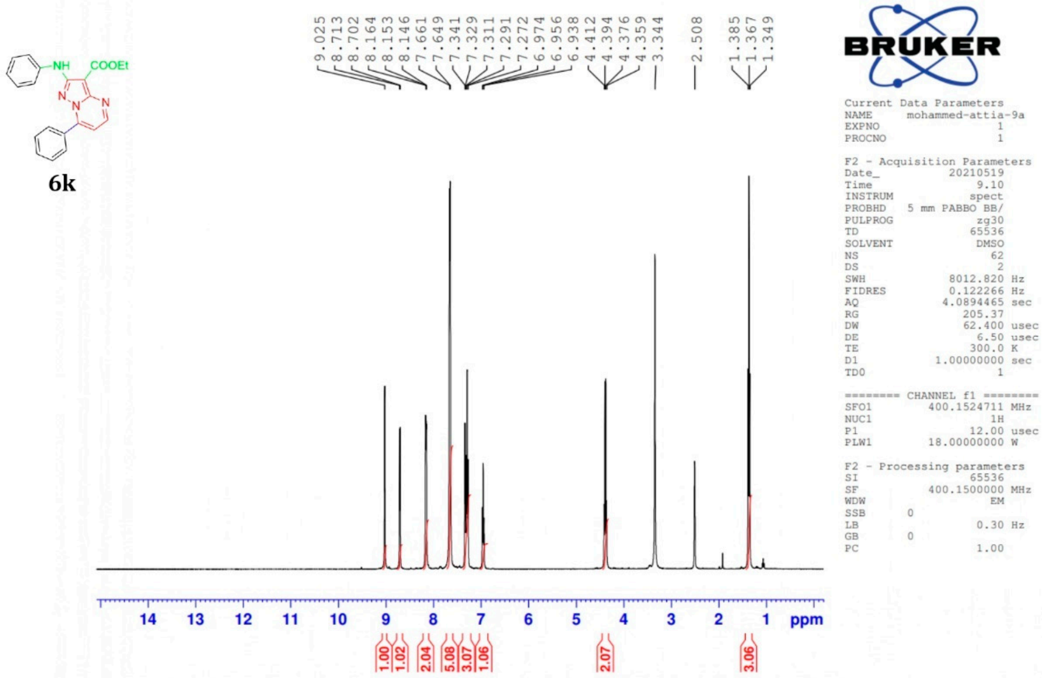

Figure S19. ^{1}H NMR of compound **6k**

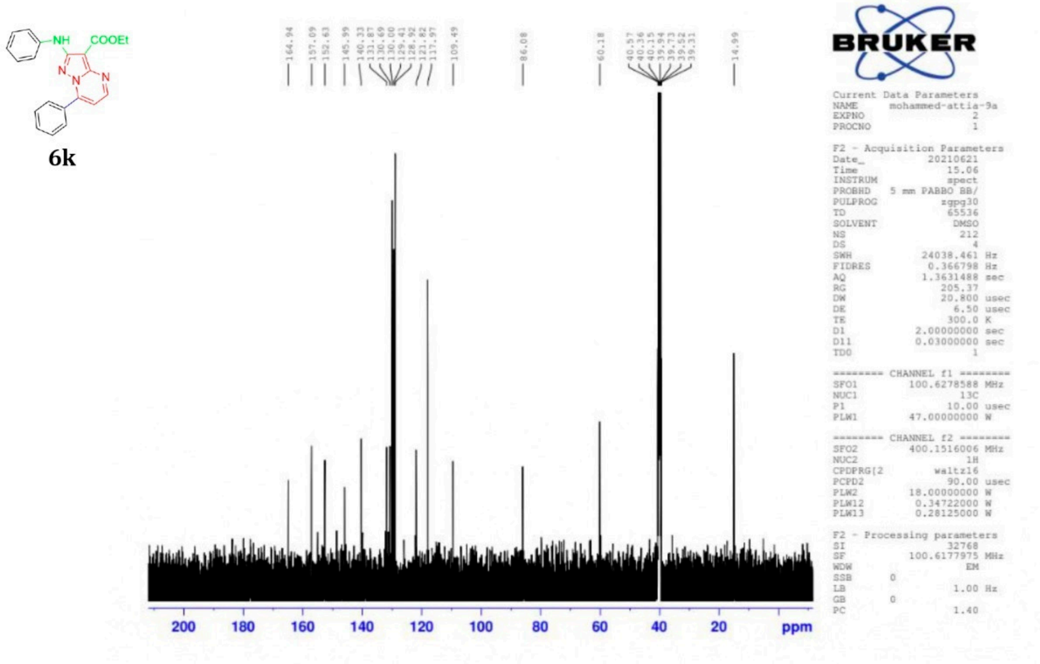

Figure S20. ^{13}C NMR of compound **6k**

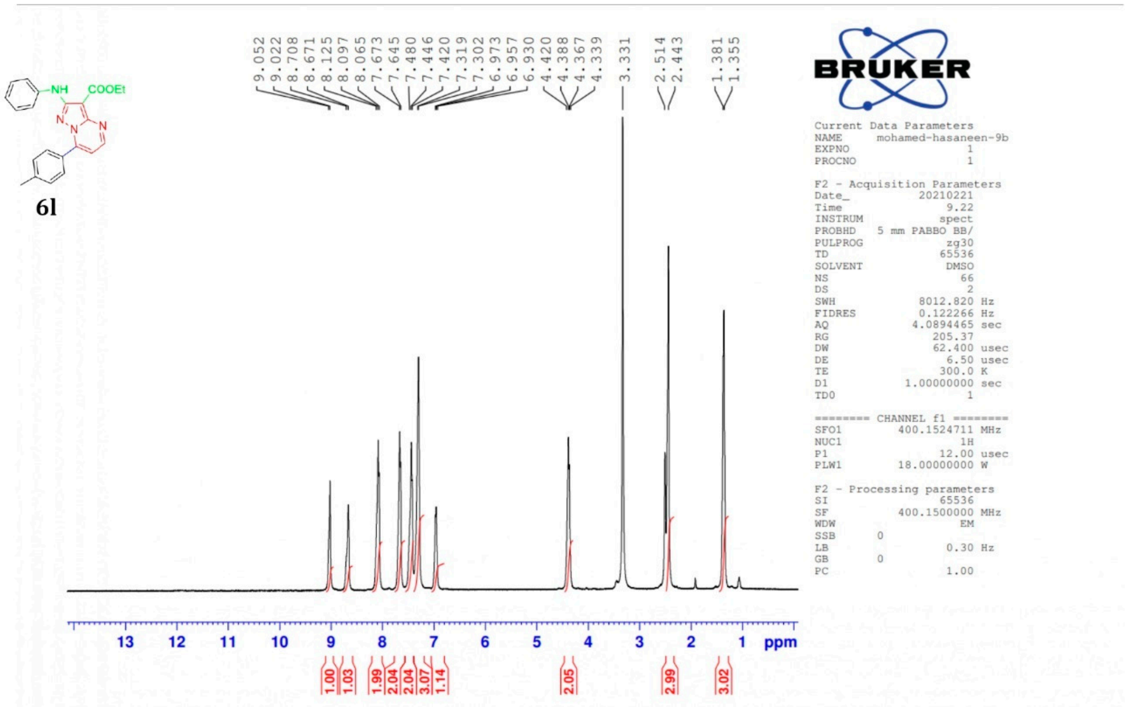

Figure S21. ^{1}H NMR of compound **6l**

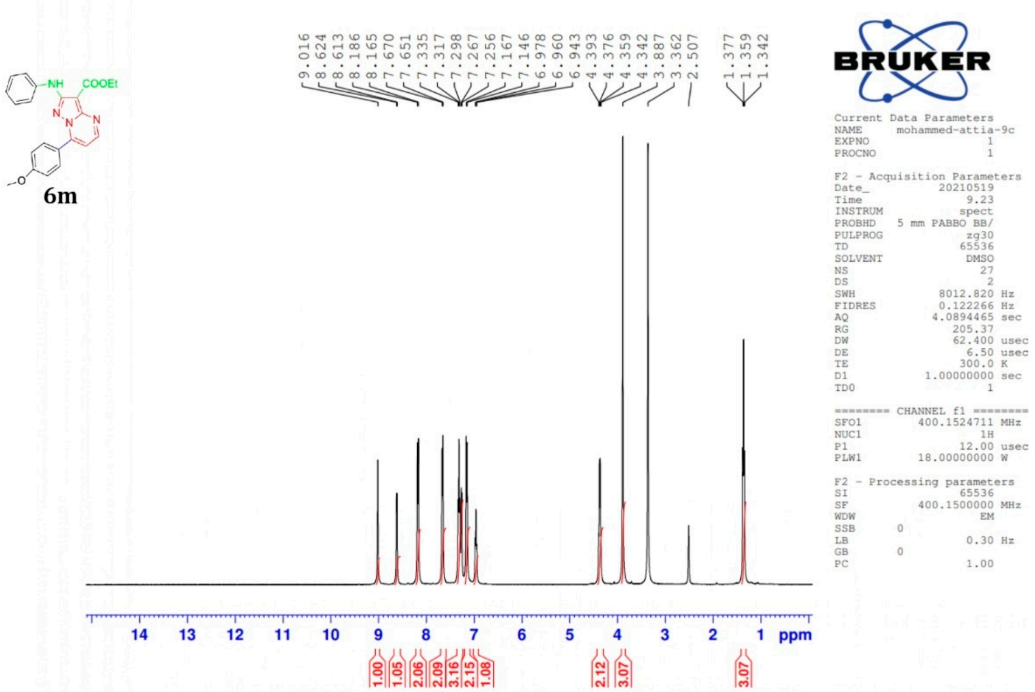

Figure S22. ^{1}H NMR of compound **6m**

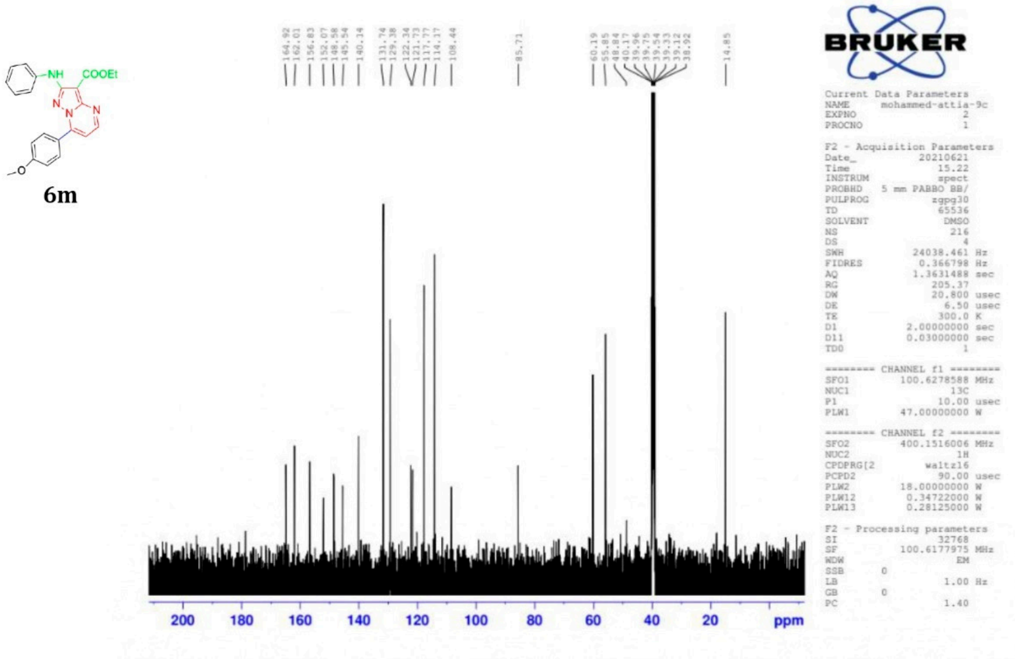

Figure S23. ^{13}C NMR of compound **6m**

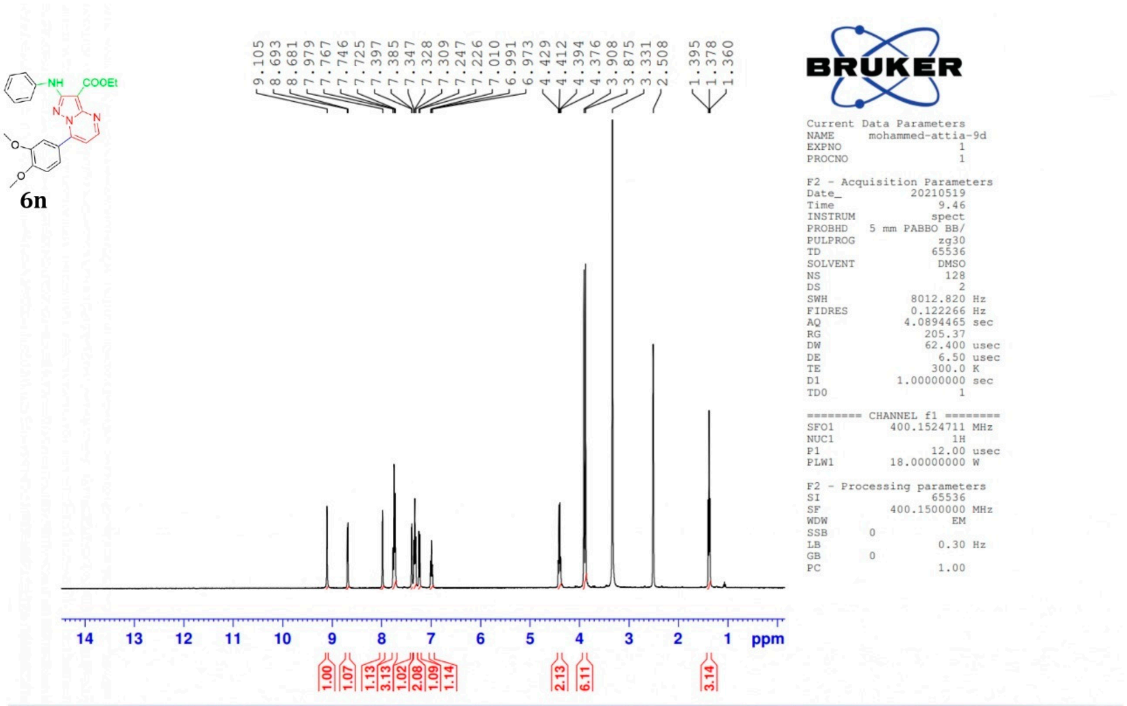

Figure S24. ^{1}H NMR of compound **6n**

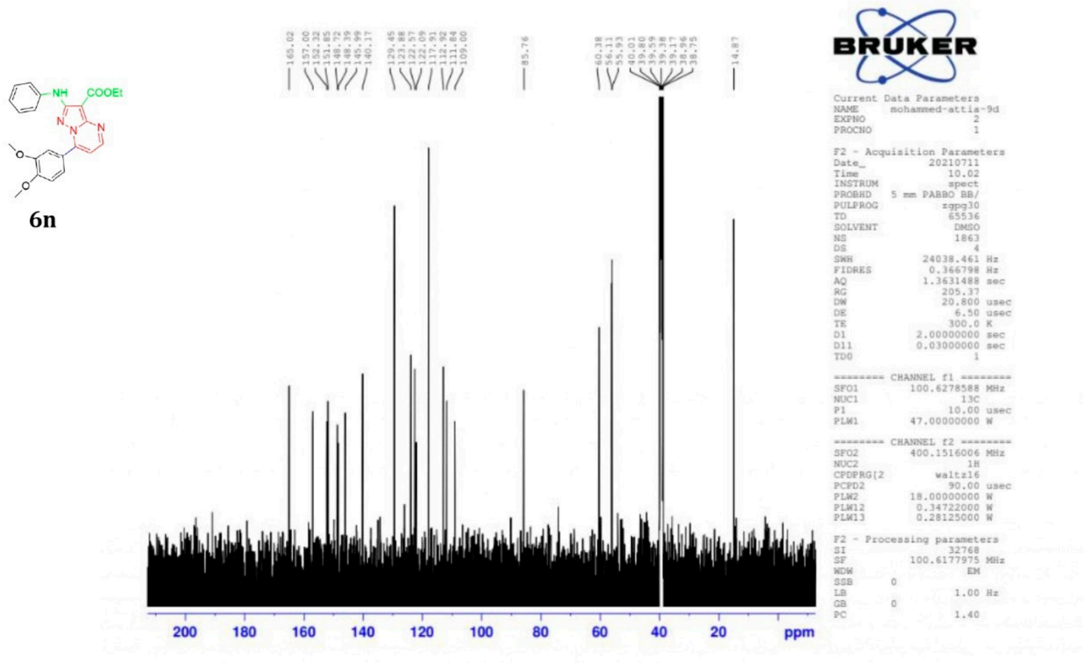

Figure S25. ^{13}C NMR of compound **6n**

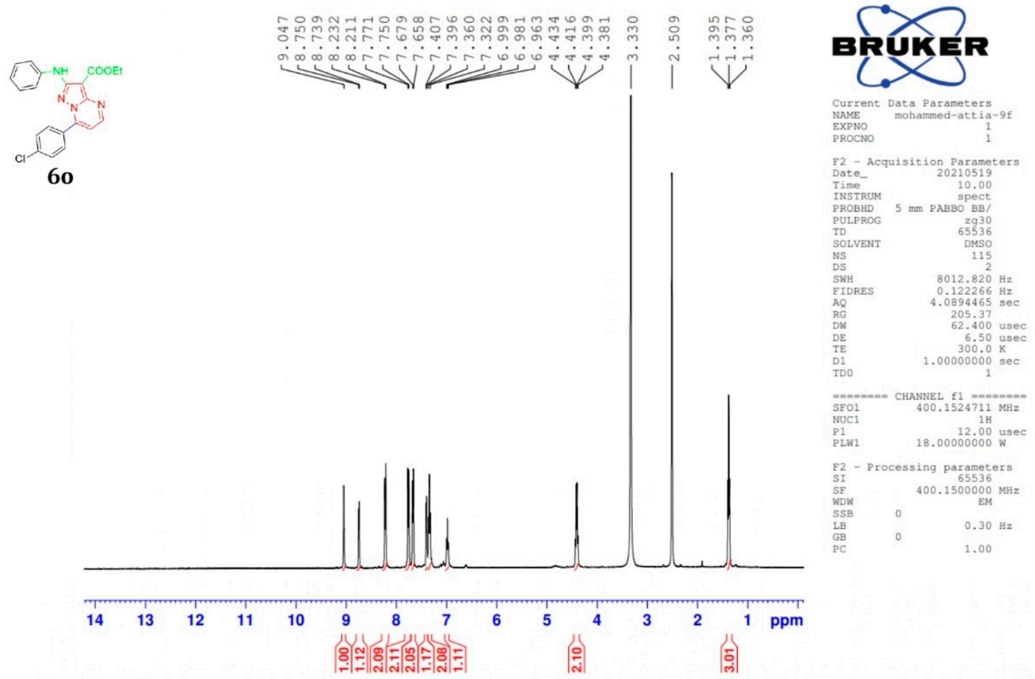

Figure S26. ^{1}H NMR of compound **6o**

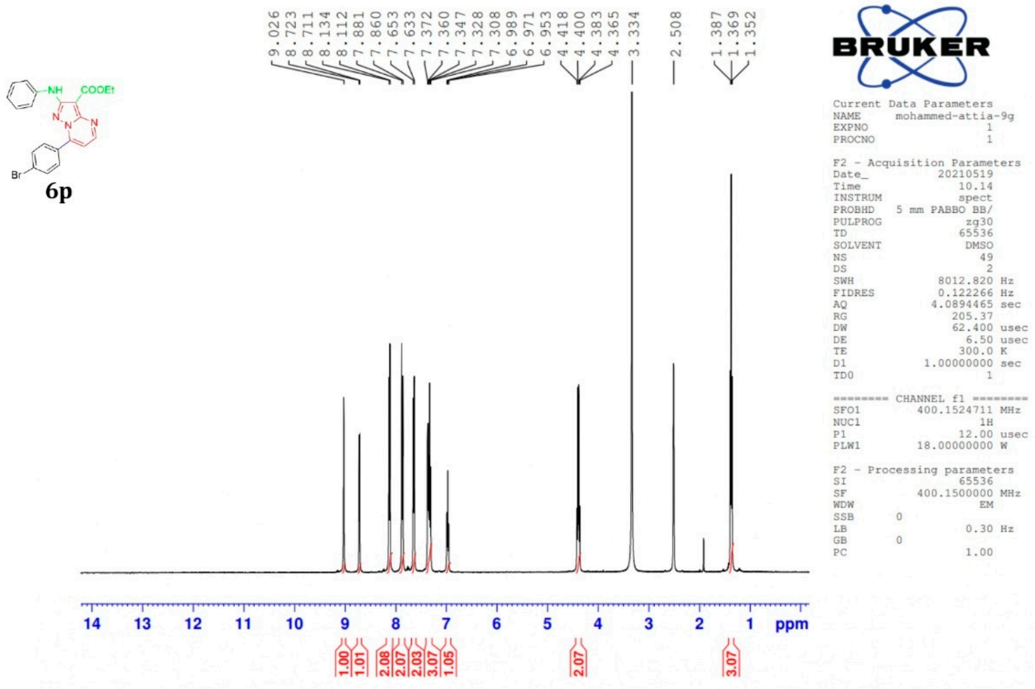

Figure S27. ^{1}H NMR of compound **6p**

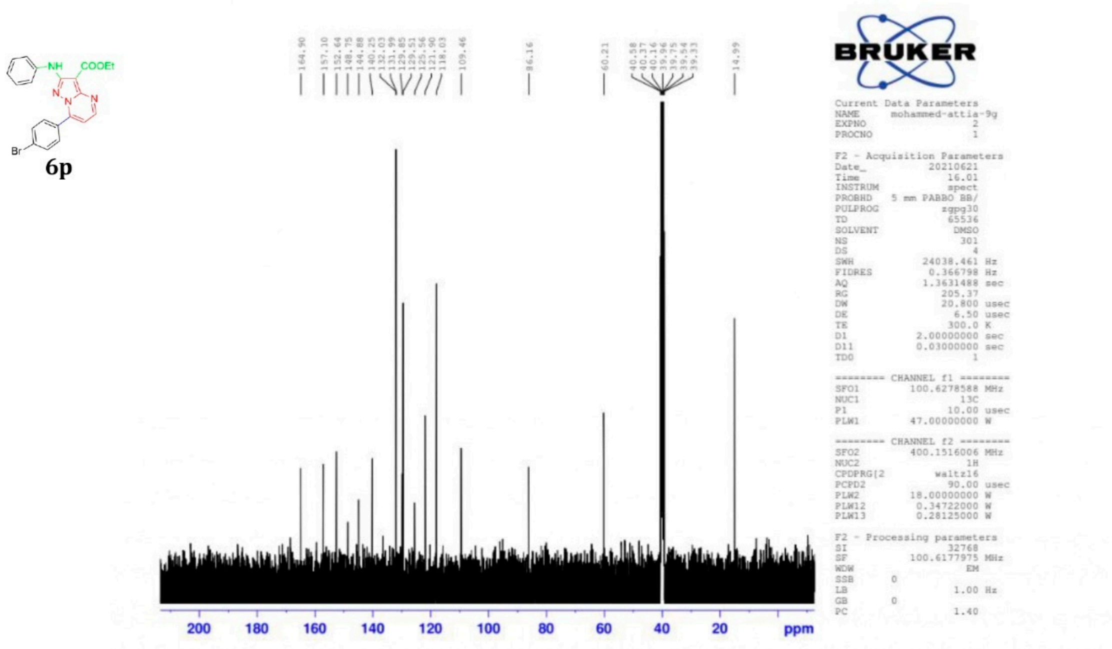

Figure S28. ^{13}C NMR of compound **6p**

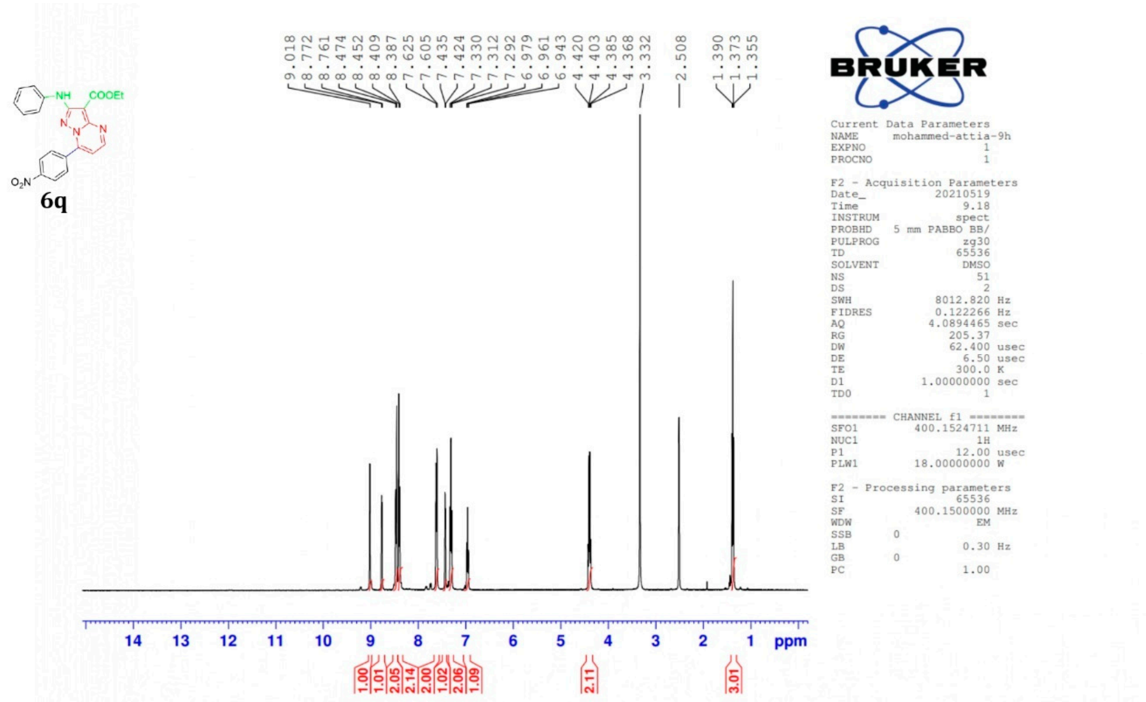

Figure S29. ^{1}H NMR of compound **6q**

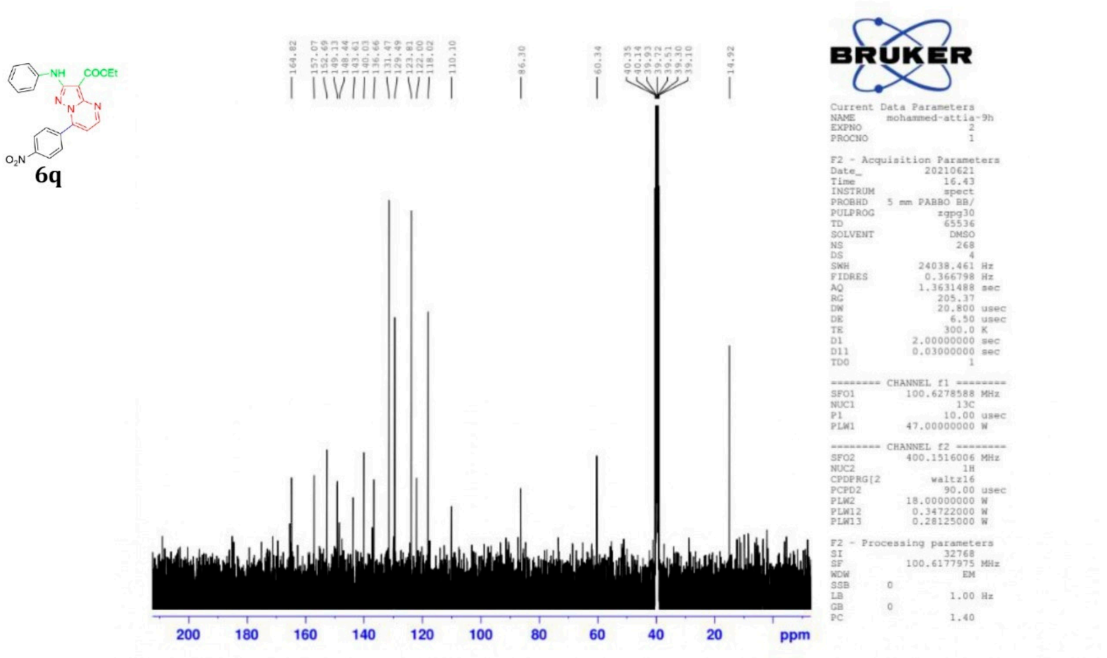

Figure S30. ^{13}C NMR of compound **6q**

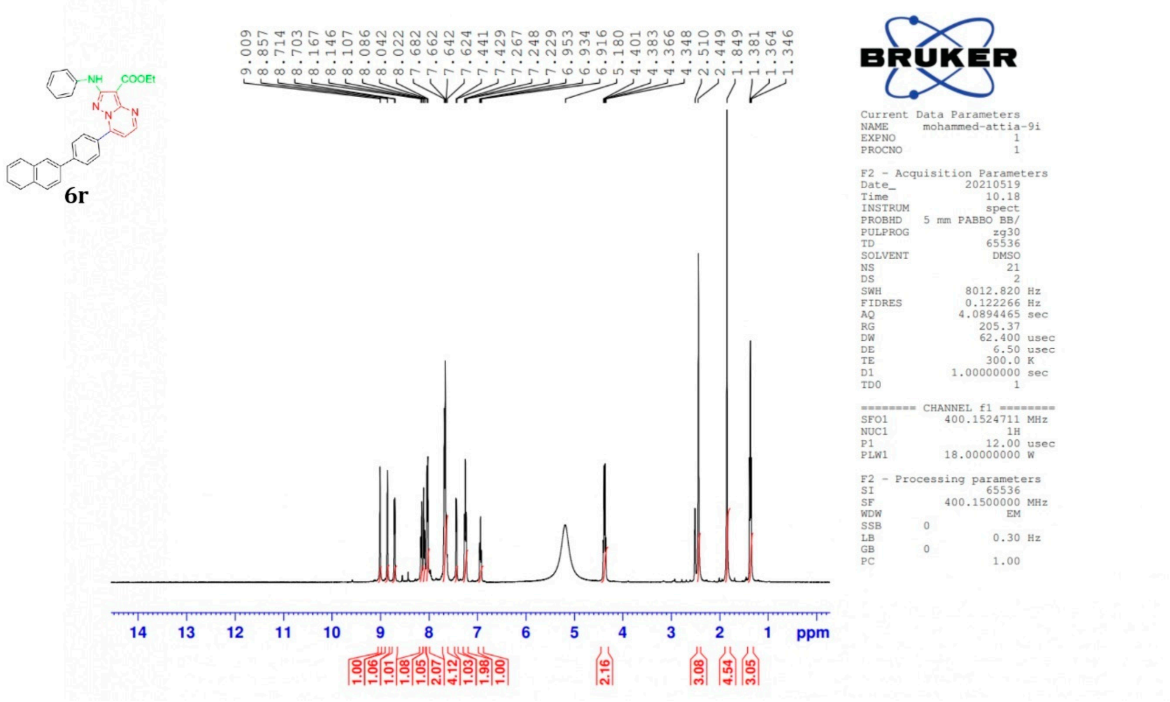

Figure S31. ^{1}H NMR of compound **6r**

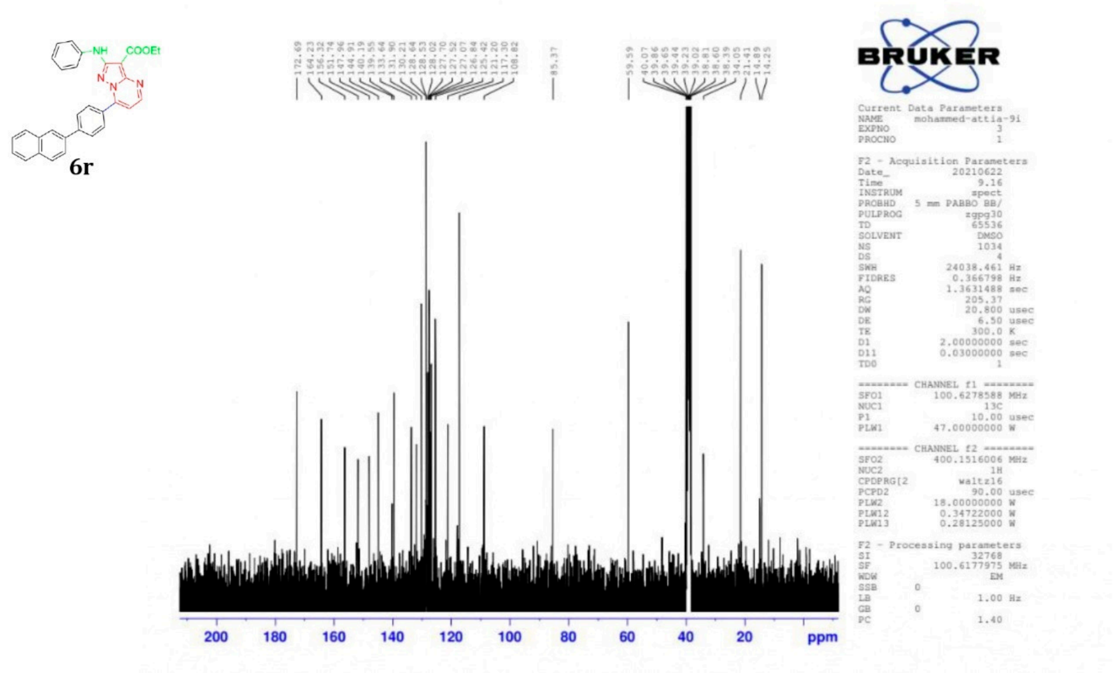

Figure S32. ^{13}C NMR of compound **6r**

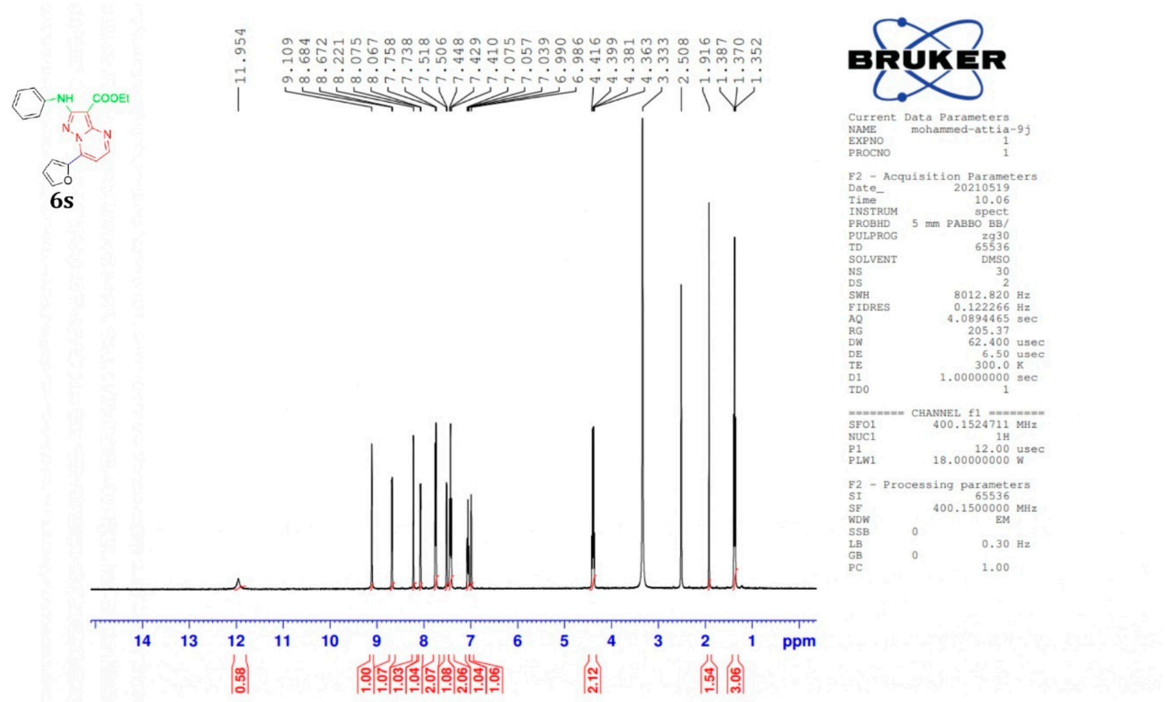

Figure S33. ^{1}H NMR of compound **6s**

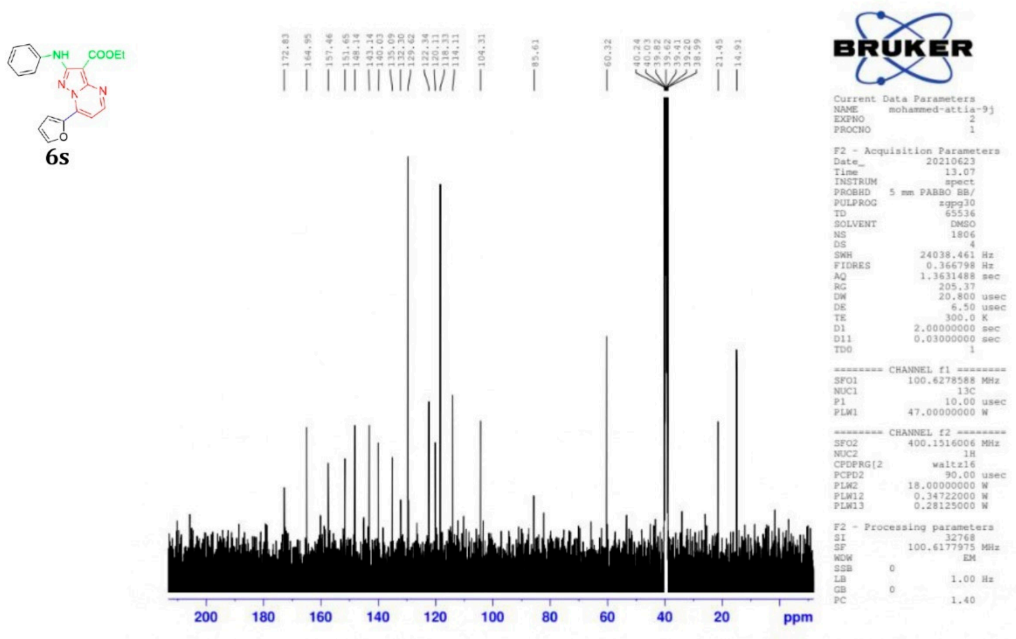

Figure S34. ^{13}C NMR of compound **6s**

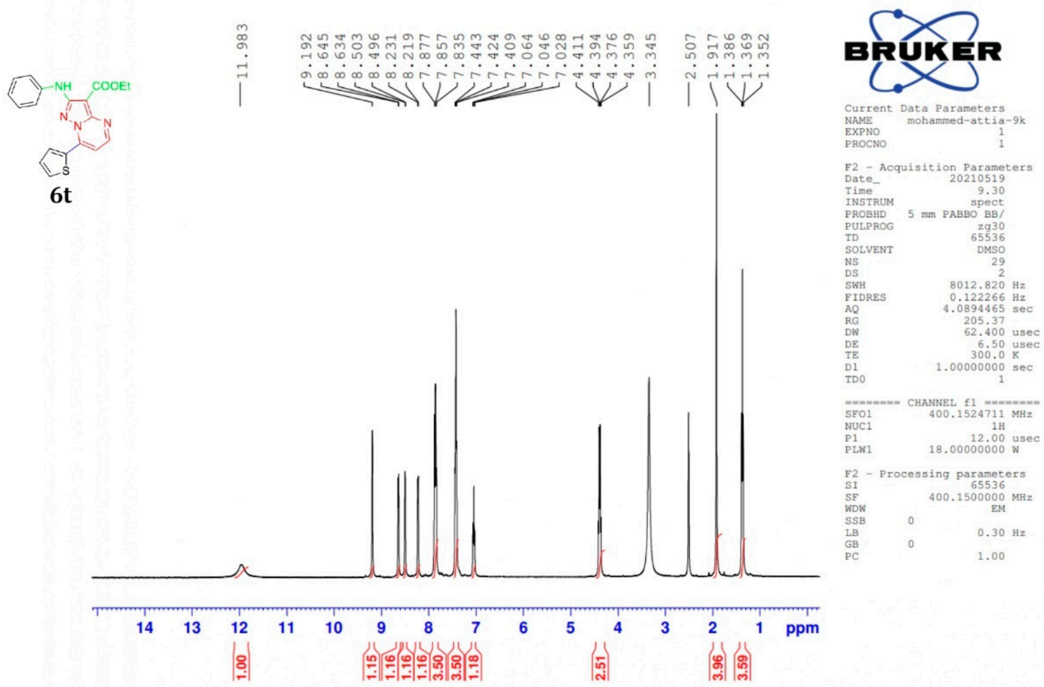

Figure S35. ^{1}H NMR of compound **6t**

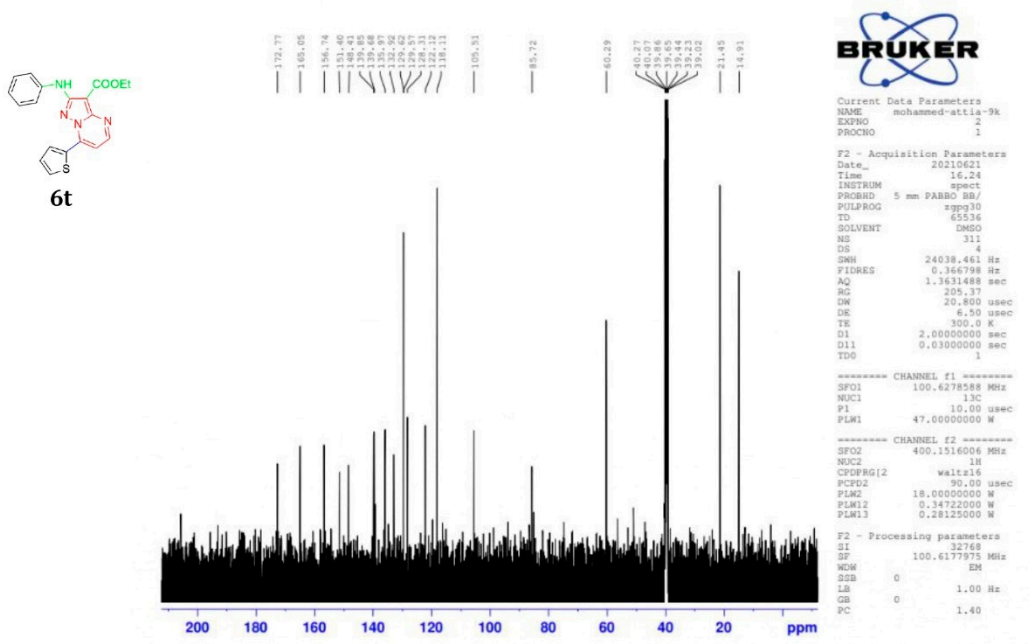

Figure S36. ^{13}C NMR of compound **6t**

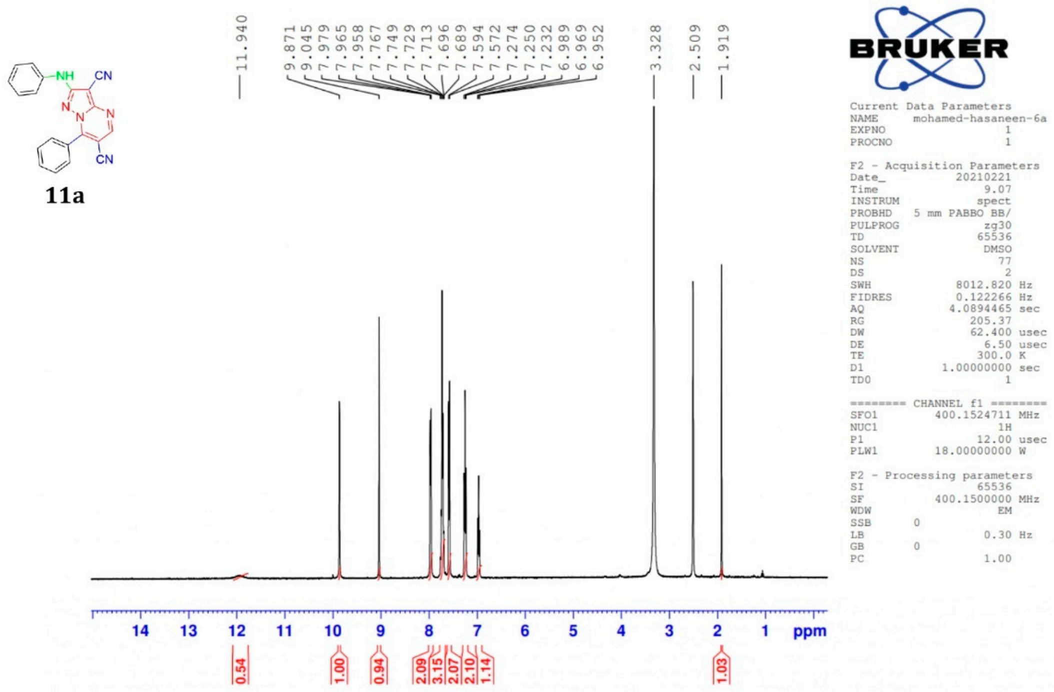

Figure S37. ^{1}H NMR of compound **11a**

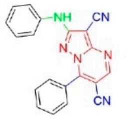

11a

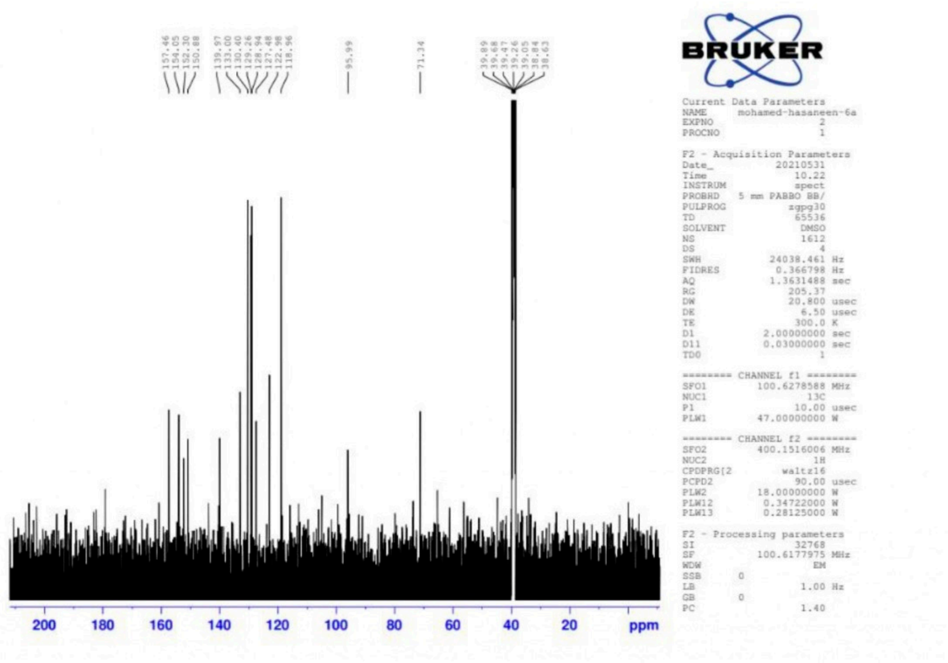

Figure S38. ^{13}C NMR of compound **11a**

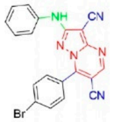

11b

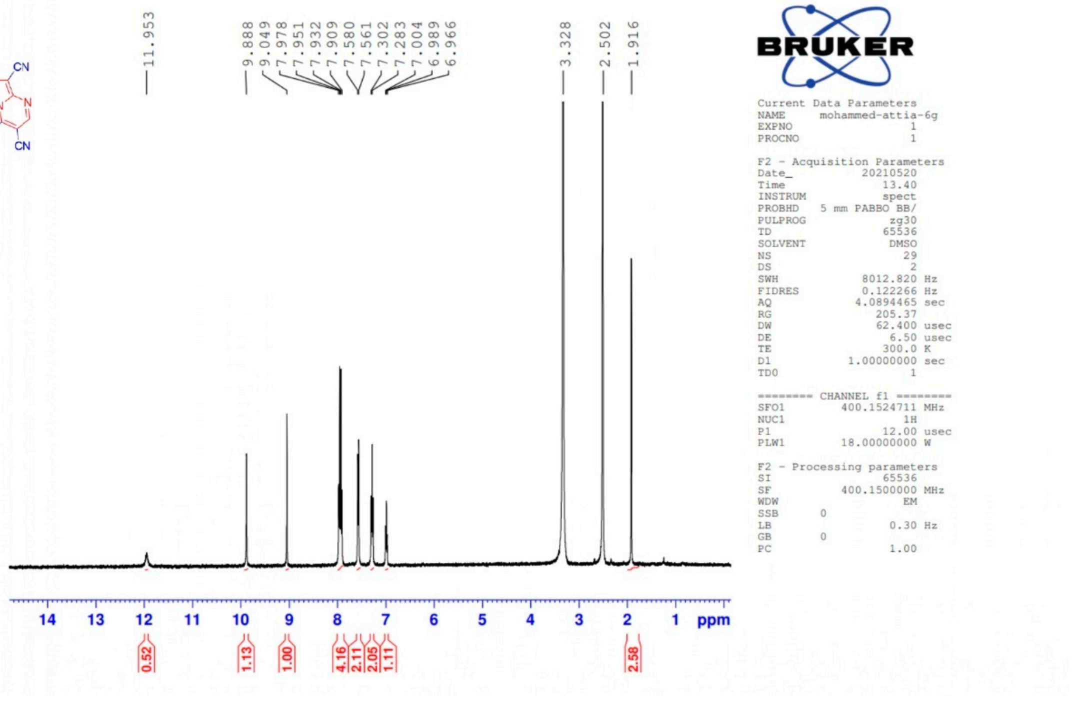

Figure S39. ^{1}H NMR of compound **11b**

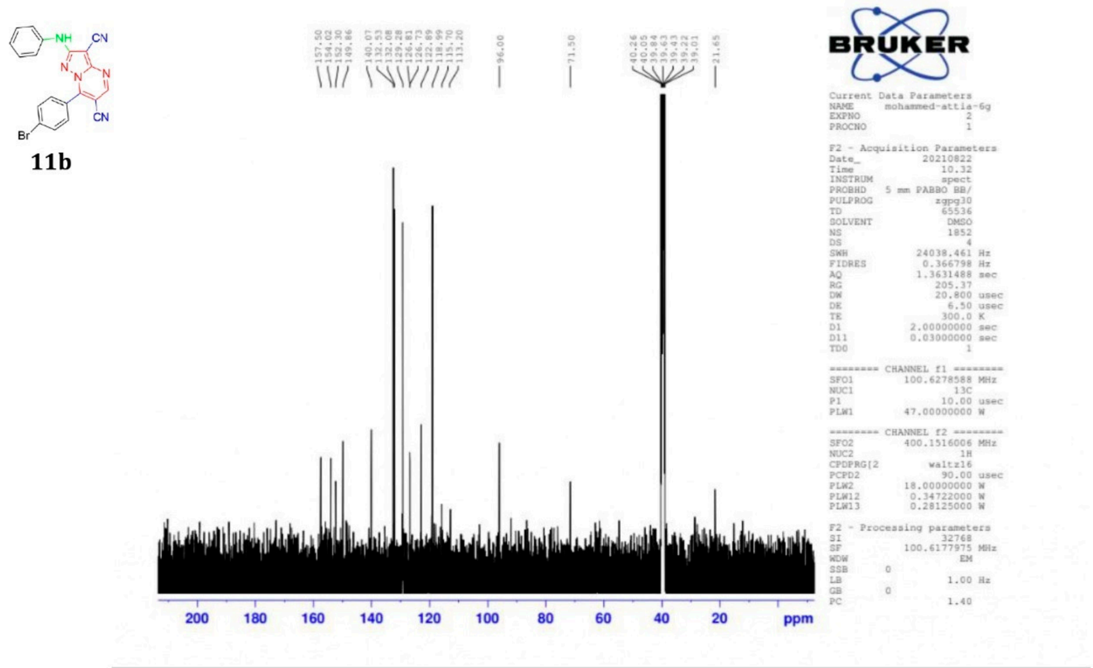

Figure S40. ^{13}C NMR of compound **11b**

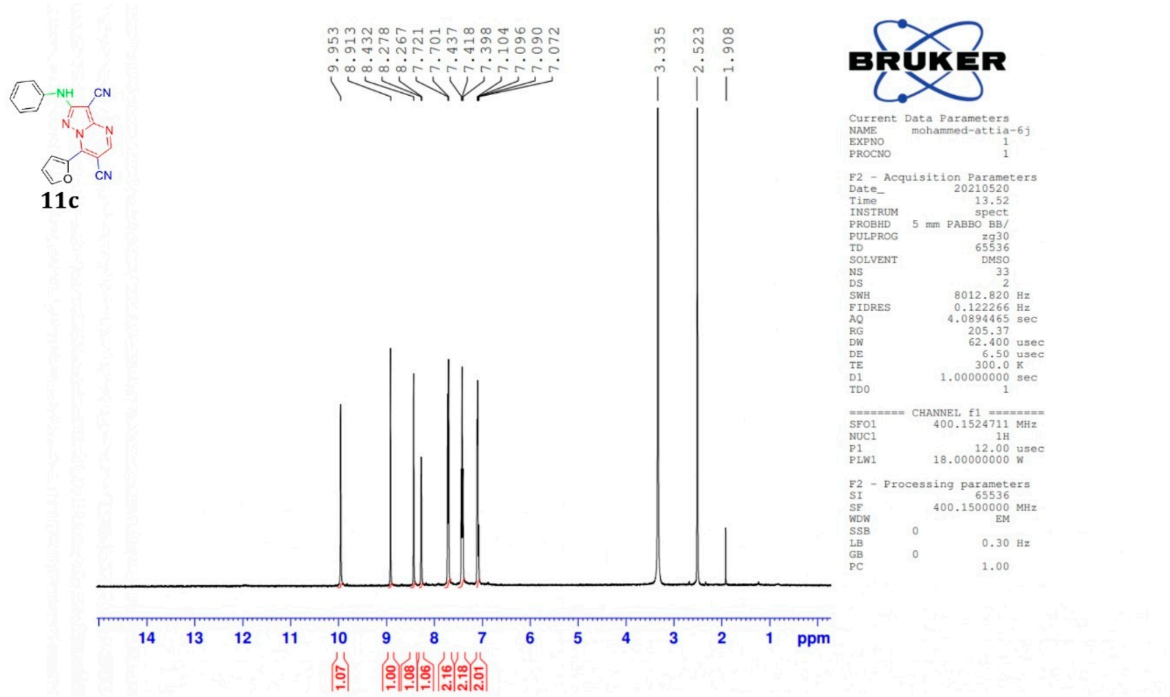

Figure S41. ^{1}H NMR of compound **11c**

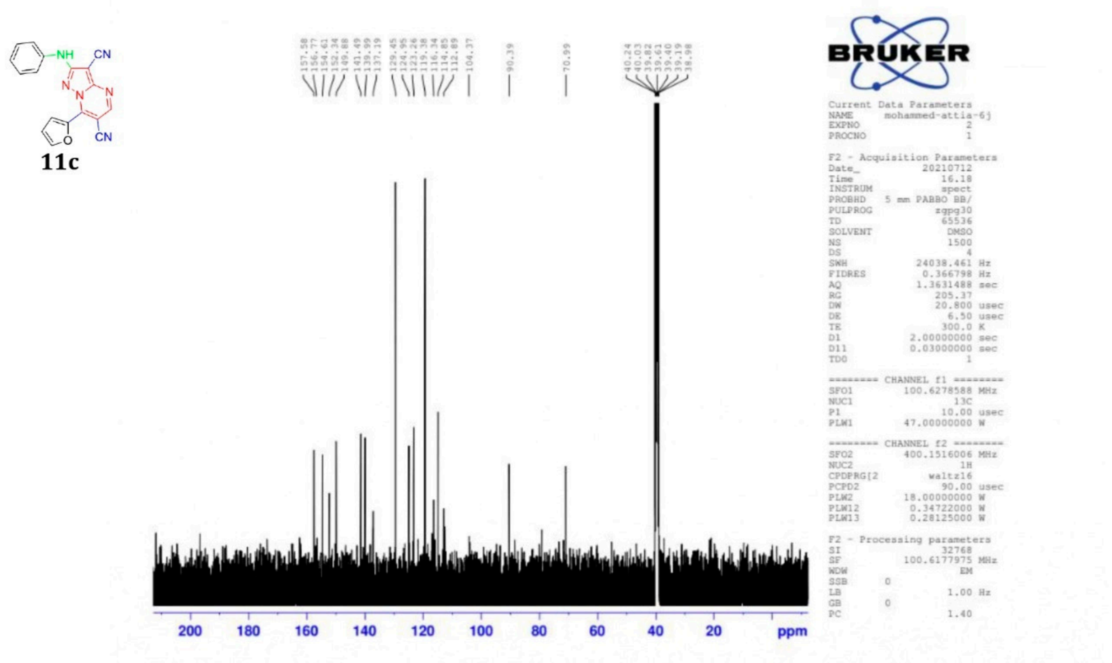

Figure S42. ^{13}C NMR of compound **11c**

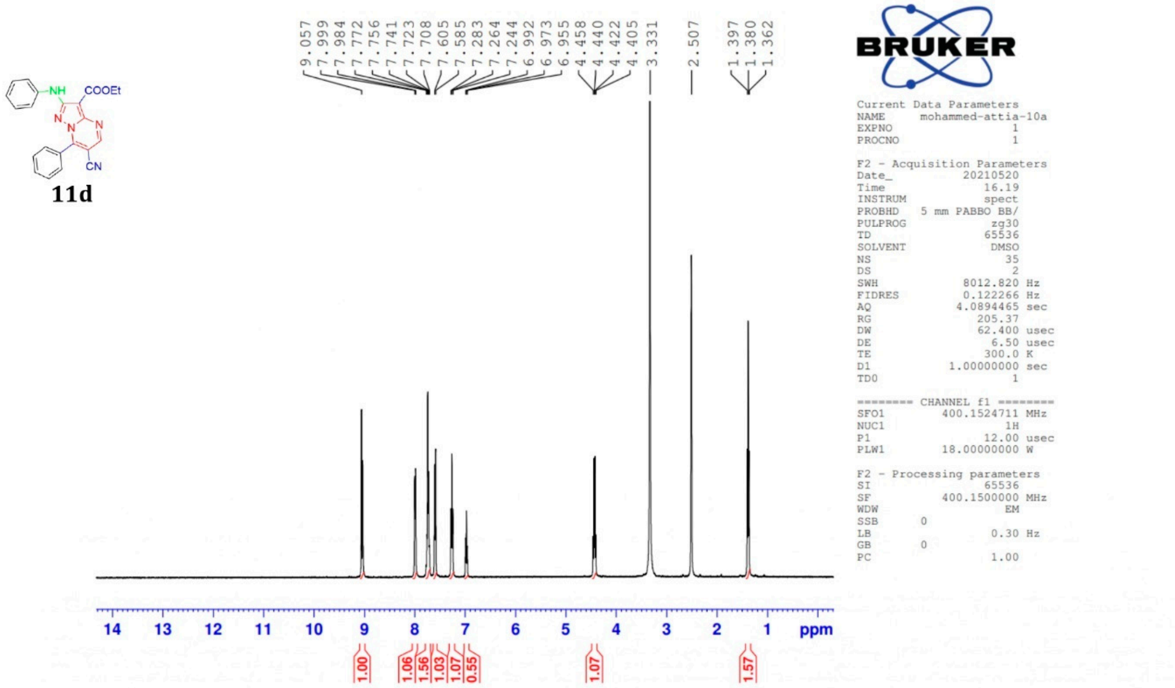

Figure S43. ^{1}H NMR of compound **11d**

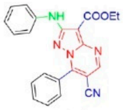

11d

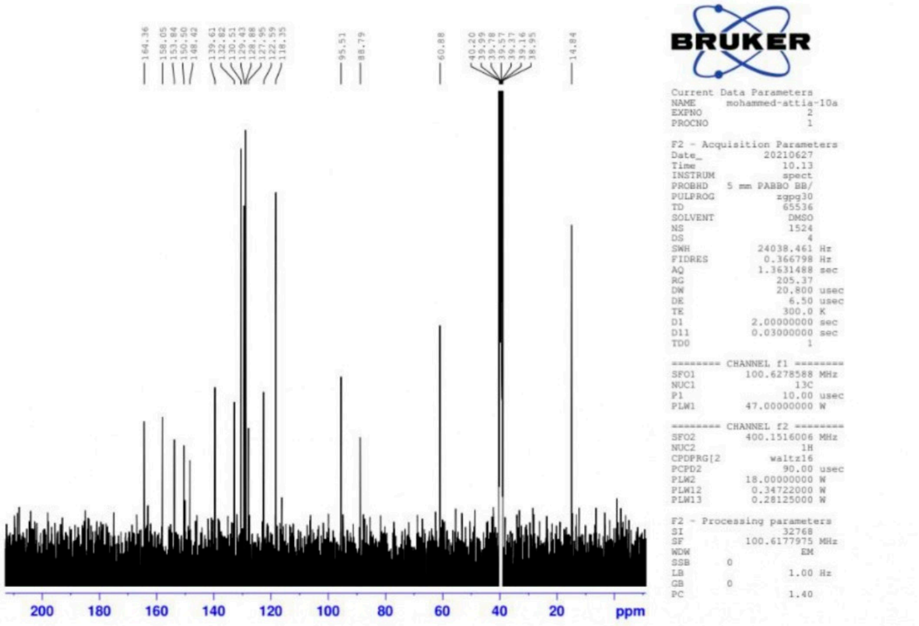

Figure S44. ^{13}C NMR of compound **11d**

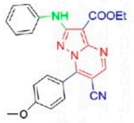

11e

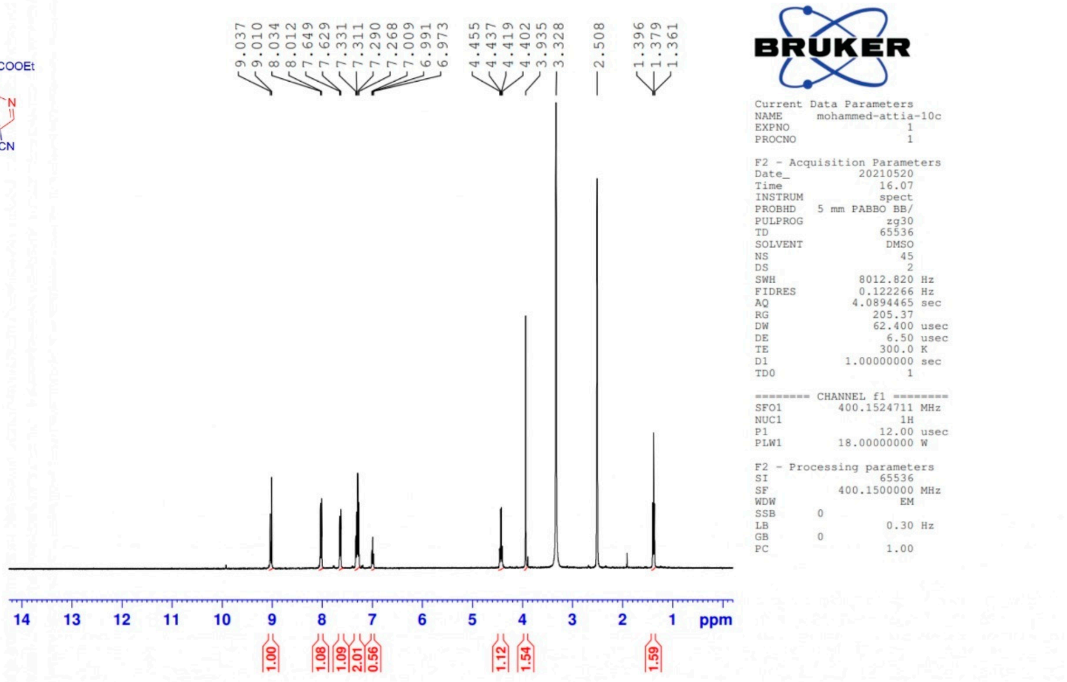

Figure S45. ^{1}H NMR of compound **11e**

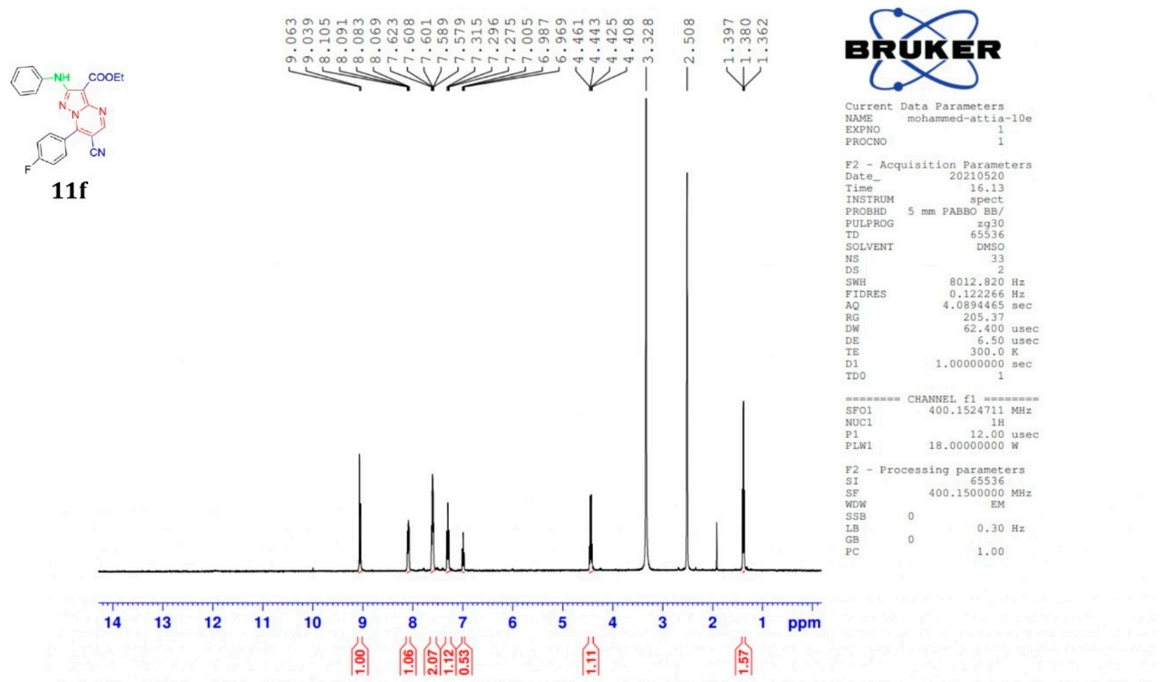

Figure S46. ^{1}H NMR of compound **11f**

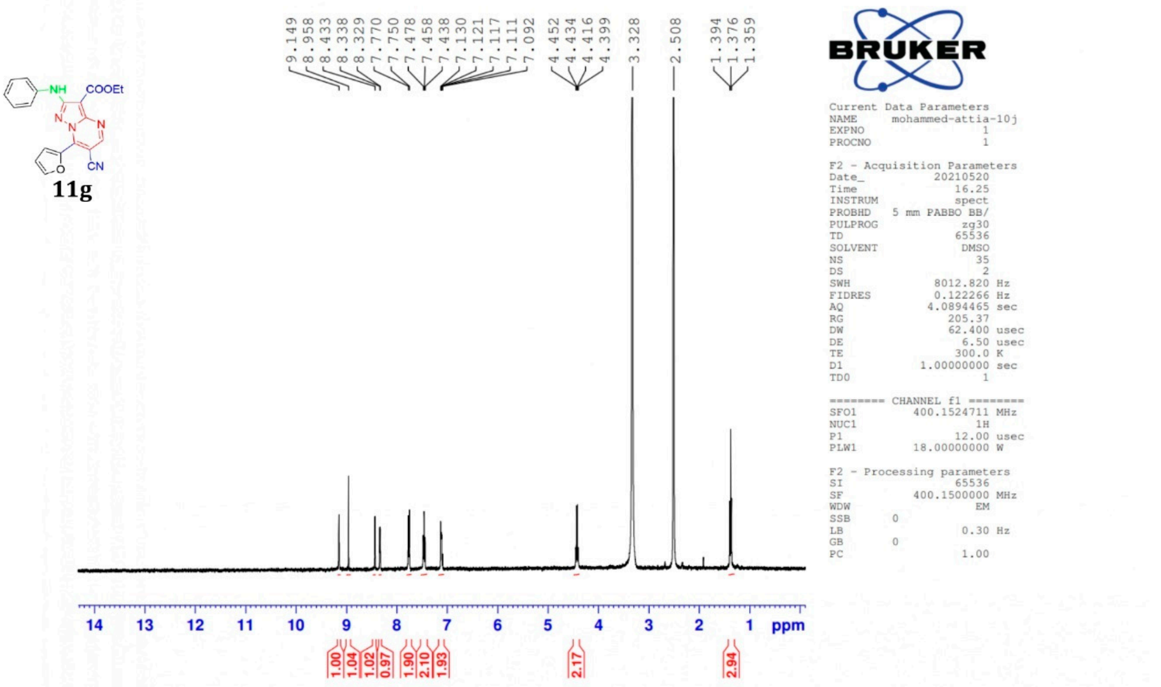

Figure S47. ^{1}H NMR of compound **11g**

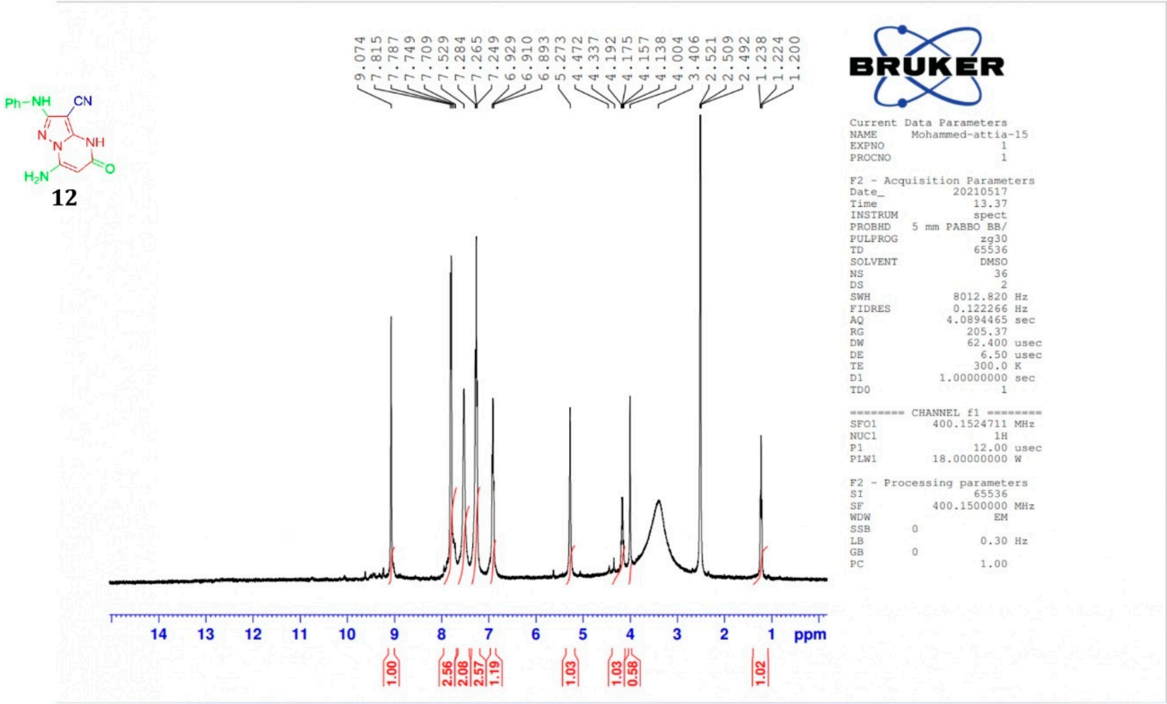

Figure S48. ^{1}H NMR of compound **12**

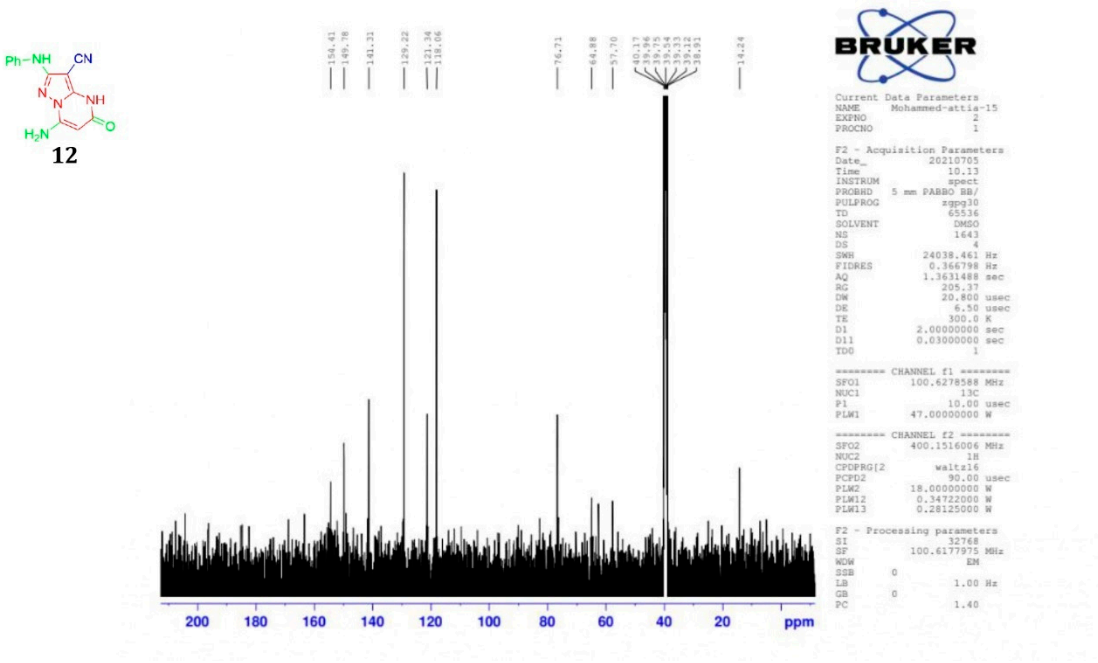

Figure S49. ^{13}C NMR of compound **12**

NCI panel cell line Mean graphs

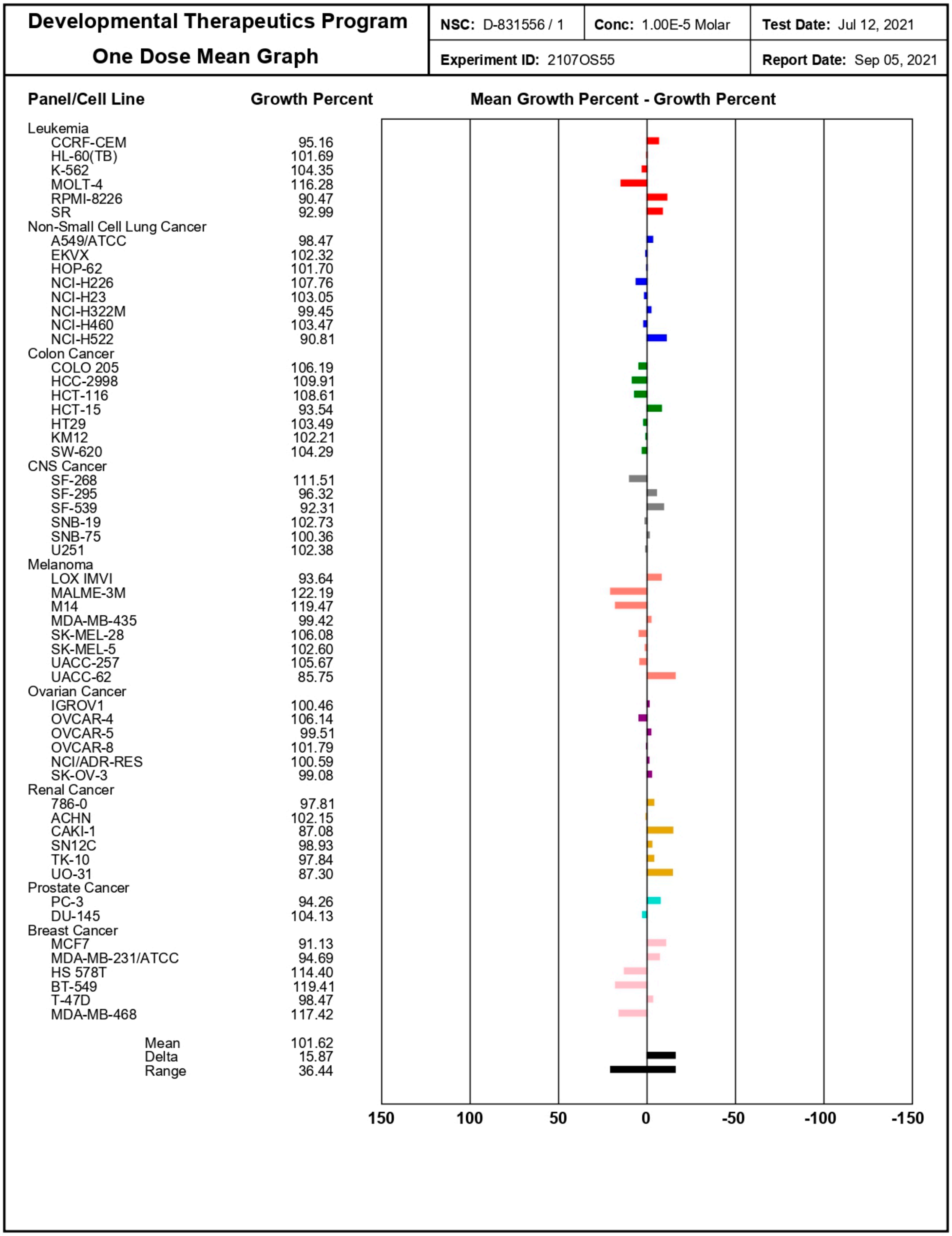

Figure S50. Mean graph of compound (**6a**) with colour codes for each cell line.

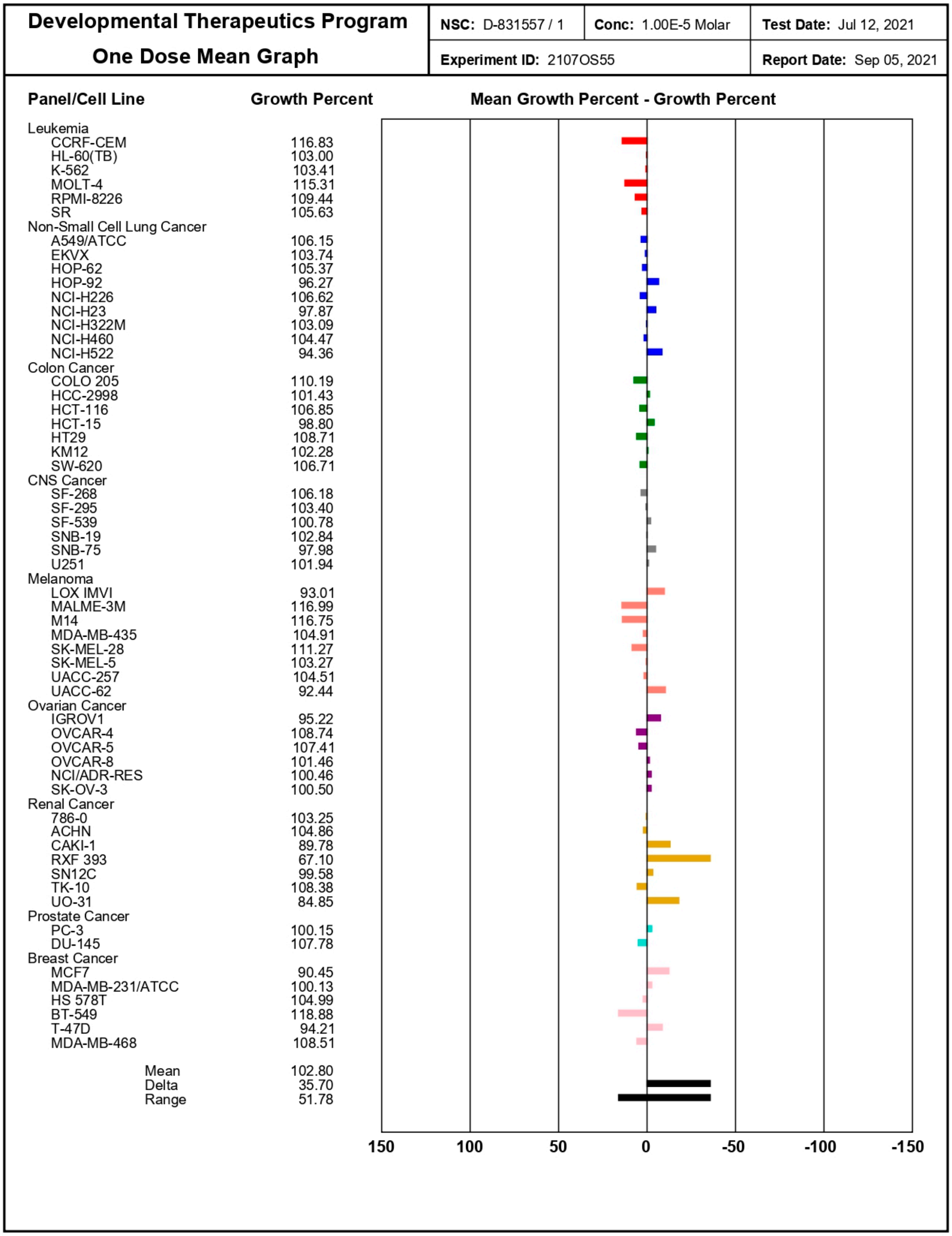

Figure S51. Mean graph of compound (**6b**) with colour codes for each cell line.

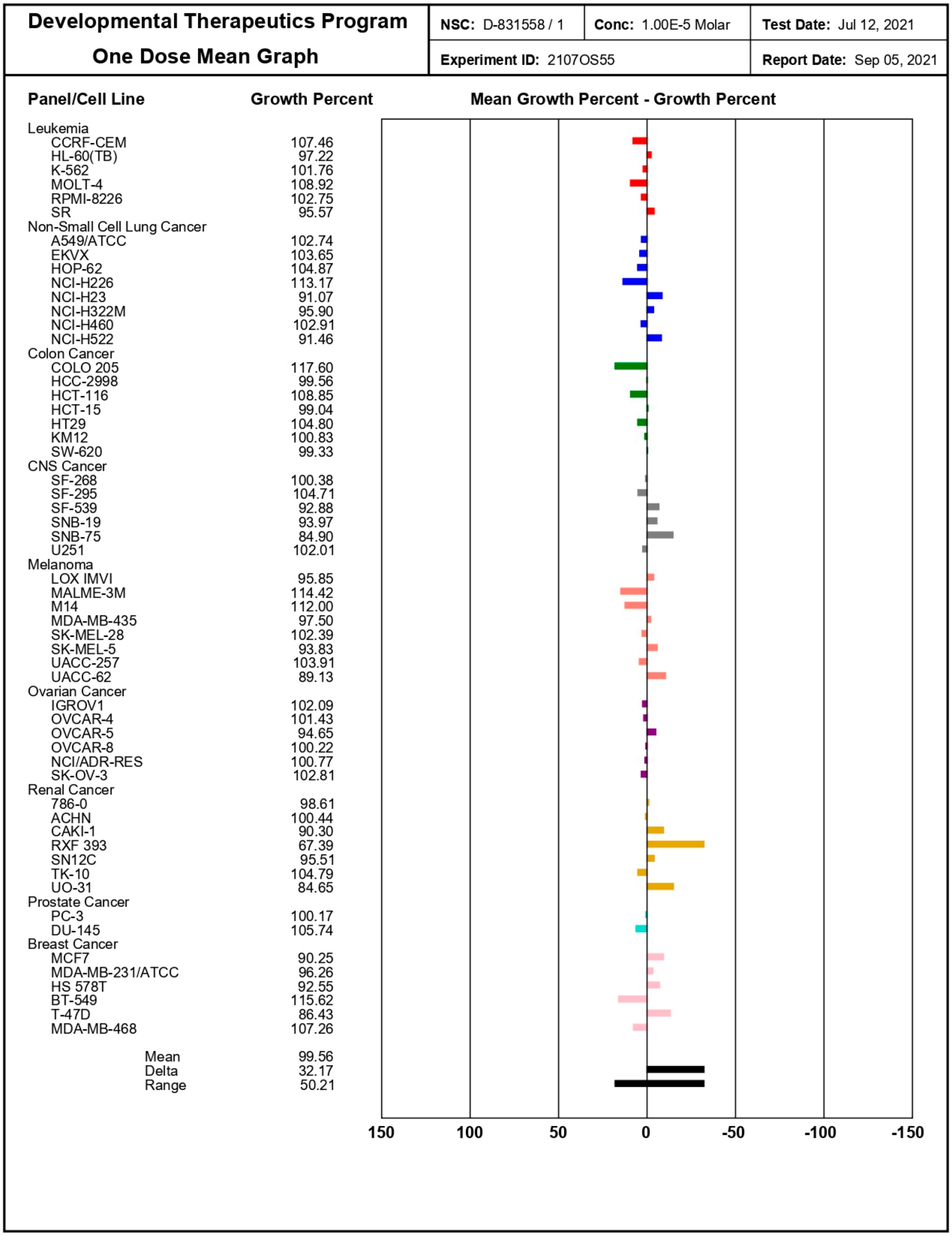

Figure S52. Mean graph of compound (**6c**) with colour codes for each cell line.

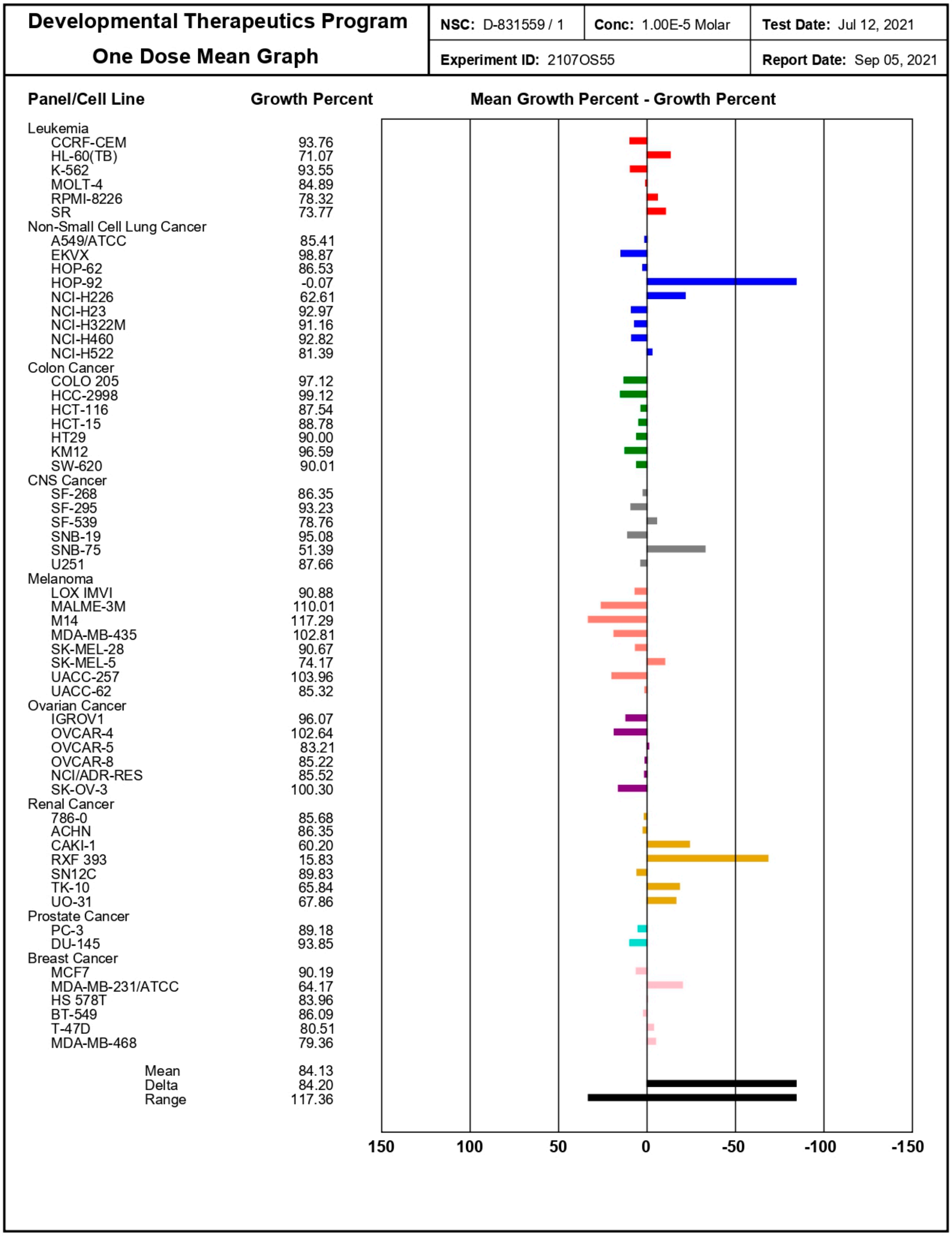

Figure S53. Mean graph of compound (**6d**) with colour codes for each cell line.

Developmental Therapeutics Program One Dose Mean Graph	NSC: D-831560 / 1	Conc: 1.00E-5 Molar	Test Date: Jul 12, 2021
	Experiment ID: 2107OS55		Report Date: Sep 05, 2021

Panel/Cell Line	Growth Percent
**Leukemia**	
CCRF-CEM	104.36
HL-60(TB)	92.07
K-562	104.38
MOLT-4	105.64
RPMI-8226	101.41
SR	96.27
**Non-Small Cell Lung Cancer**	
A549/ATCC	94.67
EKVX	102.14
HOP-62	114.74
HOP-92	82.03
NCI-H226	98.70
NCI-H23	104.47
NCI-H322M	99.95
NCI-H460	102.97
NCI-H522	88.79
**Colon Cancer**	
COLO 205	121.78
HCC-2998	106.93
HCT-116	95.10
HCT-15	101.22
HT29	100.49
KM12	101.40
SW-620	102.40
**CNS Cancer**	
SF-268	100.22
SF-295	99.23
SF-539	99.15
SNB-19	99.78
SNB-75	110.97
U251	99.13
**Melanoma**	
LOX IMVI	95.57
MALME-3M	105.96
M14	99.61
MDA-MB-435	103.47
SK-MEL-28	102.37
SK-MEL-5	79.08
UACC-257	100.08
UACC-62	88.70
**Ovarian Cancer**	
IGROV1	105.32
OVCAR-4	101.45
OVCAR-5	101.93
OVCAR-8	100.94
NCI/ADR-RES	104.80
SK-OV-3	108.57
**Renal Cancer**	
786-0	94.98
ACHN	103.53
CAKI-1	94.85
RXF 393	65.74
SN12C	102.88
TK-10	99.44
UO-31	85.47
**Prostate Cancer**	
PC-3	104.00
DU-145	104.78
**Breast Cancer**	
MCF7	93.57
MDA-MB-231/ATCC	103.23
HS 578T	103.23
BT-549	95.06
T-47D	110.00
MDA-MB-468	95.70
Mean	99.73
Delta	33.99
Range	56.04

Mean Growth Percent - Growth Percent

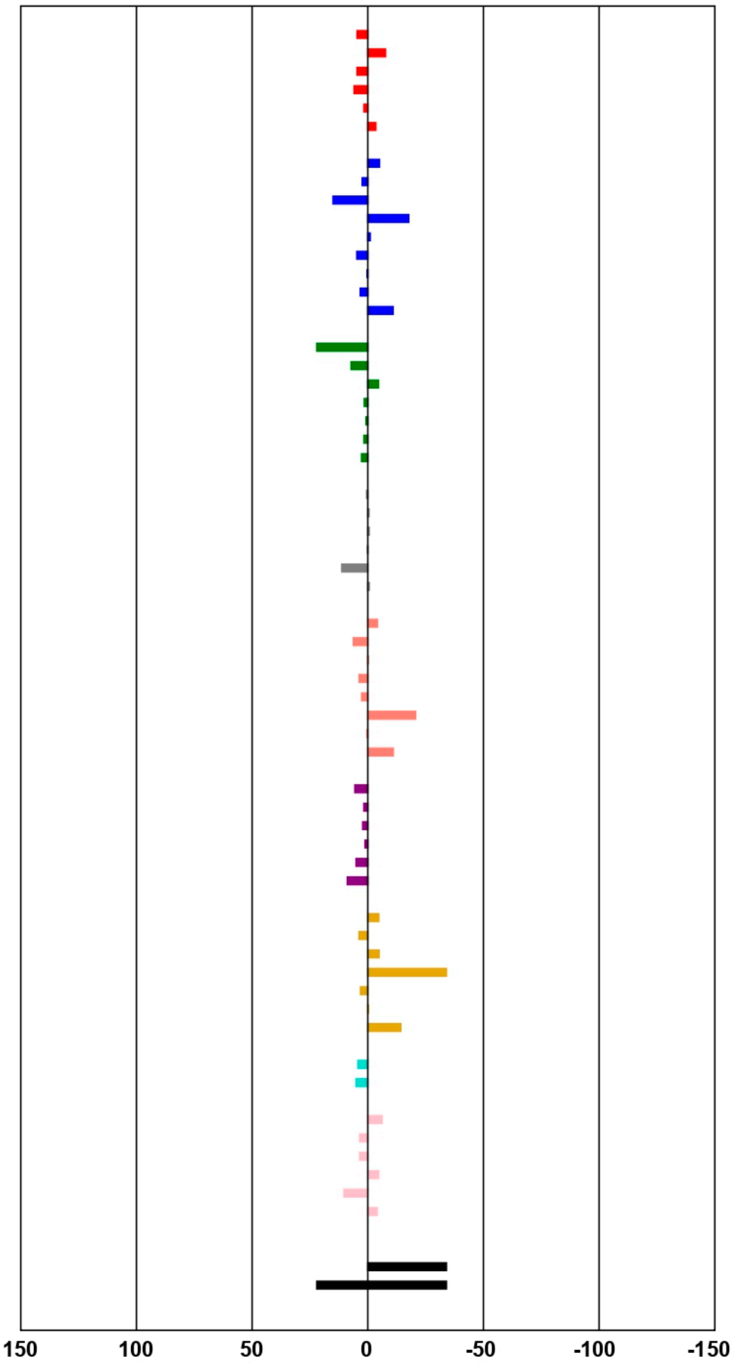

Figure S54. Mean graph of compound (**6e**) with colour codes for each cell line.

Developmental Therapeutics Program One Dose Mean Graph	NSC: D-831561 / 1	Conc: 1.00E-5 Molar	Test Date: Jul 12, 2021
	Experiment ID: 2107OS55		Report Date: Sep 05, 2021

Panel/Cell Line	Growth Percent
**Leukemia**	
CCRF-CEM	97.83
HL-60(TB)	90.83
K-562	110.51
MOLT-4	100.04
RPMI-8226	89.05
SR	53.22
**Non-Small Cell Lung Cancer**	
A549/ATCC	95.81
EKVX	97.24
HOP-62	104.65
HOP-92	73.29
NCI-H226	105.50
NCI-H23	96.97
NCI-H322M	99.56
NCI-H460	99.28
NCI-H522	88.63
**Colon Cancer**	
COLO 205	104.37
HCC-2998	103.81
HCT-116	99.29
HCT-15	95.21
HT29	100.31
KM12	100.83
SW-620	97.60
**CNS Cancer**	
SF-268	101.10
SF-295	101.66
SF-539	95.94
SNB-19	101.98
SNB-75	108.76
U251	99.37
**Melanoma**	
LOX IMVI	97.06
MALME-3M	114.34
M14	104.74
MDA-MB-435	100.69
SK-MEL-28	101.49
SK-MEL-5	96.72
UACC-257	101.81
UACC-62	87.24
**Ovarian Cancer**	
IGROV1	98.48
OVCAR-4	99.48
OVCAR-5	103.91
OVCAR-8	100.06
NCI/ADR-RES	99.23
SK-OV-3	102.53
**Renal Cancer**	
786-0	97.67
ACHN	105.46
CAKI-1	93.36
RXF 393	65.25
SN12C	104.05
TK-10	109.79
UO-31	81.87
**Prostate Cancer**	
PC-3	98.42
DU-145	102.52
**Breast Cancer**	
MCF7	93.25
MDA-MB-231/ATCC	109.09
HS 578T	103.71
BT-549	116.06
T-47D	104.43
MDA-MB-468	97.27
Mean	98.29
Delta	45.07
Range	62.84

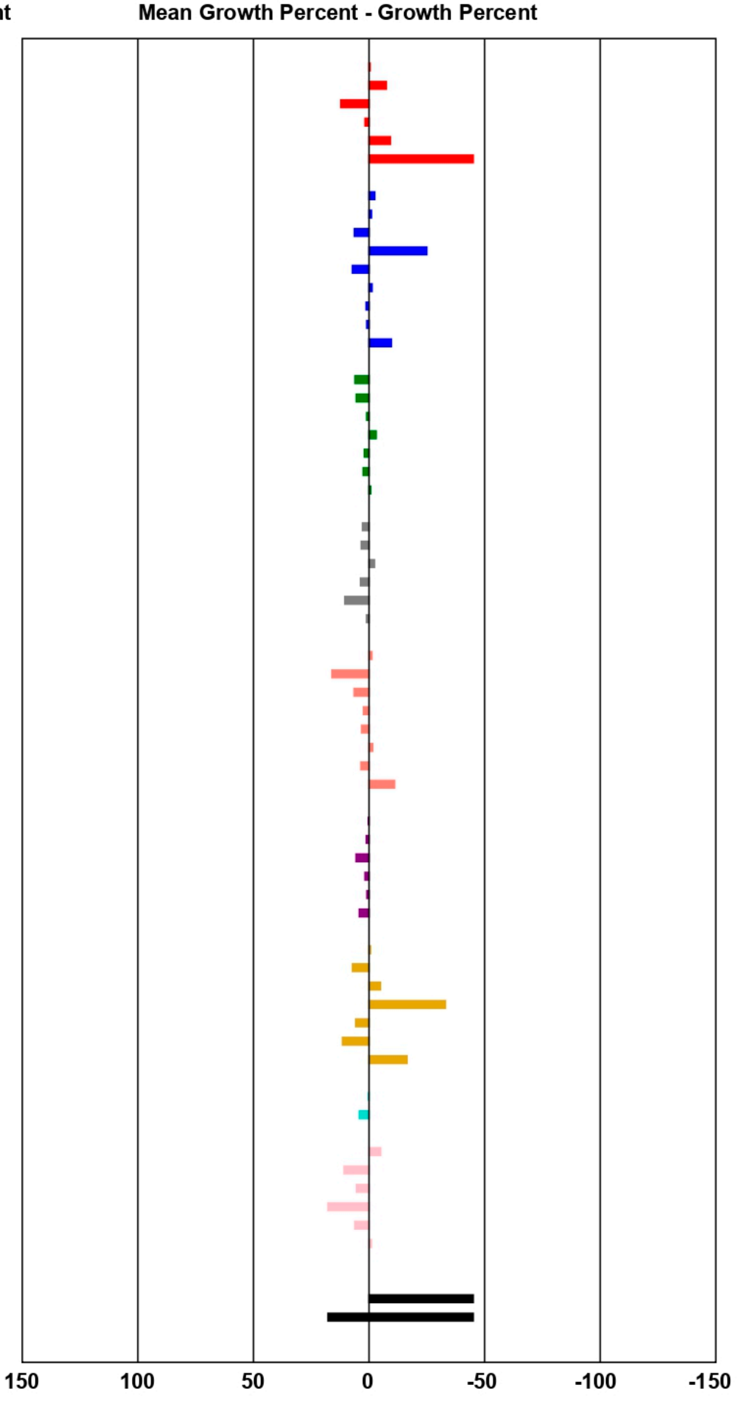

Figure S55. Mean graph of compound (**6f**) with colour codes for each cell line.

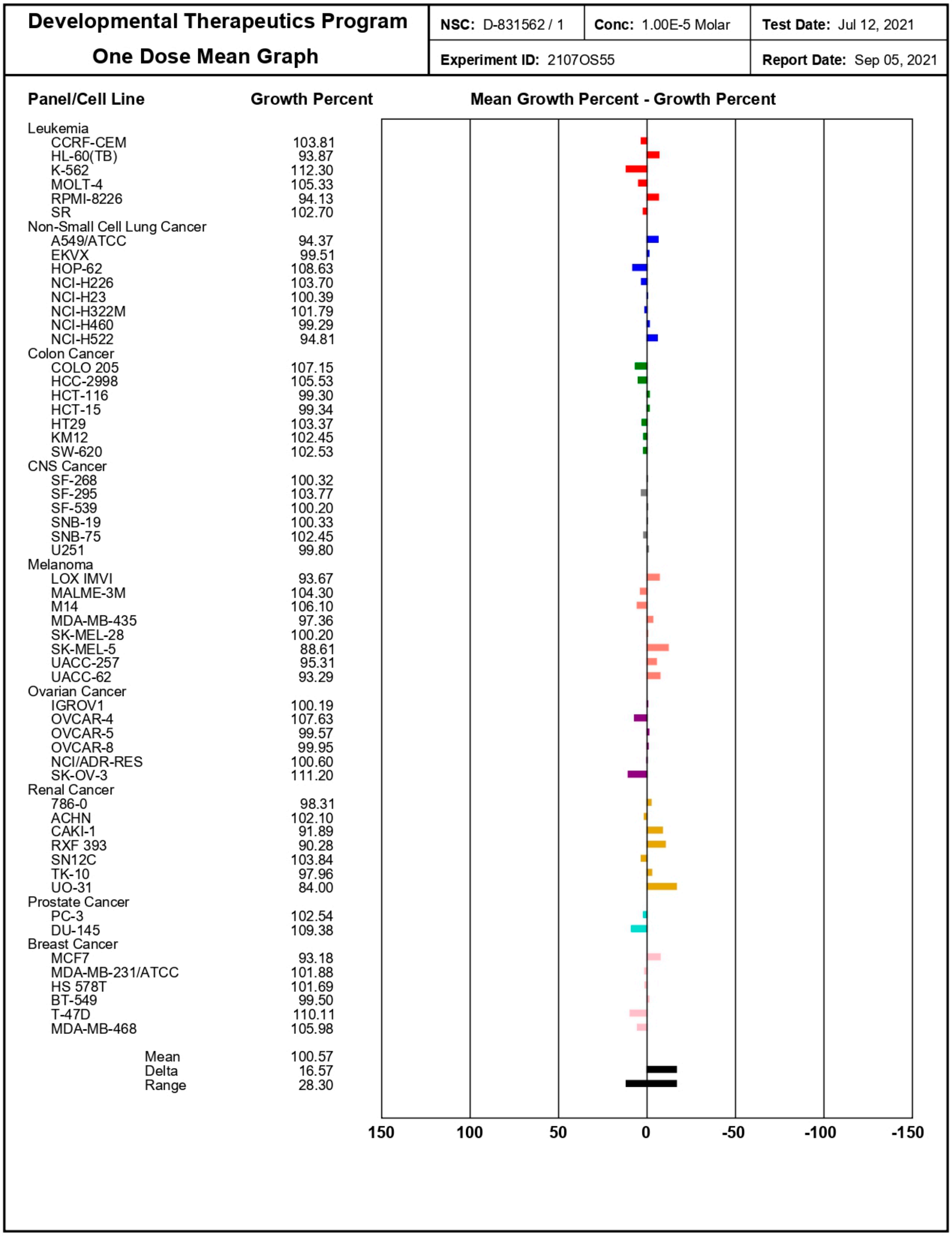

Figure S56. Mean graph of compound (**6g**) with colour codes for each cell line.

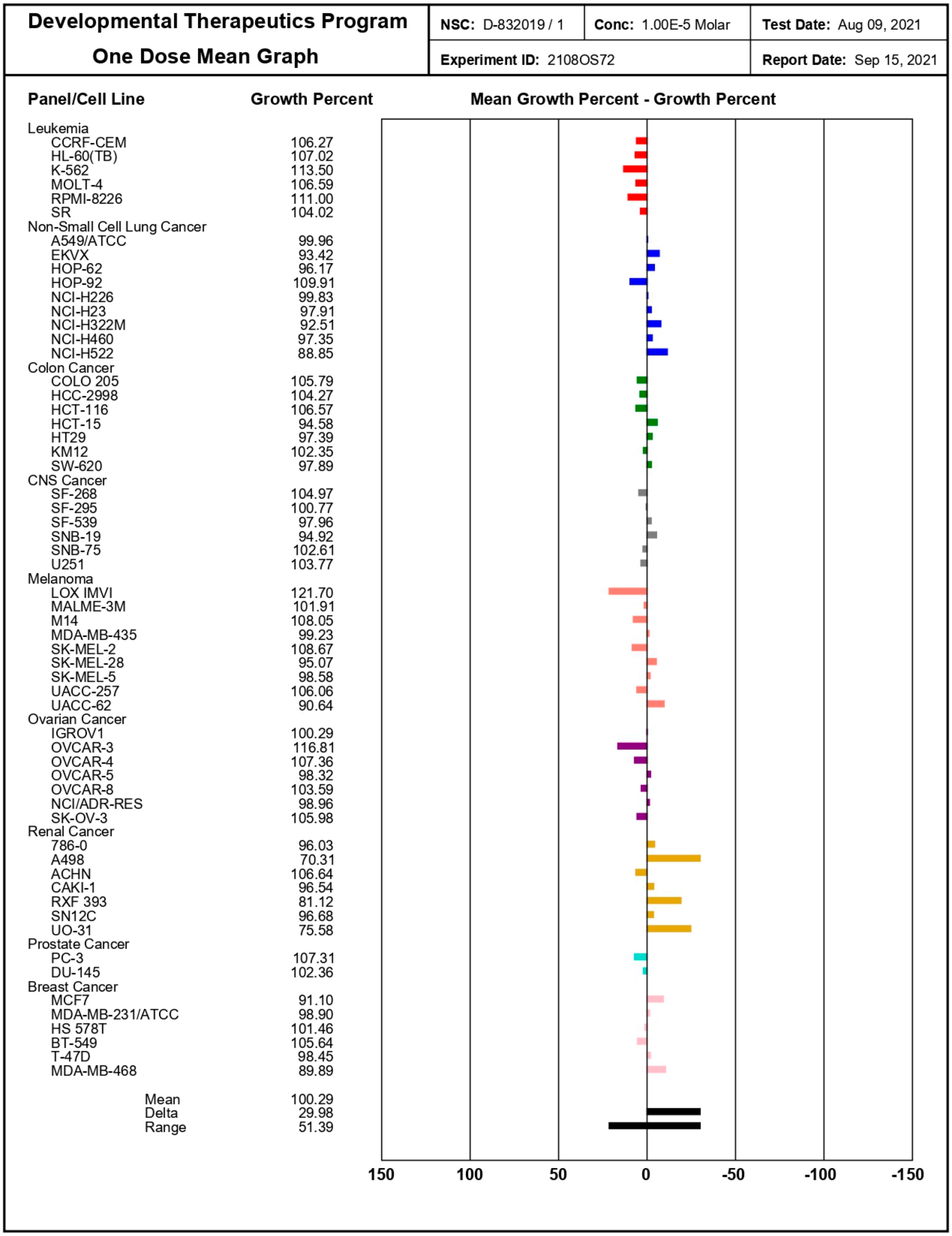

Figure S57. Mean graph of compound (**6h**) with colour codes for each cell line.

Developmental Therapeutics Program One Dose Mean Graph	NSC: D-831563 / 1	Conc: 1.00E-5 Molar	Test Date: Jul 12, 2021
	Experiment ID: 2107OS55		Report Date: Sep 05, 2021

Panel/Cell Line	Growth Percent
**Leukemia**	
CCRF-CEM	98.40
HL-60(TB)	84.21
K-562	103.35
MOLT-4	101.44
RPMI-8226	94.64
SR	81.48
**Non-Small Cell Lung Cancer**	
A549/ATCC	89.61
EKVX	96.78
HOP-62	104.12
HOP-92	31.04
NCI-H226	86.01
NCI-H23	99.24
NCI-H322M	91.71
NCI-H460	91.77
NCI-H522	86.92
**Colon Cancer**	
COLO 205	98.76
HCC-2998	96.80
HCT-116	92.92
HCT-15	93.07
HT29	96.01
KM12	96.83
SW-620	94.21
**CNS Cancer**	
SF-268	103.81
SF-295	95.89
SF-539	95.85
SNB-19	95.09
SNB-75	98.26
U251	93.37
**Melanoma**	
LOX IMVI	95.82
MALME-3M	108.72
M14	112.24
MDA-MB-435	94.80
SK-MEL-28	98.74
SK-MEL-5	97.34
UACC-257	100.40
UACC-62	91.34
**Ovarian Cancer**	
IGROV1	97.60
OVCAR-4	102.31
OVCAR-5	98.97
OVCAR-8	95.56
NCI/ADR-RES	96.28
SK-OV-3	96.55
**Renal Cancer**	
786-0	89.79
ACHN	94.75
CAKI-1	80.23
RXF 393	55.47
SN12C	92.48
TK-10	93.93
UO-31	83.99
**Prostate Cancer**	
PC-3	97.41
DU-145	92.95
**Breast Cancer**	
MCF7	91.23
MDA-MB-231/ATCC	87.00
HS 578T	100.82
BT-549	73.30
T-47D	95.88
MDA-MB-468	91.05
Mean	92.96
Delta	61.92
Range	81.20

Mean Growth Percent - Growth Percent

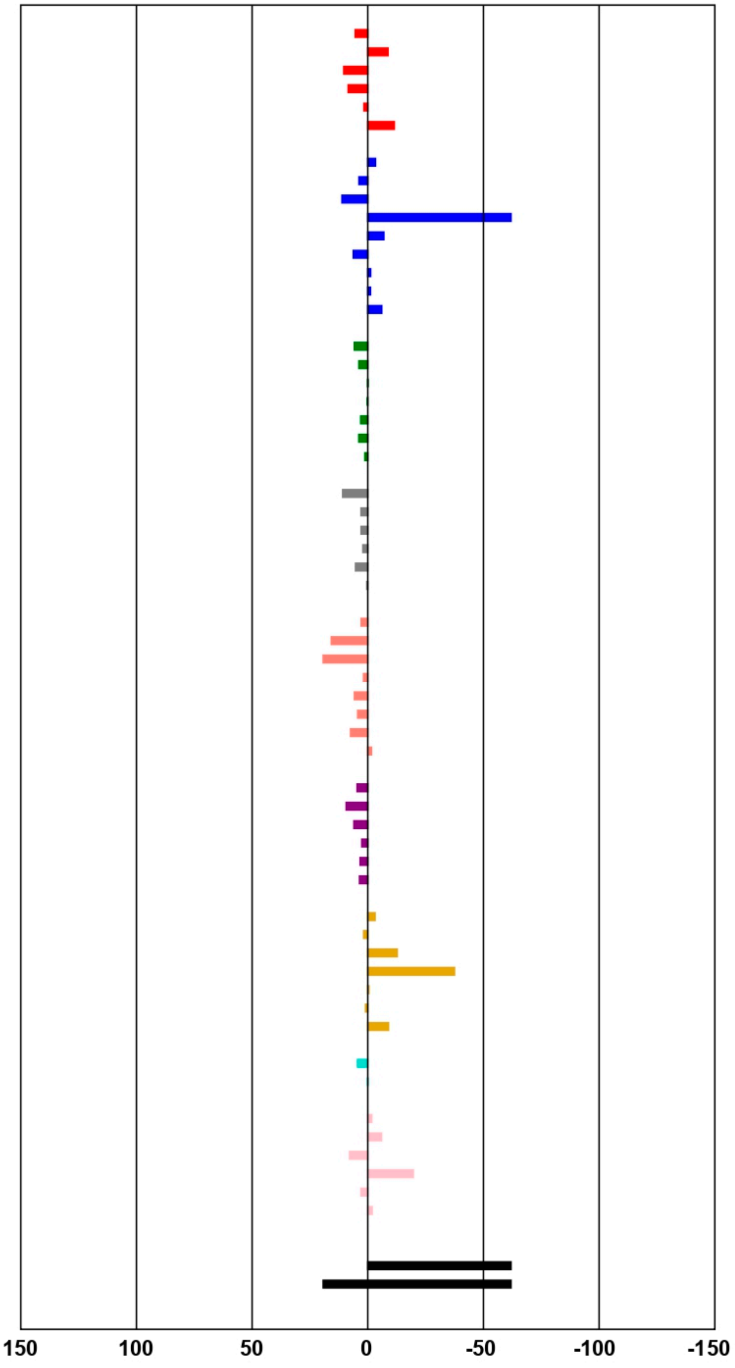

Figure S58. Mean graph of compound (**6i**) with colour codes for each cell line.

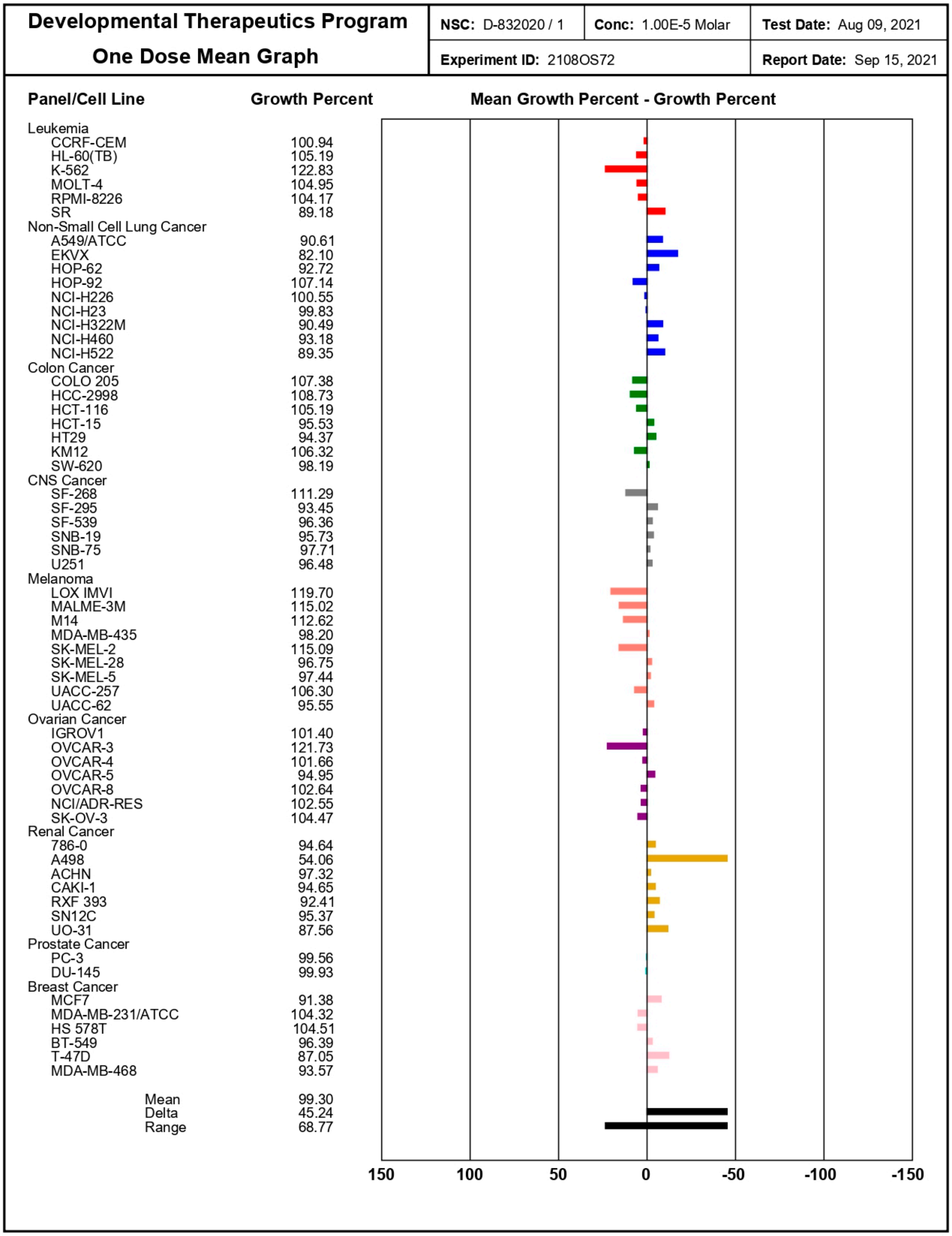

Figure S59. Mean graph of compound (**6j**) with colour codes for each cell line.

Developmental Therapeutics Program	NSC: D-831564 / 1	Conc: 1.00E-5 Molar	Test Date: Jul 12, 2021
One Dose Mean Graph	Experiment ID: 2107OS55		Report Date: Sep 05, 2021

Panel/Cell Line	Growth Percent
Leukemia	
CCRF-CEM	9.59
HL-60(TB)	80.67
K-562	63.82
MOLT-4	87.12
RPMI-8226	30.69
SR	74.44
Non-Small Cell Lung Cancer	
A549/ATCC	92.43
EKVX	95.35
HOP-62	100.96
NCI-H226	85.32
NCI-H23	86.23
NCI-H322M	94.20
NCI-H460	97.86
NCI-H522	87.86
Colon Cancer	
COLO 205	99.51
HCC-2998	97.07
HCT-116	86.68
HCT-15	63.34
HT29	84.40
KM12	88.86
SW-620	105.05
CNS Cancer	
SF-268	112.80
SF-295	92.93
SF-539	92.48
SNB-19	98.33
SNB-75	67.09
U251	90.78
Melanoma	
LOX IMVI	82.97
MALME-3M	105.36
M14	110.28
MDA-MB-435	96.14
SK-MEL-28	100.67
SK-MEL-5	78.55
UACC-257	112.25
UACC-62	87.74
Ovarian Cancer	
IGROV1	110.44
OVCAR-4	95.94
OVCAR-5	93.42
OVCAR-8	96.14
NCI/ADR-RES	87.62
SK-OV-3	102.33
Renal Cancer	
786-0	86.57
ACHN	90.75
CAKI-1	88.73
SN12C	90.51
TK-10	111.26
UO-31	85.50
Prostate Cancer	
PC-3	92.78
DU-145	97.50
Breast Cancer	
MCF7	91.79
MDA-MB-231/ATCC	82.36
HS 578T	91.89
BT-549	145.40
T-47D	91.00
MDA-MB-468	98.01
Mean	90.36
Delta	80.77
Range	135.81

Mean Growth Percent - Growth Percent

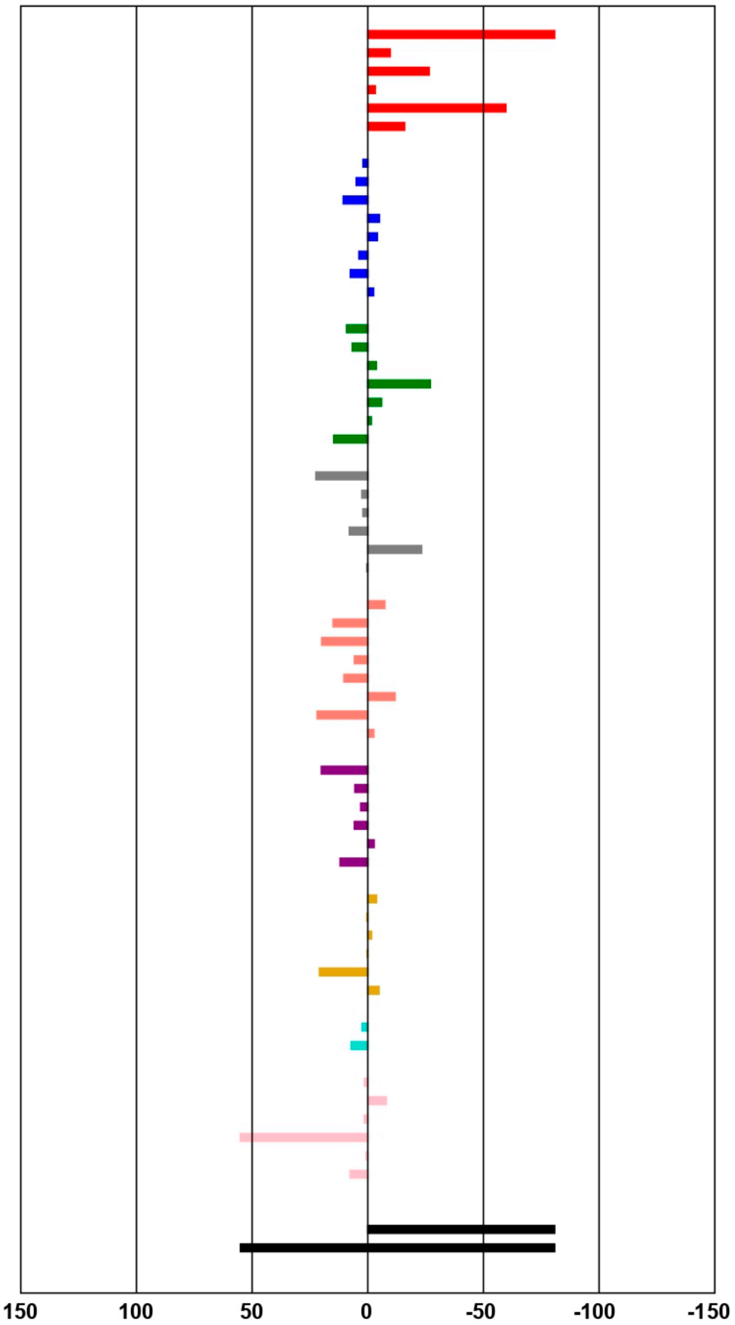

Figure S60. Mean graph of compound (**6k**) with colour codes for each cell line.

Developmental Therapeutics Program One Dose Mean Graph	NSC: D-831565 / 1	Conc: 1.00E-5 Molar	Test Date: Jul 12, 2021
	Experiment ID: 2107OS55		Report Date: Sep 05, 2021

Panel/Cell Line	Growth Percent
Leukemia	
CCRF-CEM	106.19
HL-60(TB)	104.20
K-562	102.19
MOLT-4	104.37
RPMI-8226	99.64
SR	95.28
Non-Small Cell Lung Cancer	
A549/ATCC	102.04
EKVX	108.33
HOP-62	102.90
HOP-92	109.63
NCI-H226	92.29
NCI-H23	95.08
NCI-H322M	98.23
NCI-H460	104.70
NCI-H522	93.04
Colon Cancer	
COLO 205	102.05
HCC-2998	102.74
HCT-116	92.94
HCT-15	81.64
HT29	88.46
KM12	98.90
SW-620	102.37
CNS Cancer	
SF-268	117.13
SF-295	98.95
SF-539	102.50
SNB-19	110.77
SNB-75	93.64
U251	105.59
Melanoma	
LOX IMVI	92.22
MALME-3M	109.74
M14	111.28
MDA-MB-435	98.04
SK-MEL-28	104.93
SK-MEL-5	87.20
UACC-257	107.73
UACC-62	94.24
Ovarian Cancer	
IGROV1	108.96
OVCAR-4	106.19
OVCAR-5	103.97
OVCAR-8	101.14
NCI/ADR-RES	97.95
SK-OV-3	102.82
Renal Cancer	
786-0	106.83
ACHN	105.59
CAKI-1	96.73
RXF 393	98.56
SN12C	96.34
TK-10	119.14
UO-31	93.25
Prostate Cancer	
PC-3	112.49
DU-145	99.58
Breast Cancer	
MCF7	95.81
MDA-MB-231/ATCC	96.44
HS 578T	99.39
BT-549	121.29
T-47D	95.67
MDA-MB-468	100.86
Mean	101.41
Delta	19.77
Range	39.65

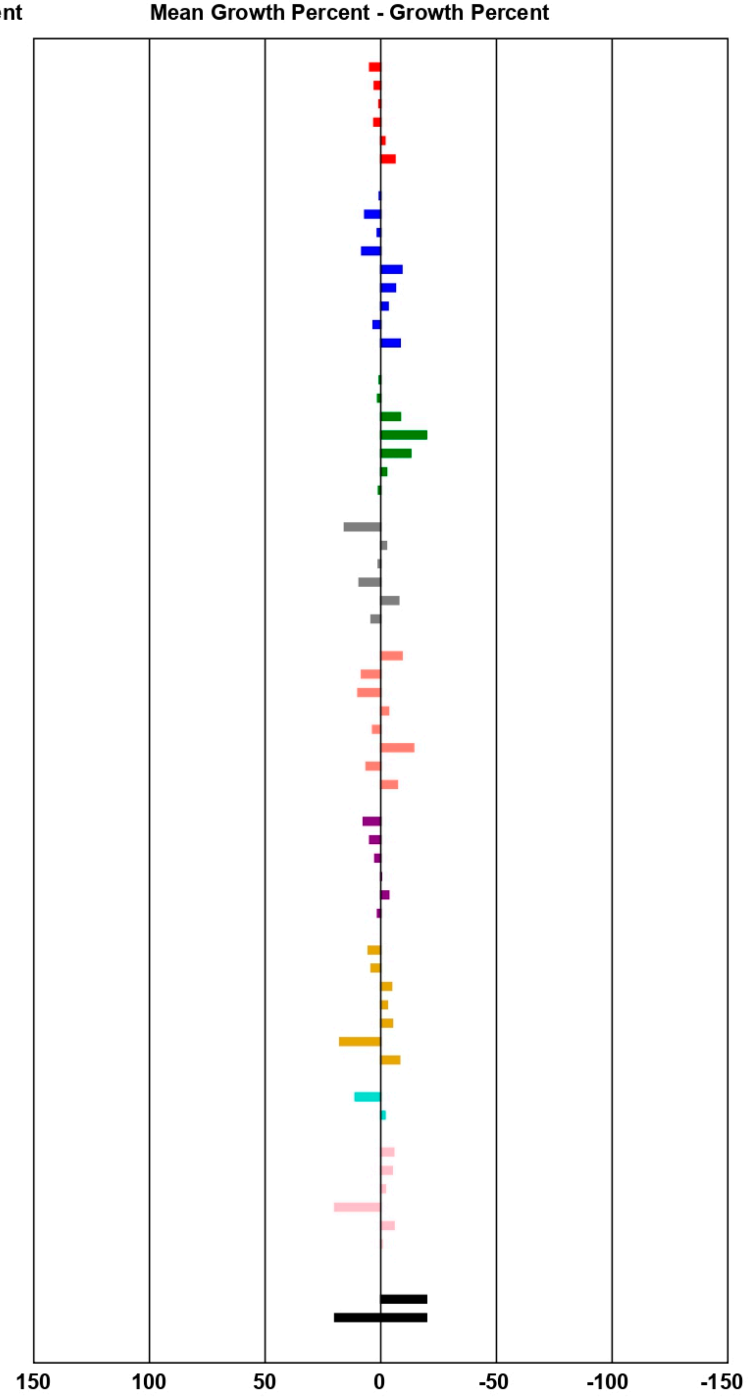

Figure S61. Mean graph of compound (**6l**) with colour codes for each cell line.

Developmental Therapeutics Program | **NSC:** D-831566 / 1 | **Conc:** 1.00E-5 Molar | **Test Date:** Jul 12, 2021

One Dose Mean Graph | **Experiment ID:** 2107OS55 | **Report Date:** Sep 05, 2021

Panel/Cell Line	Growth Percent
**Leukemia**	
CCRF-CEM	74.07
HL-60(TB)	77.34
K-562	45.34
MOLT-4	82.52
RPMI-8226	75.13
SR	76.72
**Non-Small Cell Lung Cancer**	
A549/ATCC	88.67
EKVX	99.55
HOP-62	95.26
NCI-H226	93.54
NCI-H23	86.03
NCI-H322M	87.07
NCI-H460	93.45
NCI-H522	88.09
**Colon Cancer**	
COLO 205	99.93
HCC-2998	95.72
HCT-116	71.74
HCT-15	48.60
HT29	74.88
KM12	73.95
SW-620	95.14
**CNS Cancer**	
SF-268	100.80
SF-295	89.48
SF-539	83.78
SNB-19	108.60
SNB-75	82.75
U251	89.20
**Melanoma**	
LOX IMVI	80.31
MALME-3M	87.61
M14	95.97
MDA-MB-435	90.35
SK-MEL-28	89.93
SK-MEL-5	71.26
UACC-257	110.14
UACC-62	95.09
**Ovarian Cancer**	
IGROV1	100.80
OVCAR-4	98.67
OVCAR-5	90.92
OVCAR-8	94.33
NCI/ADR-RES	88.12
SK-OV-3	100.69
**Renal Cancer**	
786-0	64.51
ACHN	83.79
CAKI-1	77.99
RXF 393	-2.68
SN12C	93.55
TK-10	100.16
UO-31	85.74
**Prostate Cancer**	
PC-3	99.19
DU-145	90.80
**Breast Cancer**	
MCF7	89.92
MDA-MB-231/ATCC	79.38
HS 578T	79.73
BT-549	126.77
T-47D	75.09
MDA-MB-468	91.06
Mean	85.83
Delta	88.51
Range	129.45

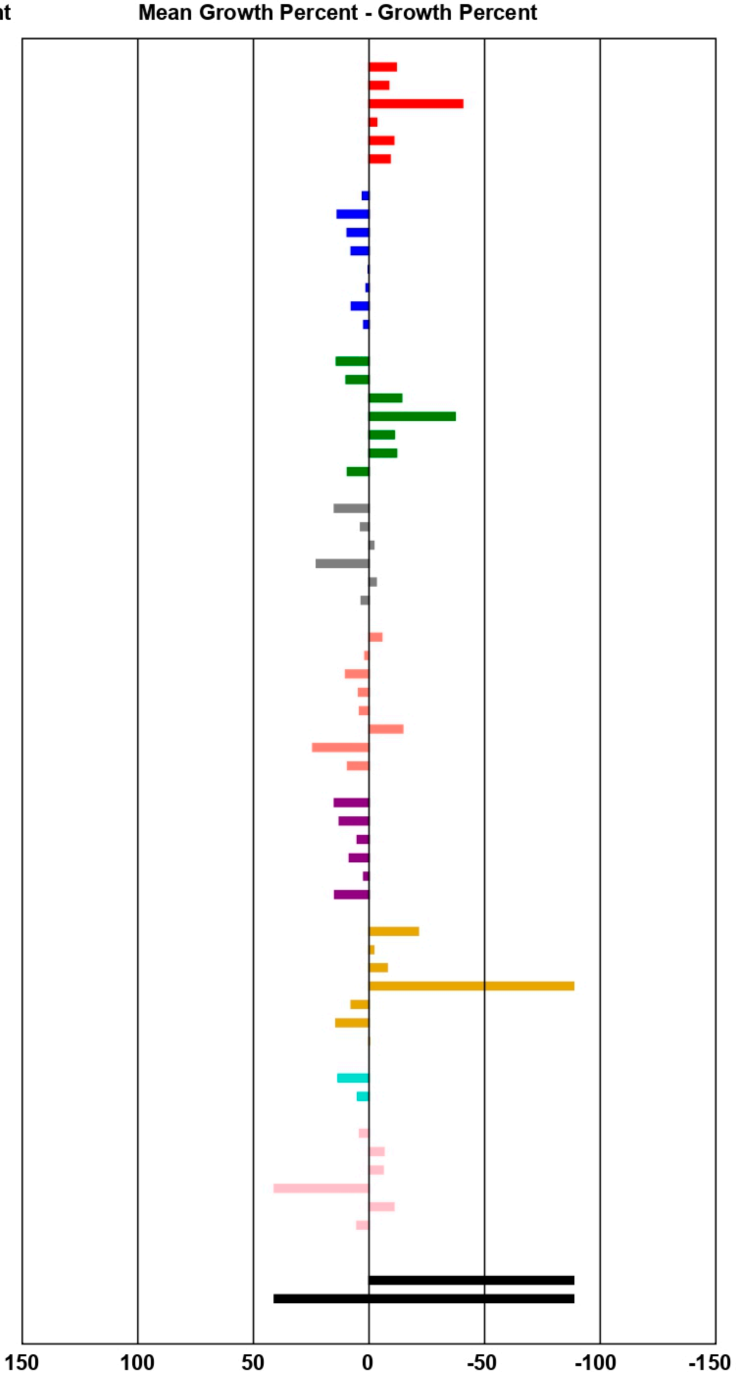

Figure S62. Mean graph of compound (**6m**) with colour codes for each cell line.

Developmental Therapeutics Program

One Dose Mean Graph

**NSC:** D-831567 / 1	**Conc:** 1.00E-5 Molar	**Test Date:** Jul 12, 2021	
**Experiment ID:** 2107OS55		**Report Date:** Sep 05, 2021	

Panel/Cell Line	Growth Percent
**Leukemia**	
CCRF-CEM	30.92
HL-60(TB)	43.31
K-562	19.85
MOLT-4	36.49
RPMI-8226	30.37
SR	23.63
**Non-Small Cell Lung Cancer**	
A549/ATCC	52.56
EKVX	77.13
HOP-62	72.66
HOP-92	5.27
NCI-H226	54.91
NCI-H23	70.77
NCI-H322M	92.55
NCI-H460	47.89
NCI-H522	69.31
**Colon Cancer**	
COLO 205	55.73
HCC-2998	52.23
HCT-116	42.71
HCT-15	18.39
HT29	62.27
KM12	44.86
SW-620	40.92
**CNS Cancer**	
SF-268	68.12
SF-295	49.00
SF-539	60.23
SNB-19	80.07
SNB-75	68.69
U251	60.61
**Melanoma**	
LOX IMVI	37.64
MALME-3M	76.21
M14	53.55
MDA-MB-435	69.63
SK-MEL-28	65.77
SK-MEL-5	32.38
UACC-257	95.23
UACC-62	77.07
**Ovarian Cancer**	
IGROV1	102.18
OVCAR-4	72.39
OVCAR-5	84.66
OVCAR-8	50.53
NCI/ADR-RES	48.89
SK-OV-3	72.02
**Renal Cancer**	
786-0	37.65
ACHN	54.77
CAKI-1	44.37
SN12C	67.93
TK-10	80.63
UO-31	42.92
**Prostate Cancer**	
PC-3	39.10
DU-145	74.18
**Breast Cancer**	
MCF7	54.97
MDA-MB-231/ATCC	34.92
HS 578T	49.40
BT-549	112.06
T-47D	41.34
MDA-MB-468	37.70
Mean	56.10
Delta	50.83
Range	106.79

Mean Growth Percent - Growth Percent

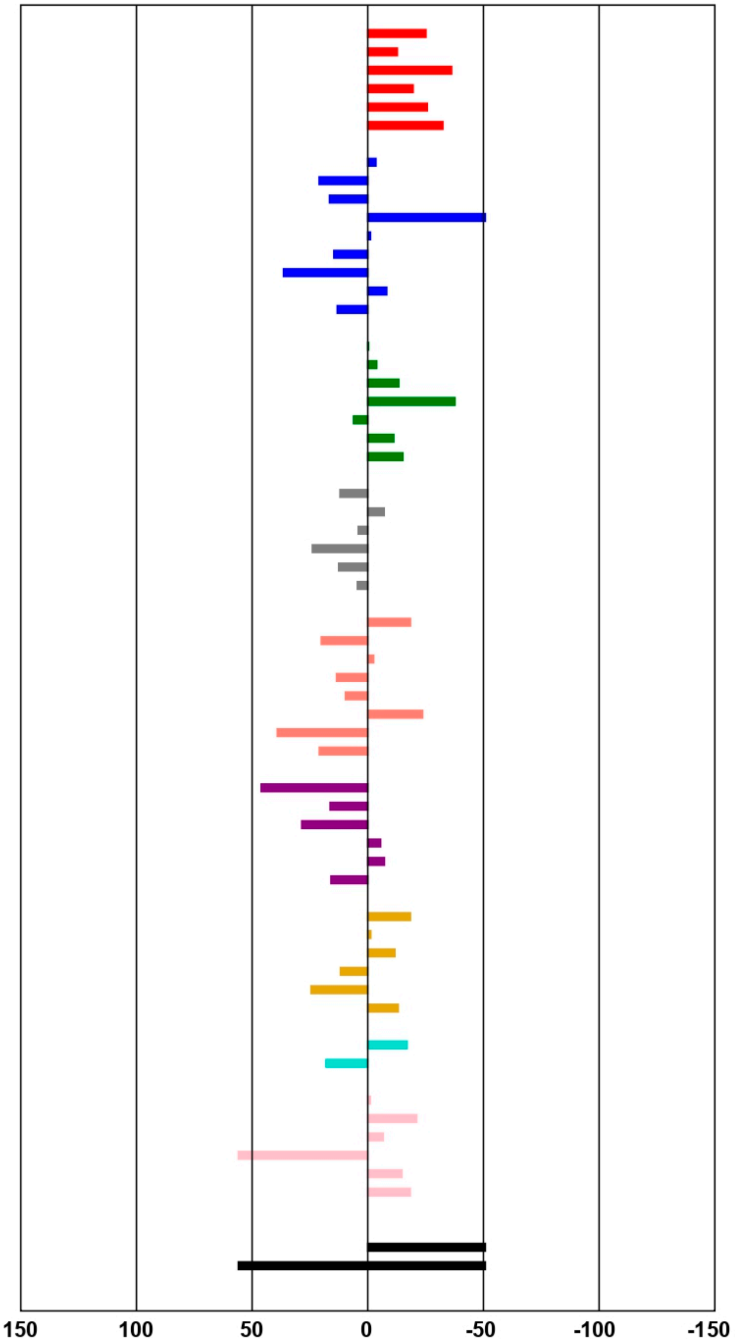

Figure S63. Mean graph of compound (**6n**) with colour codes for each cell line.

Developmental Therapeutics Program One Dose Mean Graph	NSC: D-831568 / 1	Conc: 1.00E-5 Molar	Test Date: Jul 12, 2021
	Experiment ID: 2107OS55		Report Date: Sep 05, 2021

Panel/Cell Line	Growth Percent
**Leukemia**	
CCRF-CEM	16.15
HL-60(TB)	86.73
K-562	81.10
MOLT-4	85.96
RPMI-8226	91.69
SR	72.68
**Non-Small Cell Lung Cancer**	
A549/ATCC	70.40
EKVX	88.95
HOP-62	56.70
NCI-H226	58.60
NCI-H23	73.45
NCI-H322M	86.55
NCI-H460	44.11
NCI-H522	79.74
**Colon Cancer**	
COLO 205	82.06
HCC-2998	91.05
HCT-116	61.12
HCT-15	77.80
HT29	83.69
KM12	89.14
SW-620	84.19
**CNS Cancer**	
SF-268	76.97
SF-295	77.54
SF-539	71.74
SNB-19	72.09
SNB-75	87.95
U251	81.45
**Melanoma**	
LOX IMVI	64.50
MALME-3M	83.99
M14	96.53
MDA-MB-435	84.21
SK-MEL-28	87.18
SK-MEL-5	80.18
UACC-257	104.26
UACC-62	82.81
**Ovarian Cancer**	
IGROV1	80.48
OVCAR-4	66.47
OVCAR-5	87.20
OVCAR-8	80.59
NCI/ADR-RES	63.60
SK-OV-3	75.41
**Renal Cancer**	
786-0	64.69
ACHN	47.23
CAKI-1	56.13
RXF 393	-10.55
SN12C	83.90
TK-10	98.11
UO-31	64.78
**Prostate Cancer**	
PC-3	80.51
DU-145	79.49
**Breast Cancer**	
MCF7	81.29
MDA-MB-231/ATCC	75.44
HS 578T	72.58
BT-549	103.13
T-47D	64.18
MDA-MB-468	65.12
Mean	74.87
Delta	85.42
Range	114.81

Mean Growth Percent - Growth Percent

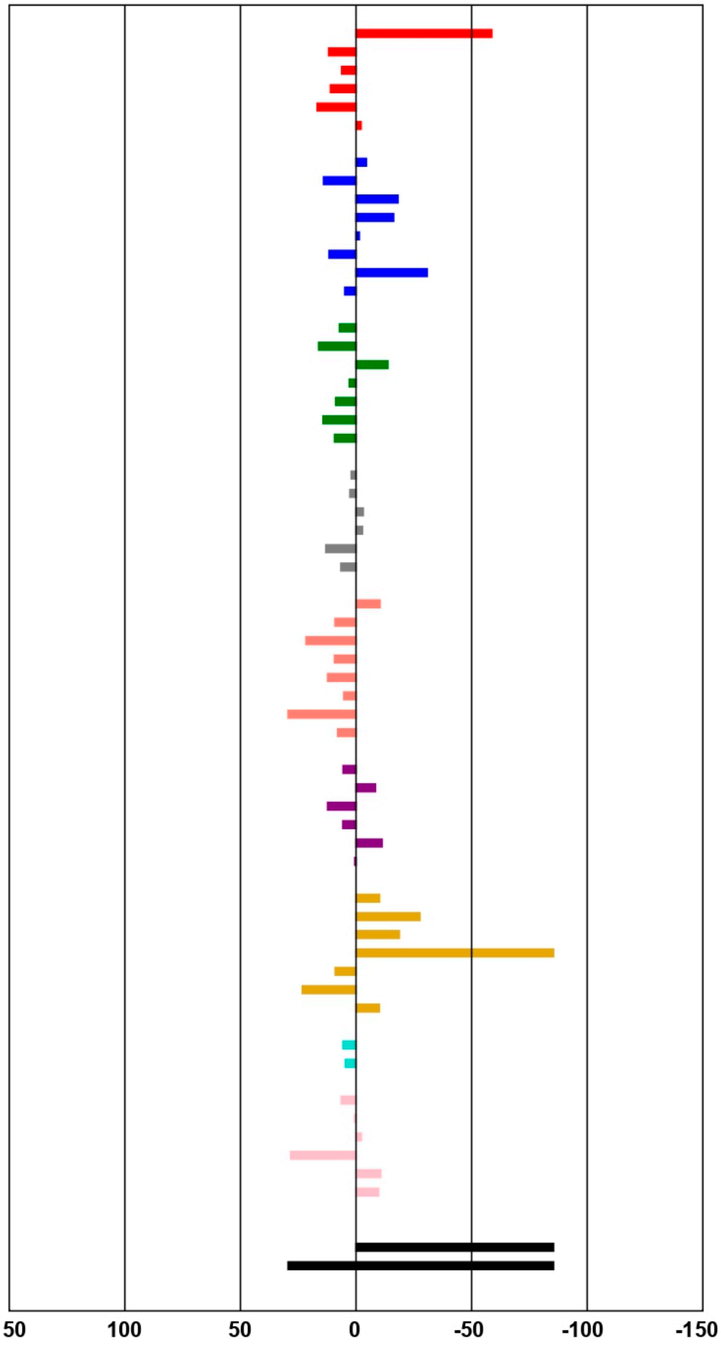

Figure S64. Mean graph of compound (**6o**) with colour codes for each cell line.

Developmental Therapeutics Program<br>One Dose Mean Graph	NSC: D-831569 / 1	Conc: 1.00E-5 Molar	Test Date: Jul 12, 2021
	Experiment ID: 2107OS55		Report Date: Sep 05, 2021

Panel/Cell Line	Growth Percent
Leukemia	
CCRF-CEM	85.82
HL-60(TB)	90.61
K-562	78.88
MOLT-4	87.63
RPMI-8226	95.83
SR	70.70
Non-Small Cell Lung Cancer	
A549/ATCC	56.39
EKVX	81.53
HOP-62	47.41
HOP-92	28.20
NCI-H226	63.33
NCI-H23	64.95
NCI-H322M	79.07
NCI-H460	33.88
NCI-H522	80.86
Colon Cancer	
COLO 205	80.05
HCC-2998	87.14
HCT-116	42.53
HCT-15	66.40
HT29	76.70
KM12	78.33
SW-620	78.45
CNS Cancer	
SF-268	68.67
SF-295	77.21
SF-539	63.81
SNB-19	56.77
SNB-75	65.20
U251	66.32
Melanoma	
LOX IMVI	56.43
MALME-3M	74.98
M14	88.58
MDA-MB-435	80.27
SK-MEL-28	78.34
SK-MEL-5	81.24
UACC-257	103.44
UACC-62	79.32
Ovarian Cancer	
IGROV1	70.96
OVCAR-4	53.49
OVCAR-5	80.39
OVCAR-8	69.16
NCI/ADR-RES	59.80
SK-OV-3	59.21
Renal Cancer	
786-0	54.33
ACHN	33.98
CAKI-1	57.58
RXF 393	-12.34
SN12C	68.31
TK-10	90.62
UO-31	65.09
Prostate Cancer	
PC-3	80.90
DU-145	77.75
Breast Cancer	
MCF7	80.87
MDA-MB-231/ATCC	70.41
HS 578T	62.83
BT-549	103.75
T-47D	41.28
MDA-MB-468	67.53
Mean	68.97
Delta	81.31
Range	116.09

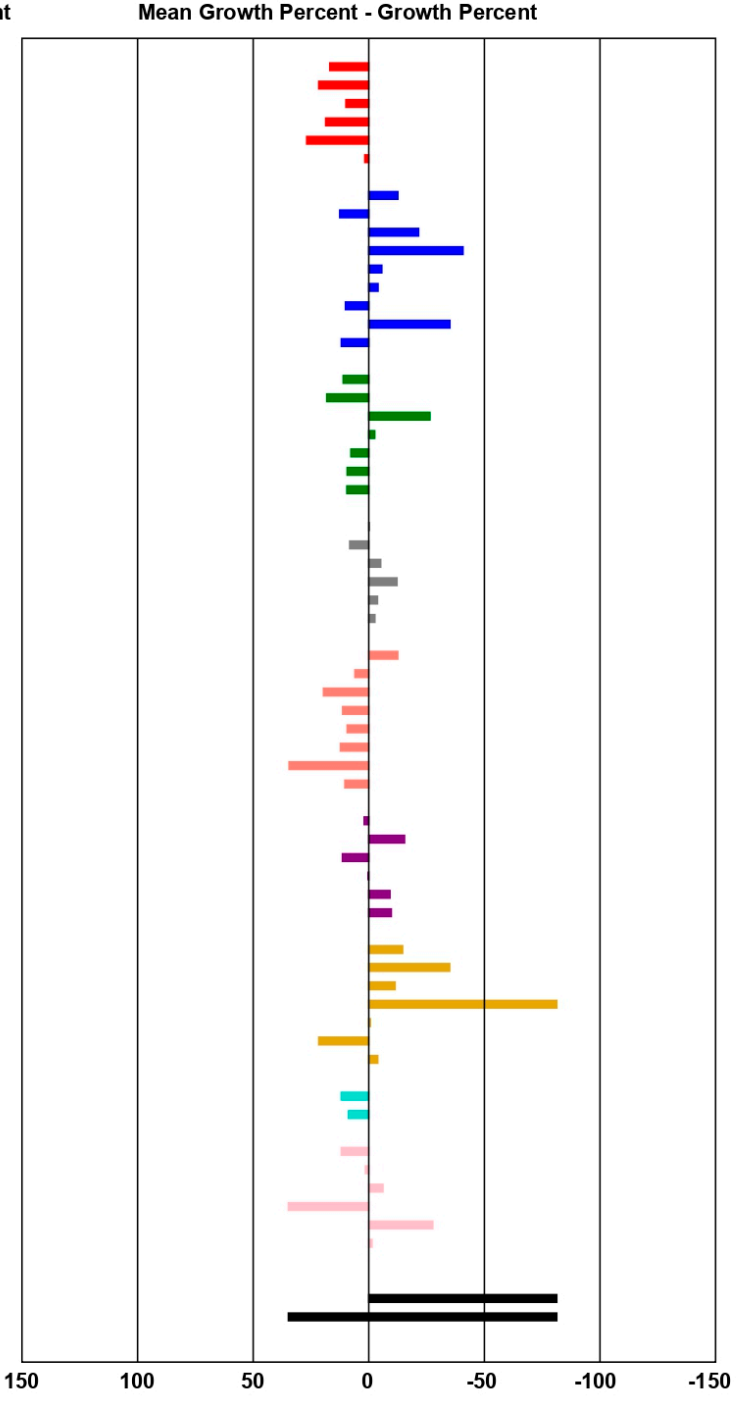

Figure S65. Mean graph of compound (**6p**) with colour codes for each cell line.

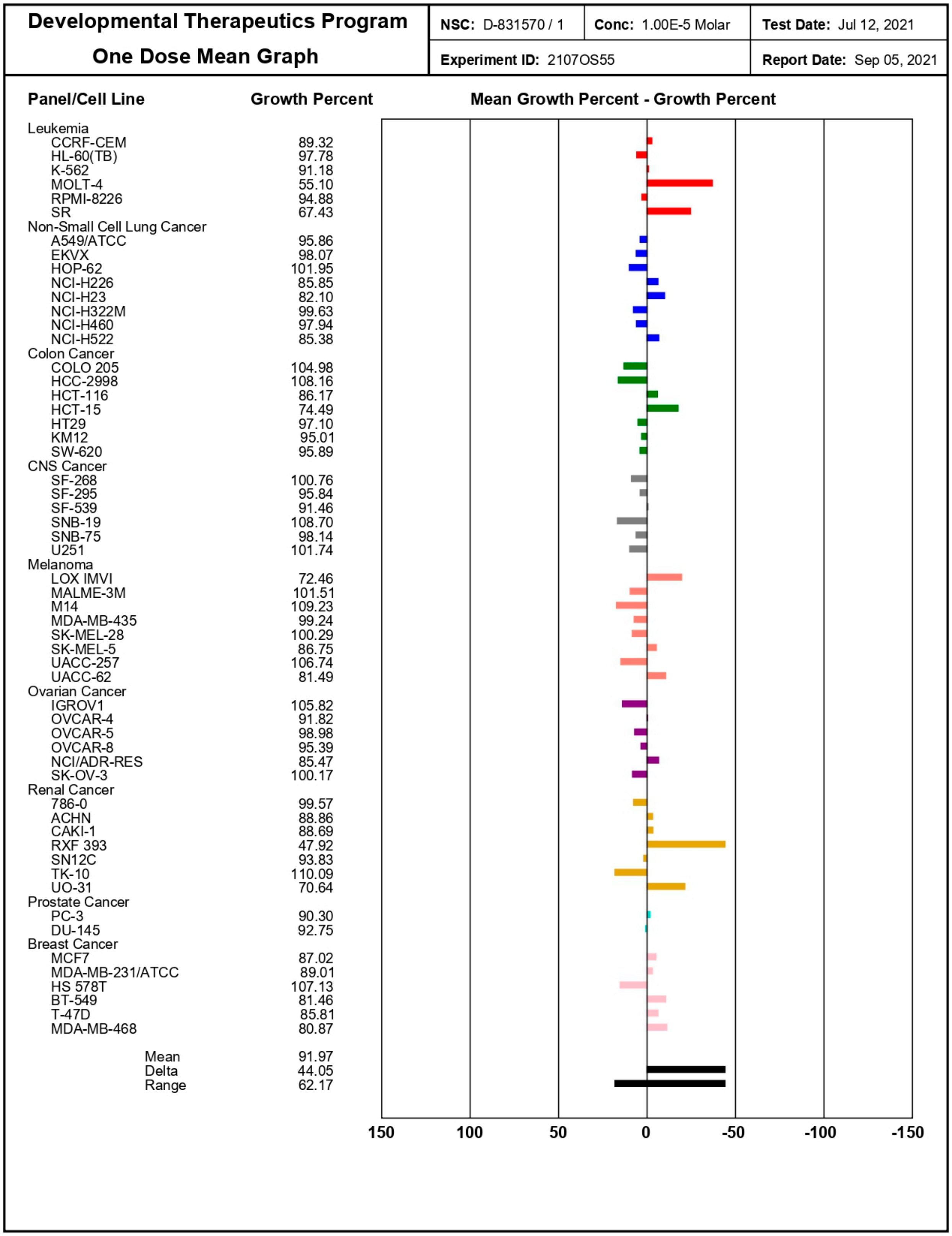

Developmental Therapeutics Program
One Dose Mean Graph

NSC: D-831570 / 1	Conc: 1.00E-5 Molar	Test Date: Jul 12, 2021
Experiment ID: 2107OS55		Report Date: Sep 05, 2021

Panel/Cell Line	Growth Percent
Leukemia	
CCRF-CEM	89.32
HL-60(TB)	97.78
K-562	91.18
MOLT-4	55.10
RPMI-8226	94.88
SR	67.43
Non-Small Cell Lung Cancer	
A549/ATCC	95.86
EKVX	98.07
HOP-62	101.95
NCI-H226	85.85
NCI-H23	82.10
NCI-H322M	99.63
NCI-H460	97.94
NCI-H522	85.38
Colon Cancer	
COLO 205	104.98
HCC-2998	108.16
HCT-116	86.17
HCT-15	74.49
HT29	97.10
KM12	95.01
SW-620	95.89
CNS Cancer	
SF-268	100.76
SF-295	95.84
SF-539	91.46
SNB-19	108.70
SNB-75	98.14
U251	101.74
Melanoma	
LOX IMVI	72.46
MALME-3M	101.51
M14	109.23
MDA-MB-435	99.24
SK-MEL-28	100.29
SK-MEL-5	86.75
UACC-257	106.74
UACC-62	81.49
Ovarian Cancer	
IGROV1	105.82
OVCAR-4	91.82
OVCAR-5	98.98
OVCAR-8	95.39
NCI/ADR-RES	85.47
SK-OV-3	100.17
Renal Cancer	
786-0	99.57
ACHN	88.86
CAKI-1	88.69
RXF 393	47.92
SN12C	93.83
TK-10	110.09
UO-31	70.64
Prostate Cancer	
PC-3	90.30
DU-145	92.75
Breast Cancer	
MCF7	87.02
MDA-MB-231/ATCC	89.01
HS 578T	107.13
BT-549	81.46
T-47D	85.81
MDA-MB-468	80.87
Mean	91.97
Delta	44.05
Range	62.17

Figure S66. Mean graph of compound (**6q**) with colour codes for each cell line.

Developmental Therapeutics Program One Dose Mean Graph	NSC: D-831571 / 1	Conc: 1.00E-5 Molar	Test Date: Jul 12, 2021
	Experiment ID: 2107OS55		Report Date: Sep 05, 2021

Panel/Cell Line	Growth Percent
**Leukemia**	
CCRF-CEM	88.64
HL-60(TB)	89.11
K-562	69.12
MOLT-4	91.47
RPMI-8226	92.32
SR	77.62
**Non-Small Cell Lung Cancer**	
A549/ATCC	99.54
EKVX	102.13
HOP-62	101.66
HOP-92	72.50
NCI-H226	103.08
NCI-H23	99.31
NCI-H322M	97.85
NCI-H460	101.45
NCI-H522	94.02
**Colon Cancer**	
COLO 205	106.54
HCC-2998	110.29
HCT-116	93.07
HCT-15	71.33
HT29	92.68
KM12	86.75
SW-620	96.95
**CNS Cancer**	
SF-268	104.96
SF-295	101.47
SF-539	95.82
SNB-19	107.61
SNB-75	91.55
U251	97.82
**Melanoma**	
LOX IMVI	89.85
MALME-3M	95.07
M14	95.90
MDA-MB-435	93.83
SK-MEL-28	96.90
SK-MEL-5	95.93
UACC-257	109.21
UACC-62	89.38
**Ovarian Cancer**	
IGROV1	113.10
OVCAR-4	99.47
OVCAR-5	93.71
OVCAR-8	98.86
NCI/ADR-RES	97.12
SK-OV-3	96.81
**Renal Cancer**	
786-0	97.05
ACHN	92.95
CAKI-1	77.92
RXF 393	18.29
SN12C	99.71
TK-10	116.28
UO-31	83.22
**Prostate Cancer**	
PC-3	96.22
DU-145	97.09
**Breast Cancer**	
MCF7	94.52
MDA-MB-231/ATCC	90.09
HS 578T	100.78
BT-549	110.58
T-47D	100.47
MDA-MB-468	93.03
Mean	94.21
Delta	75.92
Range	97.99

Mean Growth Percent - Growth Percent

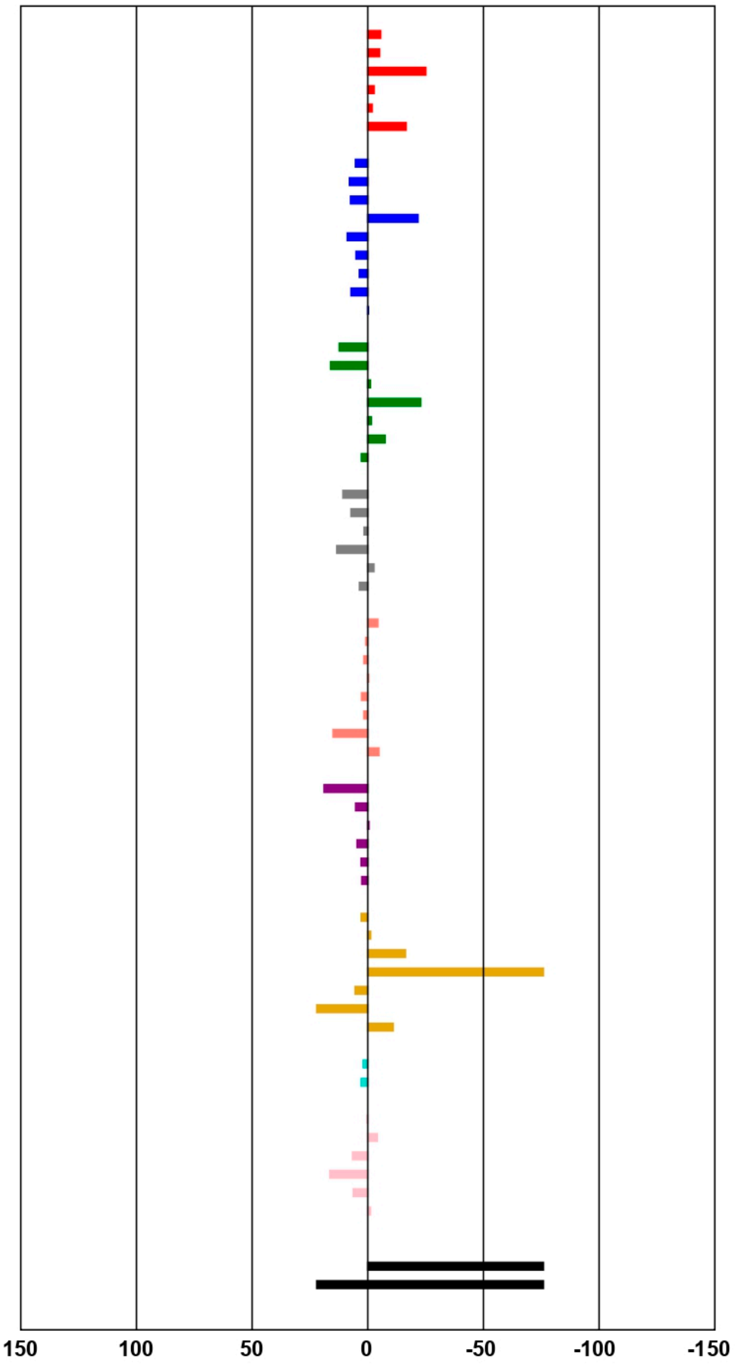

Figure S67. Mean graph of compound (**6r**) with colour codes for each cell line.

Developmental Therapeutics Program	NSC: D-831572 / 1	Conc: 1.00E-5 Molar	Test Date: Jul 12, 2021
One Dose Mean Graph	Experiment ID: 2107OS55		Report Date: Sep 05, 2021

Panel/Cell Line	Growth Percent
Leukemia	
CCRF-CEM	95.67
HL-60(TB)	85.65
K-562	97.22
MOLT-4	104.52
RPMI-8226	89.39
SR	78.51
Non-Small Cell Lung Cancer	
A549/ATCC	99.14
EKVX	97.09
HOP-62	101.85
HOP-92	60.47
NCI-H226	97.50
NCI-H23	96.96
NCI-H322M	103.65
NCI-H460	100.34
NCI-H522	85.60
Colon Cancer	
COLO 205	99.51
HCC-2998	107.99
HCT-116	84.93
HCT-15	90.09
HT29	90.58
KM12	101.26
SW-620	94.83
CNS Cancer	
SF-268	100.86
SF-295	99.55
SF-539	93.86
SNB-19	101.35
SNB-75	99.44
U251	97.82
Melanoma	
LOX IMVI	94.92
MALME-3M	119.87
M14	114.75
MDA-MB-435	95.32
SK-MEL-28	99.33
SK-MEL-5	86.67
UACC-257	107.06
UACC-62	85.64
Ovarian Cancer	
IGROV1	102.50
OVCAR-4	95.65
OVCAR-5	95.58
OVCAR-8	96.15
NCI/ADR-RES	99.76
SK-OV-3	91.23
Renal Cancer	
786-0	91.80
ACHN	102.77
CAKI-1	87.75
RXF 393	66.52
SN12C	97.28
TK-10	103.82
UO-31	92.71
Prostate Cancer	
PC-3	99.50
DU-145	92.87
Breast Cancer	
MCF7	87.36
MDA-MB-231/ATCC	88.78
HS 578T	109.36
BT-549	106.30
T-47D	93.45
MDA-MB-468	88.46
Mean	95.59
Delta	35.12
Range	59.40

Mean Growth Percent - Growth Percent

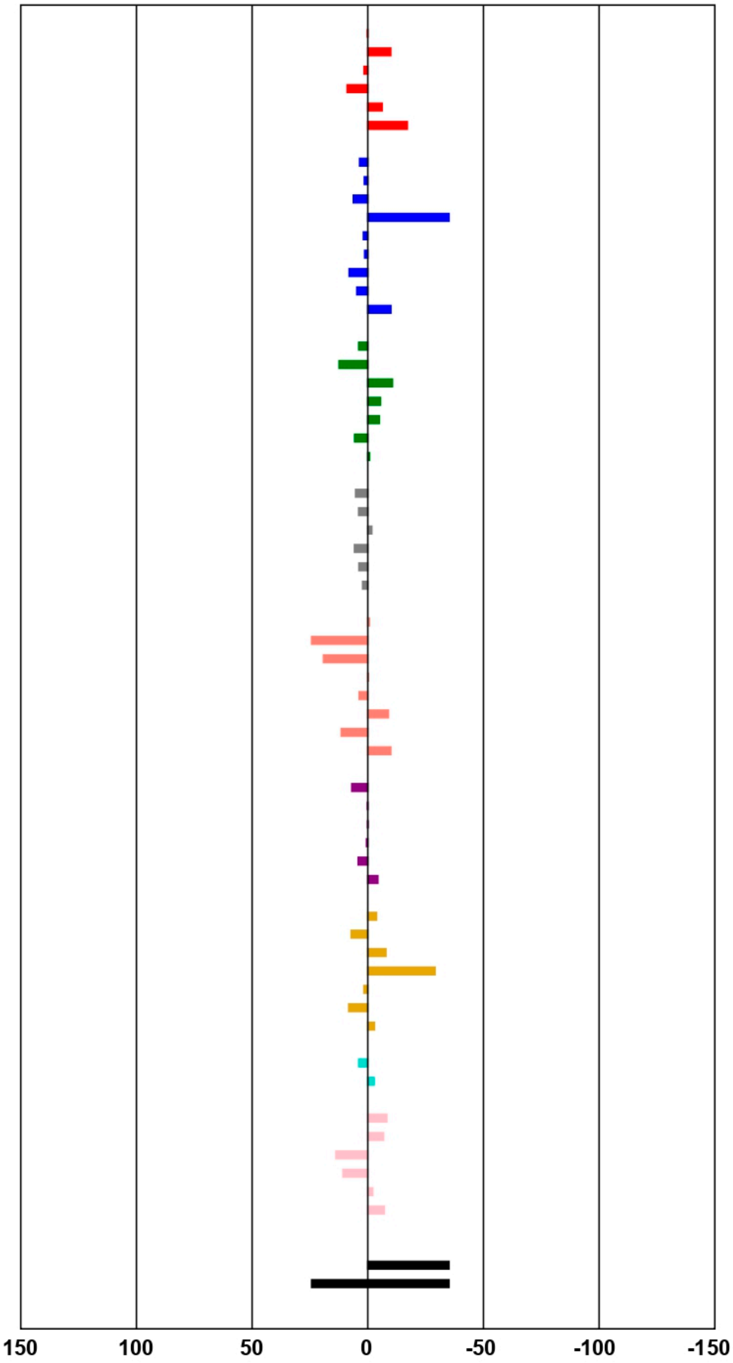

Figure S68. Mean graph of compound (**6s**) with colour codes for each cell line.

Developmental Therapeutics Program<br>One Dose Mean Graph	NSC: D-831573 / 1	Conc: 1.00E-5 Molar	Test Date: Jul 12, 2021
	Experiment ID: 2107OS55		Report Date: Sep 05, 2021

Panel/Cell Line	Growth Percent
**Leukemia**	
CCRF-CEM	58.81
HL-60(TB)	74.88
K-562	59.02
MOLT-4	74.54
RPMI-8226	66.24
SR	63.53
**Non-Small Cell Lung Cancer**	
A549/ATCC	95.24
EKVX	88.83
HOP-62	93.78
NCI-H226	88.98
NCI-H23	92.68
NCI-H322M	94.36
NCI-H460	92.30
NCI-H522	82.06
**Colon Cancer**	
COLO 205	92.69
HCC-2998	100.12
HCT-116	77.96
HCT-15	53.76
HT29	80.33
KM12	82.14
SW-620	88.70
**CNS Cancer**	
SF-268	91.40
SF-295	84.07
SF-539	84.12
SNB-19	98.18
SNB-75	88.79
U251	88.60
**Melanoma**	
LOX IMVI	73.66
MALME-3M	88.73
M14	91.65
MDA-MB-435	87.52
SK-MEL-28	90.66
SK-MEL-5	70.89
UACC-257	107.92
UACC-62	84.57
**Ovarian Cancer**	
IGROV1	91.27
OVCAR-4	91.67
OVCAR-5	90.30
OVCAR-8	92.22
NCI/ADR-RES	87.38
SK-OV-3	83.21
**Renal Cancer**	
786-0	71.04
ACHN	88.34
CAKI-1	78.83
RXF 393	-6.08
SN12C	91.03
TK-10	107.32
UO-31	82.58
**Prostate Cancer**	
PC-3	81.02
DU-145	84.72
**Breast Cancer**	
MCF7	90.47
MDA-MB-231/ATCC	81.32
HS 578T	90.23
BT-549	109.07
T-47D	79.20
MDA-MB-468	73.32
Mean	83.40
Delta	89.48
Range	115.15

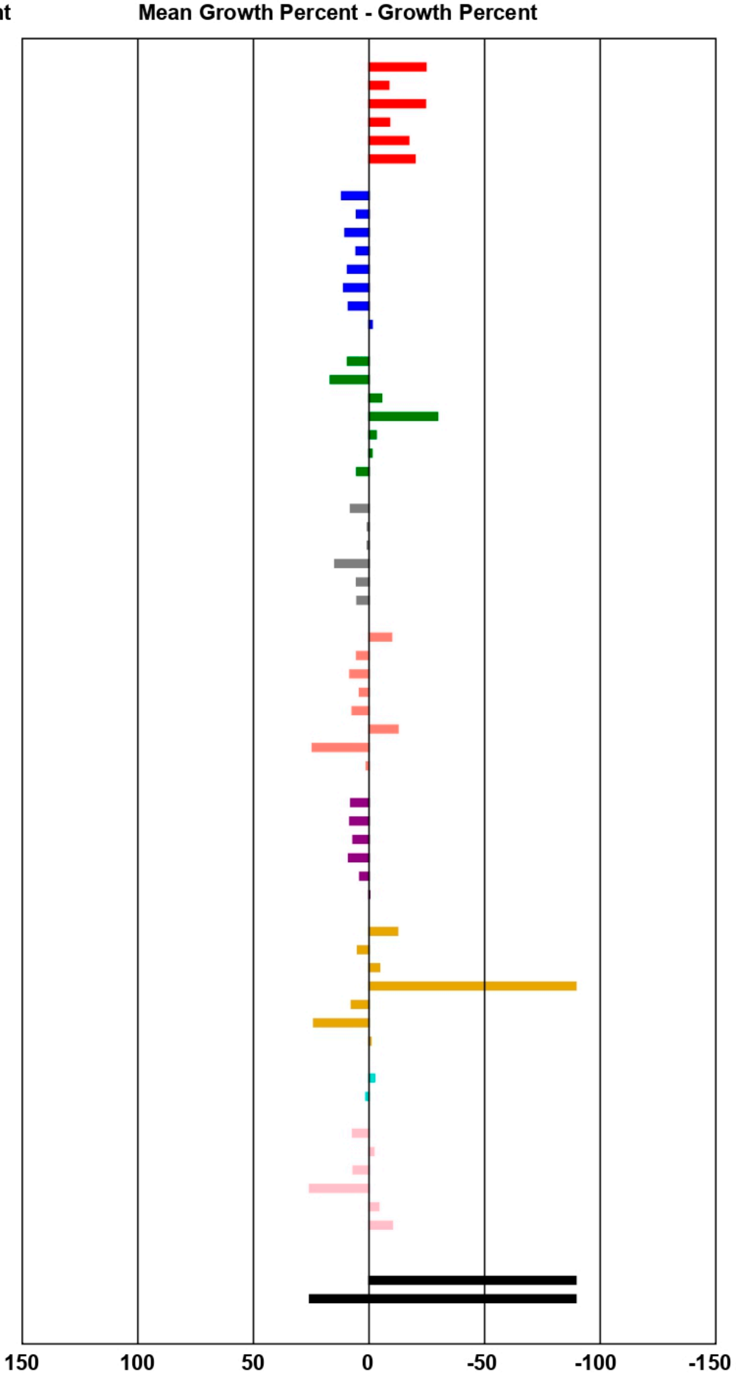

Figure S69. Mean graph of compound (**6t**) with colour codes for each cell line.

Developmental Therapeutics Program<br>One Dose Mean Graph	NSC: D-832021 / 1	Conc: 1.00E-5 Molar	Test Date: Aug 09, 2021
	Experiment ID: 2108OS72		Report Date: Sep 15, 2021

Panel/Cell Line	Growth Percent
**Leukemia**	
HL-60(TB)	119.72
K-562	114.75
MOLT-4	113.53
RPMI-8226	115.73
SR	107.43
**Non-Small Cell Lung Cancer**	
A549/ATCC	94.34
EKVX	92.64
HOP-62	92.33
HOP-92	92.44
NCI-H226	102.89
NCI-H23	104.64
NCI-H322M	96.81
NCI-H460	100.58
NCI-H522	85.97
**Colon Cancer**	
COLO 205	104.47
HCC-2998	105.97
HCT-116	99.40
HCT-15	94.19
HT29	96.61
KM12	100.46
SW-620	99.18
**CNS Cancer**	
SF-268	103.93
SF-295	95.02
SF-539	96.89
SNB-19	99.42
SNB-75	87.33
U251	93.05
**Melanoma**	
LOX IMVI	111.06
MALME-3M	108.48
M14	112.48
MDA-MB-435	91.41
SK-MEL-2	107.98
SK-MEL-28	102.71
SK-MEL-5	98.72
UACC-257	104.39
UACC-62	93.43
**Ovarian Cancer**	
IGROV1	102.13
OVCAR-3	109.90
OVCAR-4	95.48
OVCAR-5	94.32
OVCAR-8	101.76
NCI/ADR-RES	106.29
SK-OV-3	102.04
**Renal Cancer**	
786-0	95.49
A498	73.18
ACHN	97.82
CAKI-1	93.50
RXF 393	84.16
SN12C	99.38
UO-31	81.54
**Prostate Cancer**	
PC-3	95.79
DU-145	103.49
**Breast Cancer**	
MCF7	90.35
MDA-MB-231/ATCC	96.14
HS 578T	95.61
BT-549	109.65
T-47D	85.73
MDA-MB-468	90.61
Mean	99.05
Delta	25.87
Range	46.54

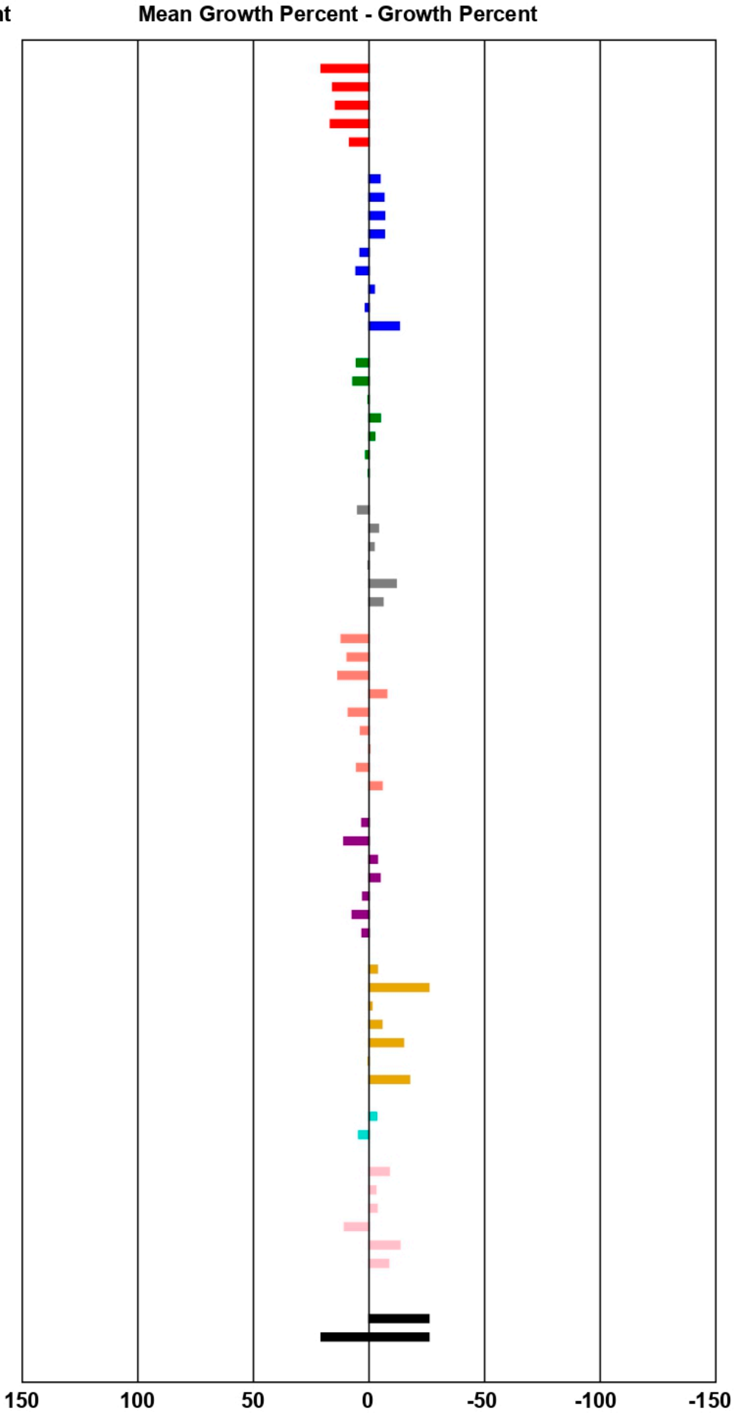

Figure S70. Mean graph of compound (**11a**) with colour codes for each cell line.

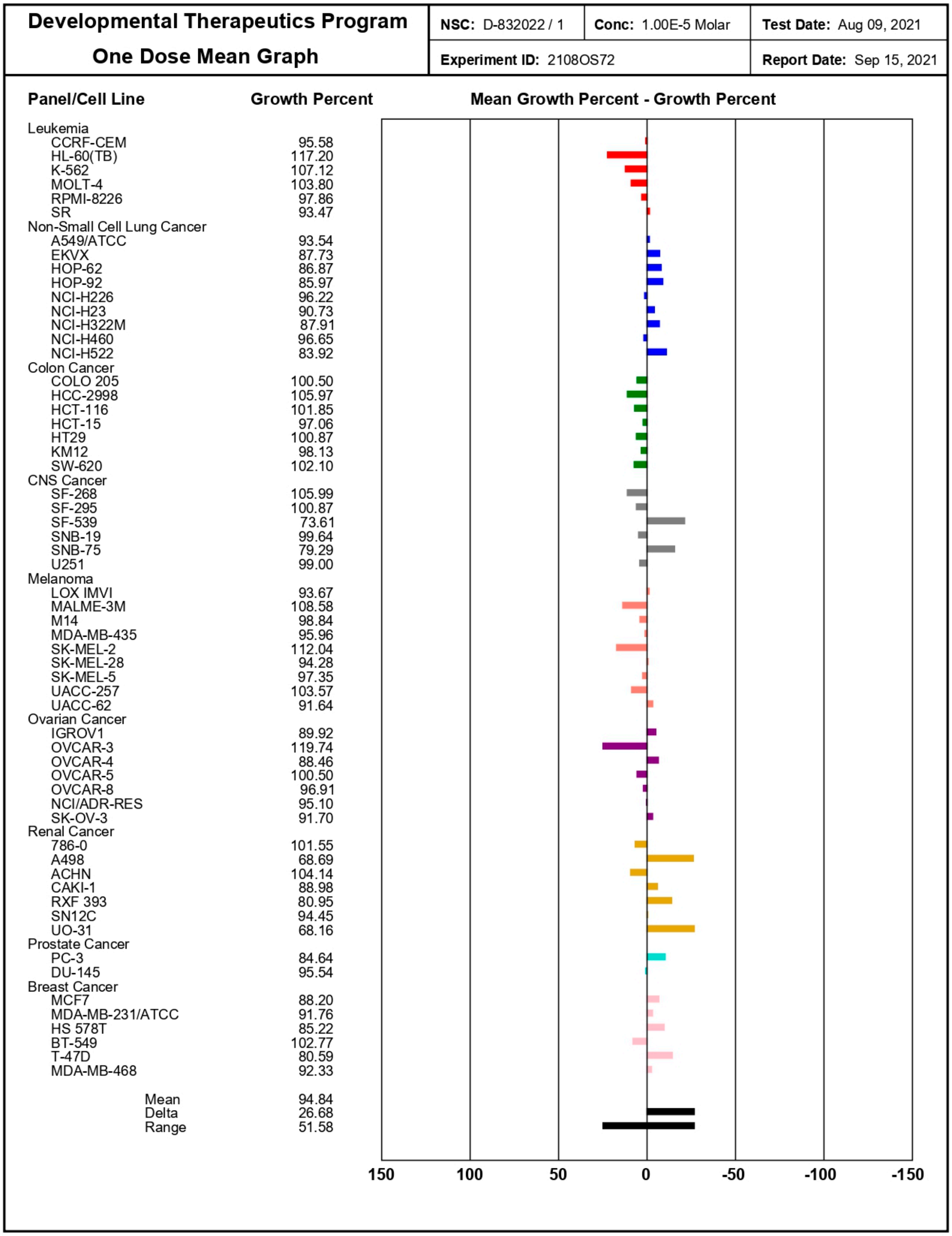

Figure S71. Mean graph of compound (**11b**) with colour codes for each cell line.

Developmental Therapeutics Program One Dose Mean Graph	NSC: D-832023 / 1	Conc: 1.00E-5 Molar	Test Date: Aug 09, 2021
	Experiment ID: 2108OS72		Report Date: Sep 15, 2021

Panel/Cell Line	Growth Percent
**Leukemia**	
CCRF-CEM	104.51
HL-60(TB)	115.81
K-562	116.79
MOLT-4	110.77
RPMI-8226	111.65
SR	103.27
**Non-Small Cell Lung Cancer**	
A549/ATCC	93.45
EKVX	91.99
HOP-62	100.32
HOP-92	93.11
NCI-H226	105.53
NCI-H23	90.29
NCI-H322M	88.49
NCI-H460	101.93
NCI-H522	94.72
**Colon Cancer**	
COLO 205	110.09
HCC-2998	103.05
HCT-116	111.91
HCT-15	98.79
HT29	100.49
KM12	103.25
SW-620	102.31
**CNS Cancer**	
SF-268	103.38
SF-295	100.44
SF-539	93.36
SNB-19	97.54
SNB-75	89.79
U251	97.08
**Melanoma**	
LOX IMVI	97.85
MALME-3M	115.55
M14	118.80
MDA-MB-435	93.85
SK-MEL-2	114.81
SK-MEL-28	95.58
SK-MEL-5	98.11
UACC-257	113.13
UACC-62	89.06
**Ovarian Cancer**	
IGROV1	94.86
OVCAR-3	114.21
OVCAR-4	95.87
OVCAR-5	99.76
OVCAR-8	102.71
NCI/ADR-RES	90.82
SK-OV-3	102.85
**Renal Cancer**	
786-0	100.39
A498	69.73
ACHN	102.39
CAKI-1	90.92
RXF 393	87.03
SN12C	99.53
UO-31	80.62
**Prostate Cancer**	
PC-3	97.93
DU-145	96.50
**Breast Cancer**	
MCF7	99.92
MDA-MB-231/ATCC	96.48
HS 578T	106.49
BT-549	63.85
T-47D	92.15
MDA-MB-468	98.25
Mean	99.22
Delta	35.37
Range	54.95

Mean Growth Percent - Growth Percent

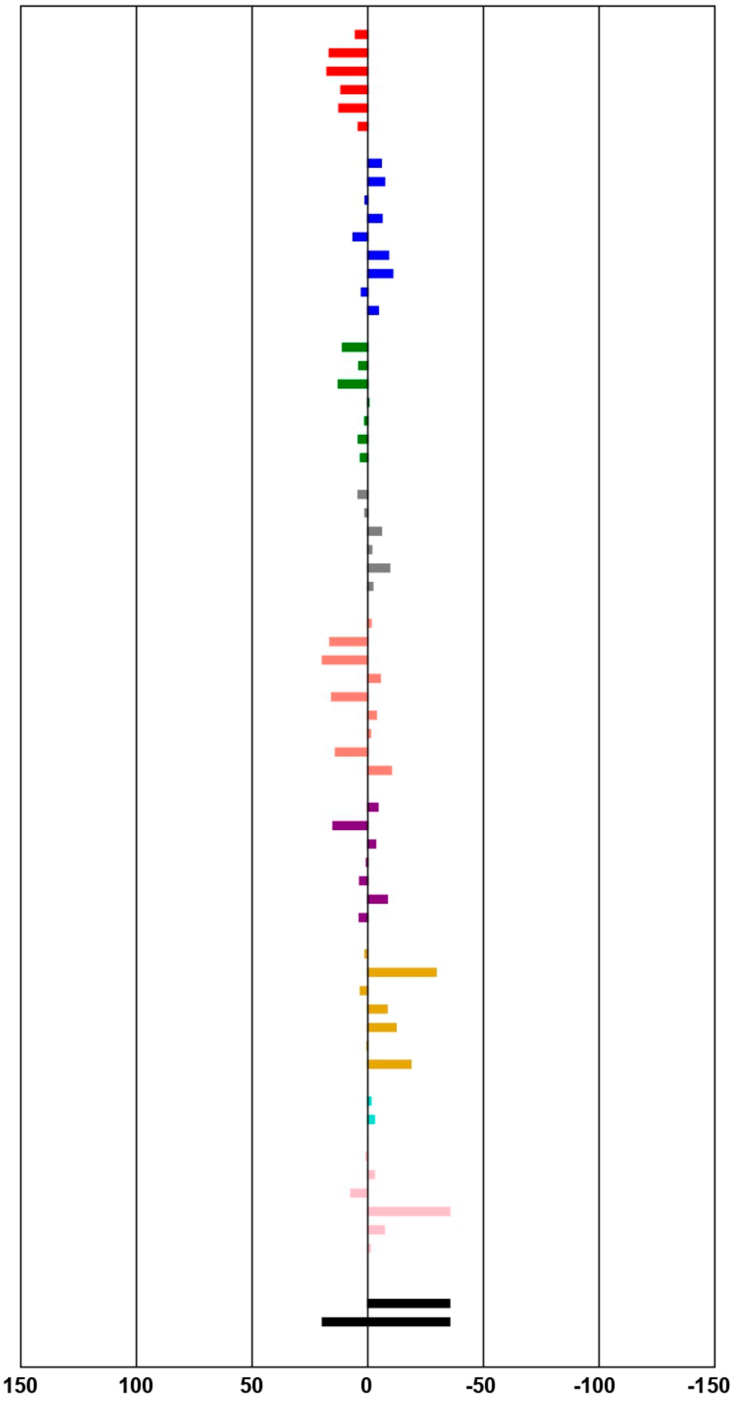

Figure S72. Mean graph of compound (**11c**) with colour codes for each cell line.

Developmental Therapeutics Program One Dose Mean Graph	NSC: D-832024 / 1	Conc: 1.00E-5 Molar	Test Date: Aug 09, 2021
	Experiment ID: 2108OS72		Report Date: Sep 15, 2021

Panel/Cell Line	Growth Percent
**Leukemia**	
CCRF-CEM	95.76
HL-60(TB)	110.33
K-562	109.02
MOLT-4	110.29
RPMI-8226	100.02
SR	103.81
**Non-Small Cell Lung Cancer**	
A549/ATCC	98.04
EKVX	94.93
HOP-62	88.87
HOP-92	84.39
NCI-H226	116.32
NCI-H23	92.41
NCI-H322M	97.23
NCI-H460	95.22
NCI-H522	88.72
**Colon Cancer**	
COLO 205	118.19
HCC-2998	102.51
HCT-116	104.95
HCT-15	98.54
HT29	100.28
KM12	100.71
SW-620	96.58
**CNS Cancer**	
SF-268	109.25
SF-295	99.70
SF-539	97.93
SNB-19	103.59
SNB-75	88.85
U251	95.39
**Melanoma**	
LOX IMVI	94.95
MALME-3M	127.29
M14	118.31
MDA-MB-435	91.38
SK-MEL-2	108.12
SK-MEL-28	97.10
SK-MEL-5	97.16
UACC-257	108.44
UACC-62	93.52
**Ovarian Cancer**	
IGROV1	102.81
OVCAR-3	125.97
OVCAR-4	94.71
OVCAR-5	102.19
OVCAR-8	99.61
NCI/ADR-RES	94.47
SK-OV-3	115.26
**Renal Cancer**	
786-0	104.44
A498	62.34
ACHN	103.29
CAKI-1	93.43
RXF 393	107.91
SN12C	95.62
UO-31	80.33
**Prostate Cancer**	
PC-3	96.10
DU-145	98.29
**Breast Cancer**	
MCF7	94.00
MDA-MB-231/ATCC	92.12
HS 578T	104.32
BT-549	114.74
T-47D	87.54
MDA-MB-468	109.99
Mean	100.30
Delta	37.96
Range	64.95

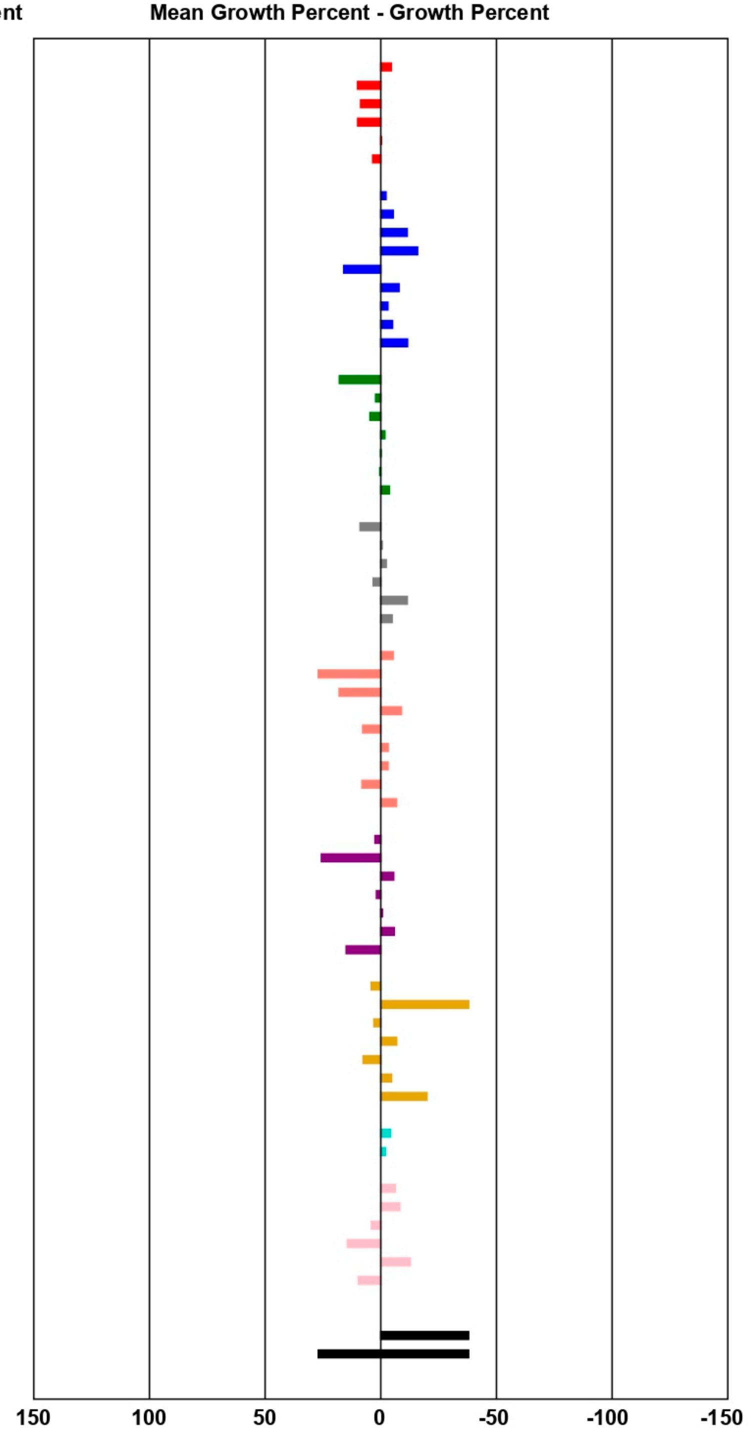

Figure S73. Mean graph of compound (**11d**) with colour codes for each cell line.

Developmental Therapeutics Program One Dose Mean Graph	NSC: D-832025 / 1	Conc: 1.00E-5 Molar	Test Date: Aug 09, 2021
	Experiment ID: 2108OS72		Report Date: Sep 15, 2021

Panel/Cell Line	Growth Percent
**Leukemia**	
CCRF-CEM	96.49
HL-60(TB)	102.38
K-562	95.66
MOLT-4	91.55
RPMI-8226	95.86
SR	83.28
**Non-Small Cell Lung Cancer**	
A549/ATCC	100.31
EKVX	89.52
HOP-62	98.14
HOP-92	78.86
NCI-H226	106.25
NCI-H23	99.87
NCI-H322M	87.45
NCI-H460	98.25
NCI-H522	96.23
**Colon Cancer**	
COLO 205	103.86
HCC-2998	118.44
HCT-116	96.70
HCT-15	94.43
HT29	91.17
KM12	98.92
SW-620	99.80
**CNS Cancer**	
SF-268	109.42
SF-295	92.51
SF-539	102.77
SNB-19	108.03
SNB-75	88.90
U251	110.44
**Melanoma**	
LOX IMVI	99.24
MALME-3M	127.67
M14	110.60
MDA-MB-435	97.23
SK-MEL-2	104.05
SK-MEL-28	97.69
SK-MEL-5	96.31
UACC-257	113.62
UACC-62	91.42
**Ovarian Cancer**	
IGROV1	97.79
OVCAR-3	131.55
OVCAR-4	96.80
OVCAR-5	99.38
OVCAR-8	95.89
NCI/ADR-RES	92.48
SK-OV-3	109.81
**Renal Cancer**	
786-0	101.58
A498	66.58
ACHN	98.97
CAKI-1	97.97
RXF 393	103.43
SN12C	99.67
UO-31	72.90
**Prostate Cancer**	
PC-3	98.93
DU-145	95.86
**Breast Cancer**	
MCF7	94.70
MDA-MB-231/ATCC	95.06
HS 578T	99.90
BT-549	113.28
T-47D	88.28
MDA-MB-468	104.97
Mean	98.80
Delta	32.22
Range	64.97

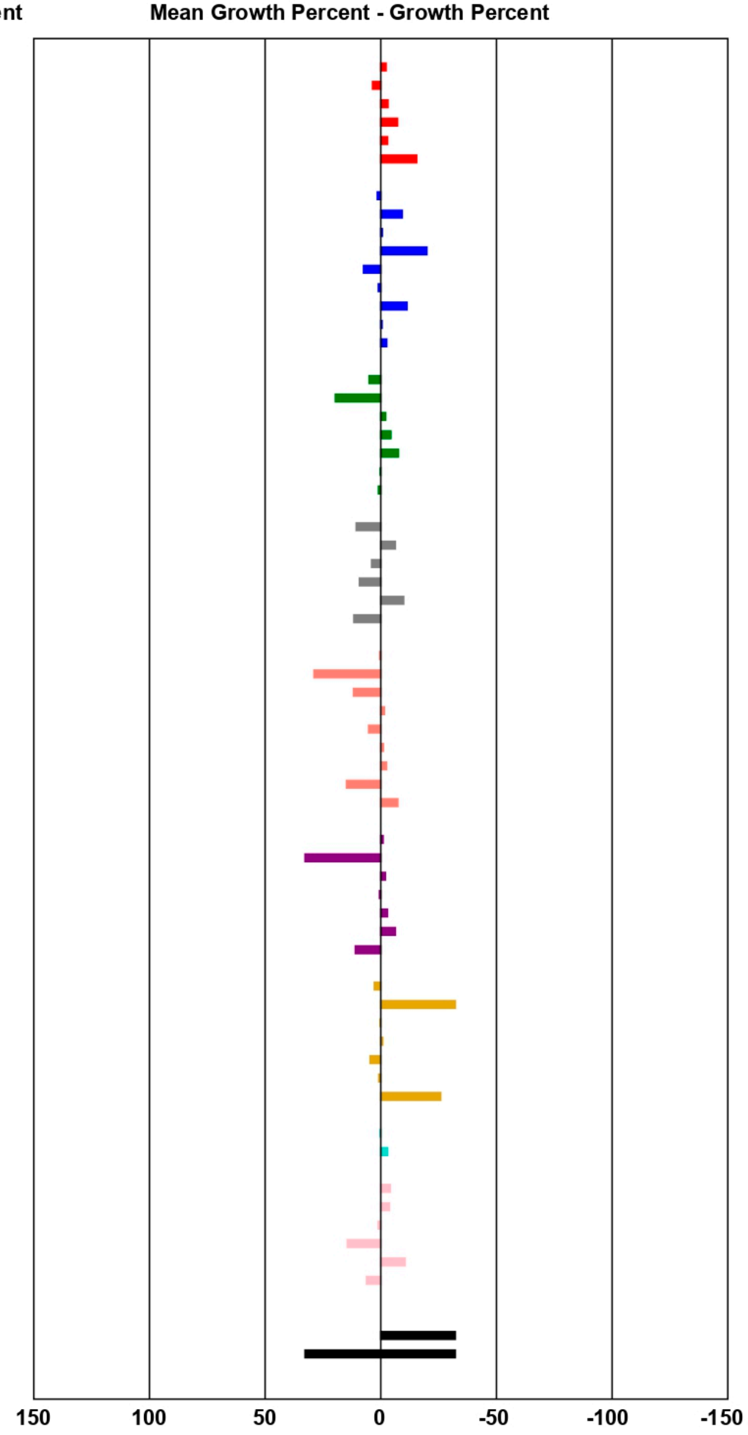

Figure S74. Mean graph of compound (**11e**) with colour codes for each cell line.

Developmental Therapeutics Program One Dose Mean Graph	NSC: D-832026 / 1	Conc: 1.00E-5 Molar	Test Date: Aug 09, 2021
	Experiment ID: 2108OS72		Report Date: Sep 15, 2021

Panel/Cell Line	Growth Percent
Leukemia	
CCRF-CEM	84.19
HL-60(TB)	106.20
K-562	94.07
MOLT-4	104.72
RPMI-8226	92.45
SR	95.21
Non-Small Cell Lung Cancer	
A549/ATCC	96.66
EKVX	89.01
HOP-62	87.98
HOP-92	82.34
NCI-H226	94.00
NCI-H23	90.70
NCI-H322M	90.85
NCI-H460	81.29
NCI-H522	98.01
Colon Cancer	
COLO 205	94.52
HCC-2998	113.39
HCT-116	93.77
HCT-15	99.23
HT29	104.33
KM12	88.37
SW-620	89.66
CNS Cancer	
SF-268	94.11
SF-295	84.53
SF-539	78.35
SNB-19	90.80
SNB-75	48.55
U251	99.37
Melanoma	
LOX IMVI	85.12
MALME-3M	112.56
M14	94.89
MDA-MB-435	90.68
SK-MEL-2	84.69
SK-MEL-28	93.73
SK-MEL-5	94.05
UACC-257	106.74
UACC-62	87.39
Ovarian Cancer	
IGROV1	96.16
OVCAR-3	115.42
OVCAR-4	85.07
OVCAR-5	97.84
OVCAR-8	84.54
NCI/ADR-RES	89.67
SK-OV-3	101.23
Renal Cancer	
786-0	83.32
A498	72.78
ACHN	74.07
CAKI-1	76.93
RXF 393	89.30
SN12C	89.67
UO-31	79.46
Prostate Cancer	
PC-3	96.58
DU-145	93.38
Breast Cancer	
MCF7	88.36
MDA-MB-231/ATCC	93.08
HS 578T	72.21
BT-549	98.38
T-47D	93.81
MDA-MB-468	99.72
Mean	91.31
Delta	42.76
Range	66.87

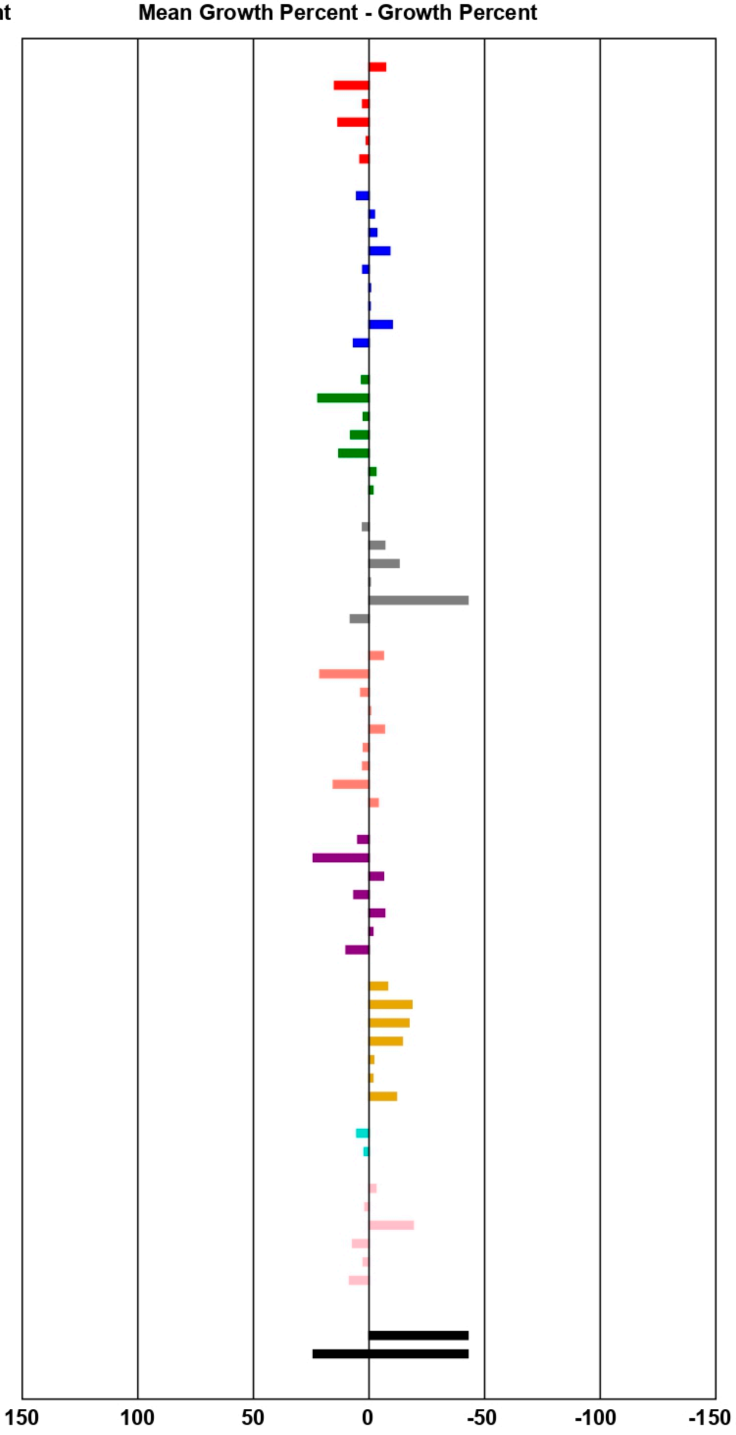

Figure S75. Mean graph of compound (**11f**) with colour codes for each cell line.

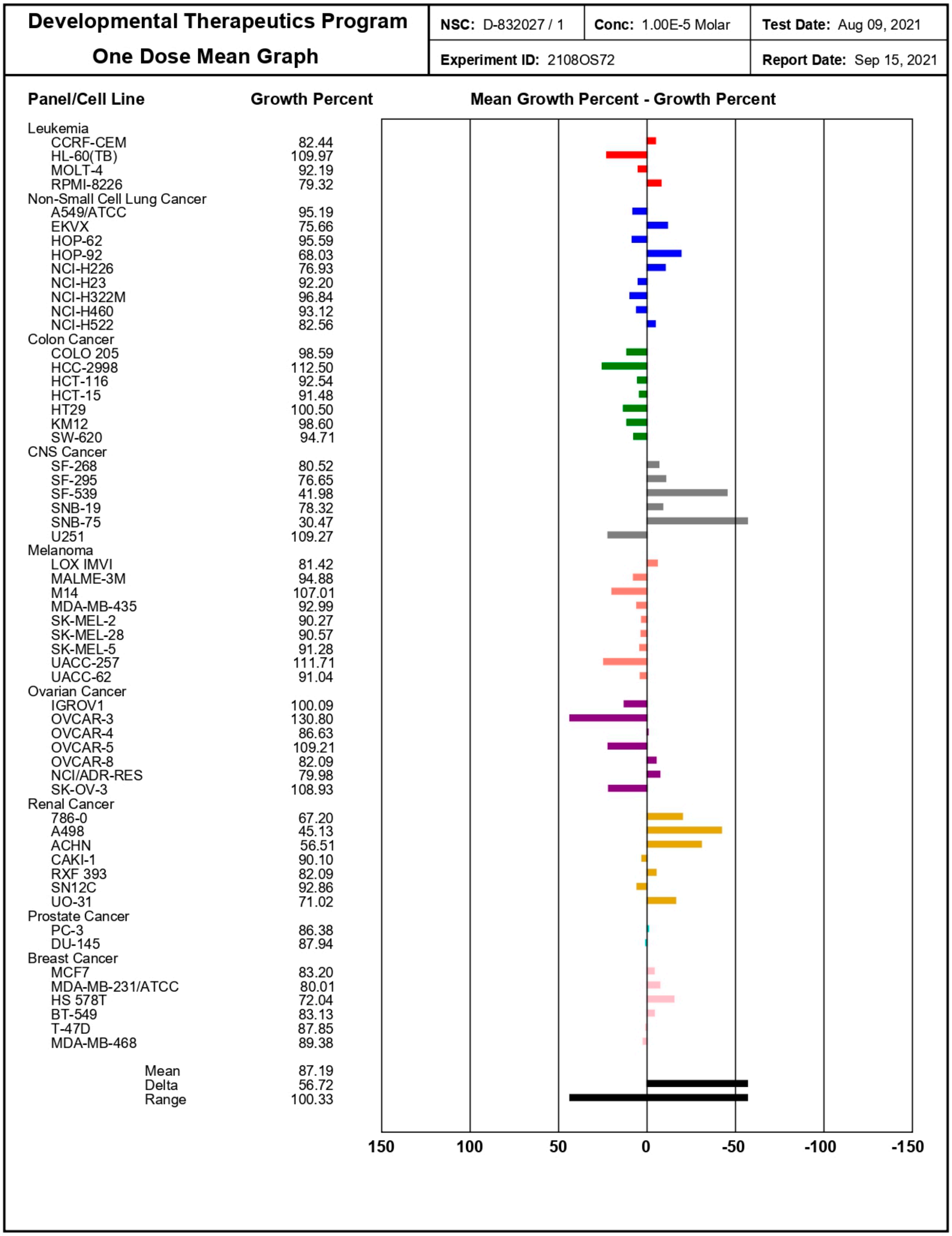

Figure S76. Mean graph of compound (**11g**) with colour codes for each cell line.

Developmental Therapeutics Program One Dose Mean Graph	NSC: D-832028 / 1	Conc: 1.00E-5 Molar	Test Date: Aug 09, 2021
	Experiment ID: 2108OS72		Report Date: Sep 15, 2021

Panel/Cell Line	Growth Percent
Leukemia	
CCRF-CEM	98.17
HL-60(TB)	102.09
K-562	94.89
MOLT-4	101.09
RPMI-8226	96.14
SR	90.34
Non-Small Cell Lung Cancer	
A549/ATCC	106.72
EKVX	97.19
HOP-62	102.03
HOP-92	97.15
NCI-H226	103.90
NCI-H23	101.41
NCI-H322M	95.44
NCI-H460	105.58
NCI-H522	95.04
Colon Cancer	
COLO 205	105.20
HCC-2998	105.07
HCT-116	103.72
HCT-15	101.55
HT29	102.80
KM12	98.01
SW-620	99.26
CNS Cancer	
SF-268	101.99
SF-295	101.40
SF-539	102.06
SNB-19	100.48
SNB-75	97.33
U251	107.55
Melanoma	
LOX IMVI	109.07
MALME-3M	106.63
M14	97.60
MDA-MB-435	93.21
SK-MEL-2	107.05
SK-MEL-28	98.94
SK-MEL-5	99.53
UACC-257	103.25
UACC-62	95.84
Ovarian Cancer	
IGROV1	104.34
OVCAR-3	110.52
OVCAR-4	98.06
OVCAR-5	103.36
OVCAR-8	100.76
NCI/ADR-RES	101.82
SK-OV-3	113.30
Renal Cancer	
786-0	92.76
A498	63.56
ACHN	104.15
CAKI-1	94.38
RXF 393	95.22
SN12C	97.42
UO-31	87.10
Prostate Cancer	
PC-3	99.19
DU-145	105.58
Breast Cancer	
MCF7	90.75
MDA-MB-231/ATCC	103.39
HS 578T	103.66
BT-549	107.09
T-47D	98.91
MDA-MB-468	104.38
Mean	100.07
Delta	36.51
Range	49.74

Mean Growth Percent - Growth Percent

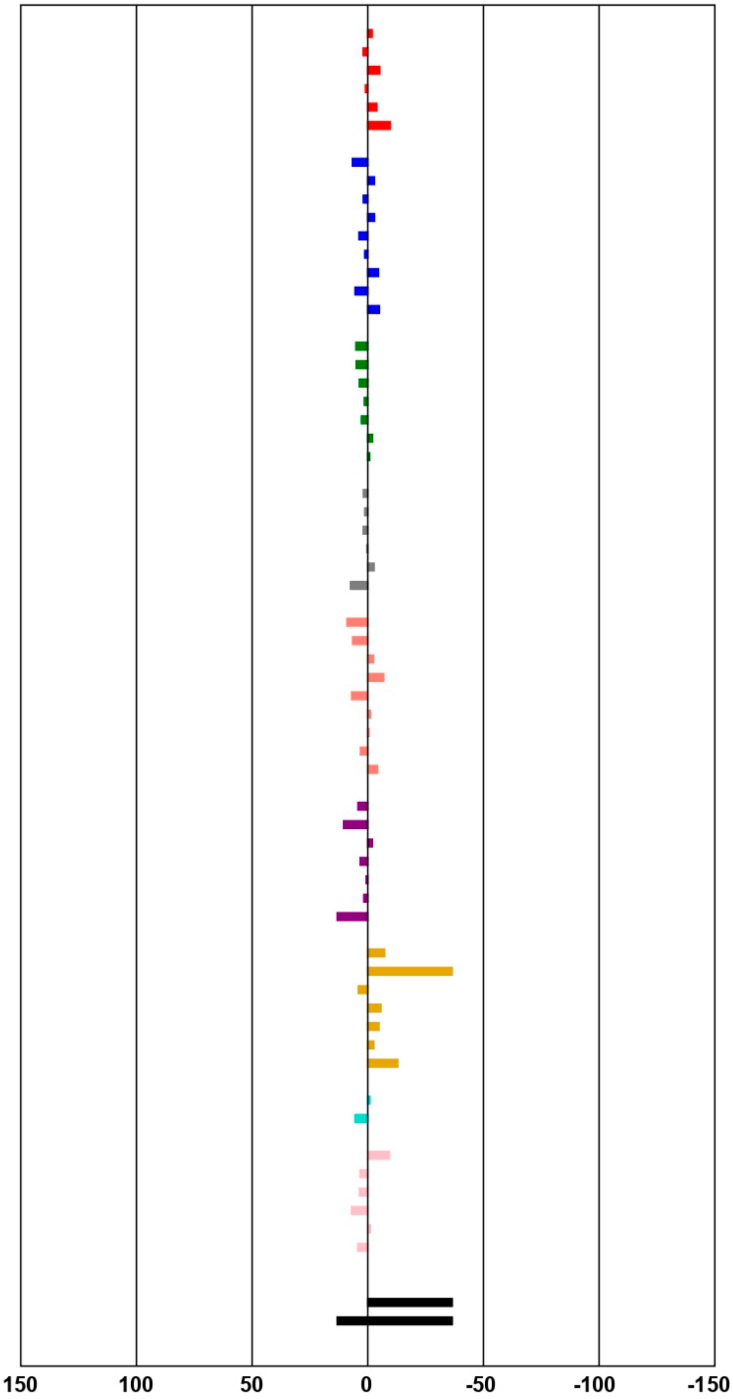

Figure S77. Mean graph of compound (**12**) with colour codes for each cell line.
